# Synergistic lethality between PARP-trapping and alantolactone-induced oxidative DNA damage in homologous recombination-proficient cancer cells

**DOI:** 10.1038/s41388-020-1191-x

**Published:** 2020-02-06

**Authors:** Hongge Wang, Shan Zhang, Liyan Song, Meng Qu, Zhihua Zou

**Affiliations:** Department of Cell Biology and Biophysics, National Engineering Laboratory for AIDS Vaccine, Key Laboratory for Molecular Enzymology and Engineering of the Ministry of Education, School of Life Sciences, Jilin University, Changchun, China

**Keywords:** Mechanisms of disease, Drug development

## Abstract

PARP1 and PARP2 play critical roles in regulating DNA repair and PARP inhibitors have been approved for the treatment of BRCA1/2-mutated ovarian and breast cancers. It has long been known that PARP inhibition sensitizes cancer cells to DNA-damaging cytotoxic agents independent of BRCA status, however, clinical use of PARP inhibitors in combination with DNA-damaging chemotherapy is limited by the more-than-additive cytotoxicity. The natural compound alantolactone (ATL) inhibits the thioredoxin reductase to induce ROS accumulation and oxidative DNA damage selectively in cancer cells. Here, we showed that nontoxic doses of ATL markedly synergized with the PARP inhibitor olaparib to result in synthetic lethality irrespective of homologous recombination status. Synergistic cytotoxicity was seen in cancer but not noncancerous cells and was reduced by the ROS inhibitor NAC or knockdown of OGG1, demonstrating that the cytotoxicity resulted from the repair of ATL-induced oxidative DNA damage. PARP1 knockdown suppressed the synergistic lethality and olaparib was much more toxic than veliparib when combined with ATL, suggesting PARP-trapping as the primary inducer of cytotoxicity. Consistently, combined use of ATL and olaparib caused intense signs of replication stress and formation of double strand DNA breaks, leading to S and G_2_ arrest followed by apoptosis. In vivo, the combination effectively induced regression of tumor xenografts, while either agent alone had no effect. Hence, PARP trapping combined with specific pro-oxidative agents may provide safe and effective ways to broaden the therapeutic potential of PARP inhibitors.

## Introduction

Cancer cells exhibit chronic replication stress, accumulate DNA damage and are highly dependent on compensatory DNA damage response (DDR) functions for survival. Thus, targeting DNA repair and/or DDR has emerged as a promising anticancer approach [1, 2]. Poly(ADP-ribose) polymerase 1 (PARP1), and to a lesser extent PARP2, play critical and overlapping roles in the major DNA repair pathways and in the maintenance of genomic stability [3, 4]. They detect single strand DNA breaks (SSB) and initiate SSB repair (SSBR) [5]; in addition, they are required for a subset of base excision repair (BER) [6] and perform regulatory roles in double strand DNA break (DSB) repair [4]. Importantly, they play critical roles in the stabilization and restart of stalled DNA replication forks [7–9]. Thus, when the activity of PARP1/2 is inhibited, unrepaired SSBs and stalled replication forks accumulate, resulting in collapse of replication forks and generation of DSBs during DNA replication [10], which are repaired by HR-mediated repair (HRR) pathways [3, 4]. However, BRCA1 or BRCA2-null cancer cells are deficient in HR and the problems caused by PARP inhibition become lethal even in the absence of exogenous genotoxic stress [11–13]. These observations have greatly accelerated the development of PARP inhibitors (PARPis) [14, 15]. Several PARPis are now approved for the treatment of BRCA1/2-mutated ovarian and breast cancers [16]. However, as most cancers are HR-proficient, the clinical potential of PARPi as monotherapy is very limited.

It has long been known that inhibition of PARP1/2 sensitizes cancer cells to ionizing radiation and DNA-damaging genotoxic agents independent of HR status [17, 18]. In addition, preclinical and clinical studies have shown that PARP-1 activity in cancer cells is critical for the establishment of resistance to genotoxic therapies [19, 20], suggesting that there is a real opportunity to combine PARPi with chemotherapy and radiotherapy [21, 22]. However, clinical trials designed to test the use of PARPis in combination with chemo/radiotherapy have been unsuccessful, largely due to unexpected more-than-additive side-effects [16].

Increased generation of reactive oxygen species (ROS) is another distinctive feature associated with oncogenic transformation which renders cancer cells vulnerable to further oxidative insult [23]. Thus, agents that weaken the antioxidant systems or promote generation of ROS can induce oxidative DNA damage selectively in cancer cells [24–27]. PARP1/2 are required for the repair of oxidative DNA lesions [28–30] and it has been shown that PARP inhibition sensitizes cells to oxidative stress [31–33], raising the possibility of using PARPis in combination with pro-oxidative agents to yield cancer-specific synergistic lethality. The natural compound alantolactone (ATL) increases cellular ROS levels by inhibiting the thioredoxin reductase (TrxR) [34–37]. We and others have shown that ATL-induced ROS elevation resulted in extensive DNA damage selectively in cancer cells [37, 38]. Here we showed that PARP-trapping caused by the PARPi olaparib markedly synergized with non-toxic doses of ATL to result in cancer-specific lethality and co-administration of sublethal doses of olaparib and ATL effectively induced regression of tumor xenografts in vivo. These studies support further exploration of synergistic lethality between PARP trapping and specific pro-oxidative agents in order to use PARP inhibitors in the treatment of cancer irrespective of HR status.

## Results

### ATL-induced oxidative DNA damage activates PARP in cancer cells

**Fig. 1:**
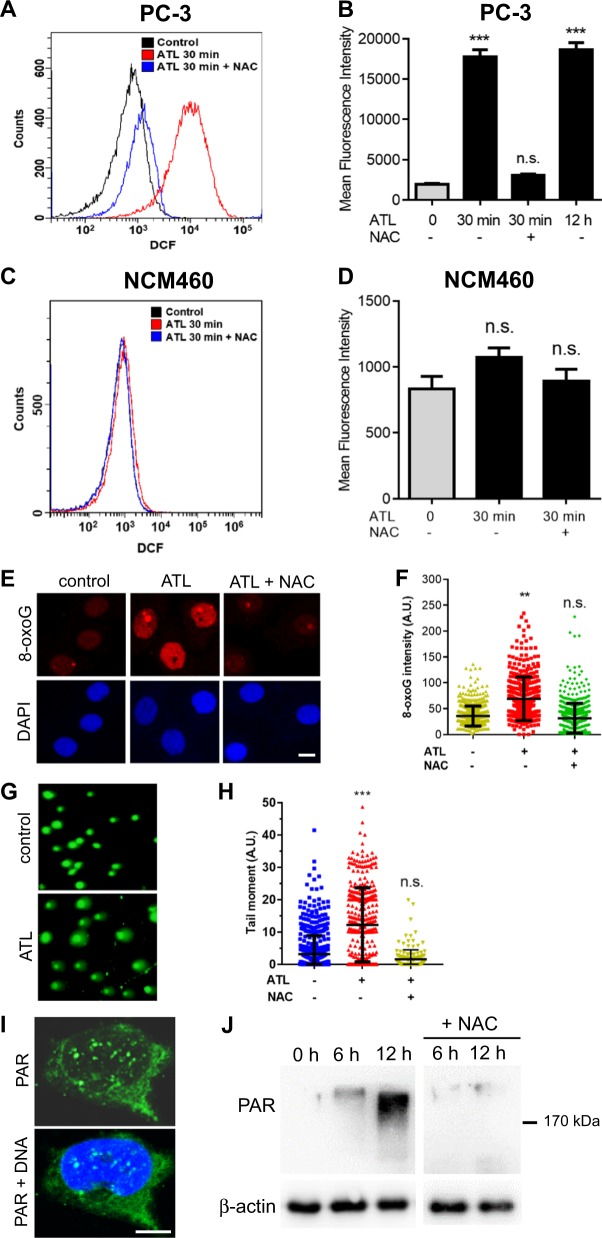
A nontoxic dose of ATL induces oxidative DNA damage and activates PARP in cancer cells. The PC-3 prostate cancer and NCM460 normal colon epithelial cells, with or without 1 h of preincubation in 10 mM NAC, were treated by 10 μM ATL or vehicle control for the indicated times. **a**, **b** Measurement of ROS in PC-3 cells by flow cytometry. Data from three independent experiments were presented as mean ± SD. **c**, **d** Measurement of ROS in NCM460 cells by flow cytometry. **e**, **f** Immunofluorescent staining of cellular 8-oxoG by Cy3-conjugated avidin. PC-3 cells were treated by 10 μM ATL for 12 h (scale bar: 10 μm). Nuclear 8-oxoG intensity was measured using the ImageJ software and the data were processed by the Prism software. **g** Representative images of alkaline comet assay. PC-3 cells were treated by vehicle control or 10 μM ATL for 12 h. **h** The tail moment was defined as percentage of tail DNA × tail length, quantified using the TriTek CometScore software. **i** Immunofluorescence staining for PAR foci in PC-3 cells treated by 10 μM ATL for 12 h. DNA was counterstained with DAPI (scale bar: 5 μm). **j** Western blot analysis of PAR in PC-3 cells. Ten micrometer ATL resulted in a time-dependent increase in PAR levels which was blocked by NAC. *n.s.* not significant, **p < 0.01, ***p < 0.001 vs. vehicle control.

Tumor cell toxicity is a well-known property of ATL, but the effective concentrations reported in the literature were very high, with IC_50_ values ranging from 20 to 60 μM in various solid tumor cell lines [38–40]. In agreement with the literature, we found 10 μM ATL had no significant impact on the clonogenic growth of many human cancer cell lines (Fig. S1A), nevertheless, it induced a marked increase in ROS levels. Within 30 min of treatment, ROS levels increased by 10-fold in the PC-3 prostate cancer cells (Fig. 1a, b) and remained unchanged until the 12 h time point (Fig. 1b). A rapid increase in ROS levels was also induced by 10 μM ATL in the SW480 colorectal and A549 lung carcinoma (Fig. S1B) as well as many other cancer cell lines (Fig. S1C). Lowering the concentrations of ATL to as low as 0.625 μM could still induce a significant increase in ROS levels, and the ROS increase induced by the nontoxic concentrations of ATL in the PC-3 and SW480 cancer cells exhibited optimal dose-dependency (Fig. S1D). In contrast, similar treatment caused no significant ROS elevation in the noncancerous NCM460 normal colon (Fig. 1c, d), BEAS-2B bronchial and HEK293 embryonic kidney epithelial cell lines (Fig. S1C). The ROS inhibitor *N*-acetyl-l-cysteine (NAC) efficiently blocked the ATL-induced ROS increase in the cancer cells (Fig. 1a, b and Fig. S1B). Cancer cells characteristically have higher oxidative pressure and upregulate antioxidant systems to combat the toxicity of excessive levels of ROS [26, 27]. As ATL is known to inhibit thioredoxin reductase (TrxR) [34], our results suggested that the thioredoxin antioxidant system likely plays critical roles in most cancer cells. On the other hand, normal cells may better tolerate TrxR inhibition owing to their normal basal ROS output.

At elevated levels, ROS can cause SSBs and oxidize nucleobases in DNA/RNA and free nucleotides [41, 42]. We found that the ATL-induced ROS elevation in the PC-3, SW480 and A549 cancer cells was followed by accumulation of cellular 8-oxoguanine (8-oxoG) (Fig. 1e, f and Fig. S2A) and an increase in DNA strand breaks as revealed by the alkaline comet assay (Fig. 1g, h and Fig. S2B). Both effects were reversed by NAC (Fig. 1e–h and Fig. S2A-B), suggesting that endogenous oxidative pressure in cancer cells was responsible for these oxidative DNA lesions following inhibition of the thioredoxin antioxidant system by ATL.

PARP1/2 sense and bind SSBs produced directly or as intermediates of BER repair of certain types of damaged DNA bases [3]. This stimulates the catalytic activity of PARP1/2, which results in rapid synthesis of poly(ADP-ribose) (PAR) chains (PARylation) on proteins at sites of SSB. Consistent with the increase in cellular 8-oxoG levels and DNA strand breaks, PAR levels increased in a time-dependent manner in ATL-treated cancer but not the NCM460 and BEAS-2B cells (Fig. 1i, j and Fig. S2C). NAC treatment reduced the accumulation of PAR in the cancer cells (Fig. 1j and Fig. S2C), correlating the PARylation with oxidative DNA damage. These results revealed that a nontoxic dose of ATL caused oxidative DNA damage specifically in cancer cells which activated PARP-mediated DNA repair activity.

### ATL sensitizes HR-proficient cancer cells to olaparib

**Fig. 2:**
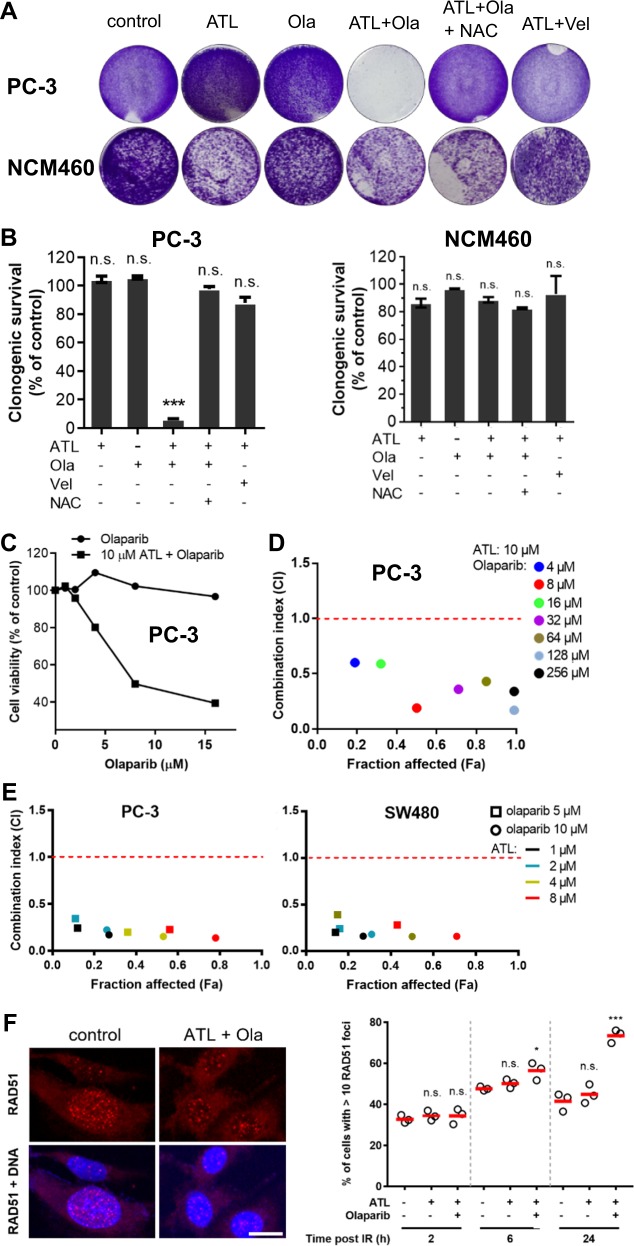
Olaparib and ATL synergize to result in synthetic lethality in HR-proficient cancer cells. **a** Colony formation assay. PC-3 and NCM460 cells were treated by 10 μM ATL, 10 μM olaparib (Ola), or combination of 10 μM ATL and 10 μM Ola or 10 μM ATL and 10 μM veliparib (Vel) for 7 days. The combination of 10 μM ATL and 10 μM Ola, but not 10 μM ATL and 10 μM Vel, completely inhibited the clonogenic growth of PC-3 but not the NCM460 cells and 10 mM NAC blocked the inhibition. **b** Quantification of colony formation assay. Cells stained by crystal violet were dissolved in 70% ethanol and absorbance at 595 nm was measured using a microplate reader. Data were presented as mean ± SD of three independent experiments. **c** MTT proliferation assay. PC-3 cells were treated by 1, 2, 4, 8, or 16 μM olaparib alone or combined with 10 μM ATL for 72 h. Olaparib in combination with 10 μM ATL dose-dependently inhibited the growth of PC-3 cells, while olaparib alone had no impact. **d** Determination of combination index (CI) values. PC-3 cells were treated by 10 μM ATL and the indicated concentrations of olaparib for 72 h. The CI values were determined by the Chou-Talalay method using the CompuSyn software. **e** CI values between lower concentrations of ATL and olaparib. PC-3 or SW480 cells were treated by 5 or 10 μM olaparib and the indicated concentrations of ATL for 72 h. **f** RAD51 foci formation after ionizing radiation (IR) exposure. PC-3 cells were irradiated with 3 Gy X-rays, treated with vehicle control, 10 μM ATL alone or in combination with 10 μM olaparib (Ola), and immunostained at the indicated time points. Left: representative micrographs of PC-3 cells stained with anti-RAD51 and counterstained with DAPI 2 h post IR (scale bar: 10 μm). Right: quantification of the percentage of cells with more than 10 RAD51 foci at the indicated time points post IR. *n.s.* not significant, *p < 0.05, ***p < 0.001 vs. vehicle control.

The increase in PAR levels prompted us to ask if nontoxic doses of ATL could sensitize cancer cells to PARPi. We first assessed the cytotoxicity of the PARPi olaparib in the PC-3, A549, and SW480 cancer cell lines and found the IC_50_ to be 46.07, 35.69, and 91.06 μM, respectively (Fig. S3A). Consistently, both 10 μM ATL and 10 μM olaparib did not affect the clonogenic growth of these cancer cell lines but remarkably, the combination of 10 μM ATL and 10 μM olaparib completely inhibited their clonogenic survival (Fig. 2a, b and Fig. S3B). Similarly, MTT proliferation assay showed that, in the presence of 10 μM ATL, olaparib dose-dependently inhibited the viability of the PC-3, A549, and SW480 cancer cells while olaparib monotherapy was nontoxic (Fig. 2c and Fig. S3C). In stark contrast, the clonogenicity of the noncancerous NCM460 (Fig. 2a, b) and BEAS-2B (Fig. S3B) cell lines was not affected by the combination of 10 μM ATL and 10 μM olaparib.

To evaluate the nature of the additive cytotoxicity between ATL and olaparib, we determined the combination index (CI) by the Chou-Talalay method using the CompuSyn software [43]. The CI values for the combinations between 10 μM ATL and a series of olaparib concentrations indicated synergism (CI < 1) in all three cancer cell lines (Fig. 2d and Fig. S3D). Over a wide range of olaparib concentrations, CI numbers were far below 0.5, and notably, the CI at the 50% fraction affected (Fa) level was 0.19, 0.30, and 0.35 in the PC-3, SW480, and A549 cell line respectively, indicating strong synergy between ATL and olaparib. As ROS increase could also be induced by lower concentrations of ATL in cancer cells, CI values between a series of lower concentrations of ATL and 5 or 10 μM olaparib were determined in PC-3 and SW480 cancer cells, which were all well below 0.5, indicating that strong synthetic lethality could be achieved at lower ATL concentrations (Fig. 2e).

It is well established that HR deficiency sensitizes cells to PARP inhibition, and factors other than BRCA1/2 mutations can induce HR defects [44, 45]. To check if the ATL-induced PARPi sensitization was resulted from HR deficiency, we examined the HR status by checking RAD51 foci formation [46] and by monitoring the repair of DSBs using the DR-GFP reporter [47]. We found that RAD51 readily formed ionizing radiation-induced foci in PC-3, SW480 and A549 cells (Fig. 2f and Fig. S4A). Likewise, RAD51 foci were effectively induced by 0.1 μM cisplatin in A549 cells (Fig. S4B). The number of RAD51 positive cells were not changed or were increased by ATL or the combination of ATL and olaparib over a 24 h time period (Fig. 2f and Fig. S4A, B), indicating that the PC-3, A549, and SW480 cancer cells were able to efficiently assemble recombination filaments, and 10 μM ATL alone or in combination with 10 μM olaparib did not reduce the recombination capacity. To directly assay HR proficiency, we established stable PC-3 and A549 cell lines harboring the DR-GFP reporter and co-transfected these cells with vectors expressing I-SceI and RFP. Twenty-four hours after transfection, cells were treated with drugs or vehicle control for 12 h. The results showed that nearly all red cells (indicating expression of I-SceI and RFP) overlapped with green cells (indicating recovery of functional GFP gene through HRR) (Fig. S4C), and flow cytometry analyses showed that the numbers of green and red cells were near identical (Fig. S4C) both before and after treatment by the combination of ATL and olaparib, suggesting normal HRR efficiency. Thus, HR-proficient cancer cells were sensitized to olaparib by a nontoxic dose of ATL. Finally, the cytotoxicity of olaparib against the BRCA1-deficient MDA-MB-436 breast cancer cells [48] was further significantly enhanced by 2 and 4 μM ATL (Fig. S4D), suggesting that ATL sensitized cancer cells to olaparib through mechanisms other than inducing HR deficiency.

### Synergy results from the repair of oxidative DNA damage and PARP-trapping

**Fig. 3:**
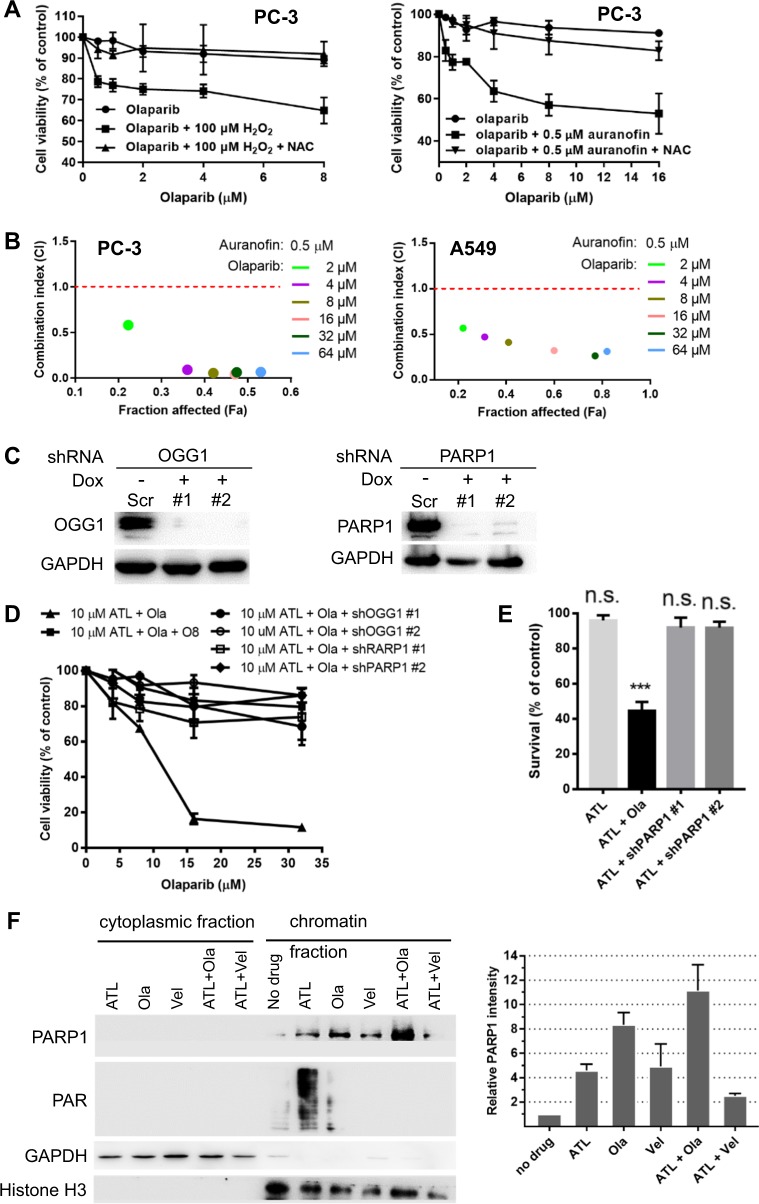
Synergy between olaparib and ATL results from PARP-trapping. **a** Both hydrogen peroxide and auranofin sensitize cancer cells to olaparib. PC-3 cells were treated by 1, 2, 4, 8, or 16 μM olaparib alone or combined with 100 μM H_2_O_2_ or 0.5 μM auranofin for 72 h. Olaparib in combination with 100 μM H_2_O_2_ or 0.5 μM auranofin dose-dependently inhibited the growth of PC-3 cells, while olaparib alone had no impact. NAC blocked H_2_O_2_ or auranofin-induced olaparib sensitization. **b** CI values between auranofin and olaparib indicate strong synergy. PC-3 or A549 cells were treated by 0.5 μM auranofin and the indicated concentrations of olaparib for 72 h. **c** Western blot verification of shRNA-mediated knockdown of OGG1 and PARP1 in PC-3 cells. **d** MTT proliferation assay. Wild-type, OGG1-depleted or inhibited by 8 μM O8, and PARP1-depleted PC-3 cells were treated by 2, 4, 8, or 16 μM olaparib in combination with 10 μM ATL for 72 h. **e** MTT assay. Wild-type or PARP1 depleted PC-3 cells were treated by 10 μM ATL or the combination of 10 μM ATL and 10 μM olaparib (Ola) for 72 h. PARP1 knockdown did not sensitize PC-3 cells to 10 μM ATL. **f** Detection of chromatin-bound PARP1 by Western blot. PC-3 cells were treated by 10 μM ATL, 10 μM olaparib (Ola), 10 μM veliparib (Vel), or the combination of 10 μM ATL with 10 μM olaparib or veliparib for 24 h. *n.s.* not significant, ***p < 0.001 vs. vehicle control.

The strong synergism between ATL and olaparib inspired us to explore the genetic basis behind it. At first, it was found that the synergistic cytotoxicity was suppressed by NAC (Fig. 2a, b and Fig. S3B), suggesting that it was dependent on the presence of oxidative DNA damage. Supporting this view, both hydrogen peroxide (H_2_O_2_) and another TrxR inhibitor auranofin [49], significantly potentiated the cytotoxicity of olaparib in PC-3 cells (Fig. 3a), and the CI values between 0.5 μM auranofin and a series of olaparib concentrations in PC-3 and A549 cancer cells also indicated strong synergistic interactions between auranofin and olaparib (Fig. 3b).

The major product of oxidative DNA damage is 8-oxoG which is repaired by the 8-oxoguanine glycosylase (OGG1)-initiated BER [42]. OGG1 exercises oxidized guanine bases and further cleaves the DNA backbone, generating SSBs. PARP1/2 bind the SSBs and recruit XRCC1 to assemble the repair machinery [3]. Thus, inhibition of PARP1/2 or XRCC1 would interfere with the completion of the repair process after 8-oxoG excision, leading to accumulation of SSBs and the potential of forming synergistic cytotoxicity with DNA damaging agents; on the other hand, inhibition of OGG1 would suppress the formation of SSB and mitigate the impact of PARP or XRCC1 inhibition. Indeed, shRNA-mediated knockdown of OGG1, or co-incubation with the OGG1 inhibitor O8, significantly improved cell viability of the combination treatment group (Fig. 3c, d and Fig. S5A, B), indicating that a significant portion of the synergistic cytotoxicity resulted from DNA base excision repair of ATL-induced 8-oxoG.

Unexpectedly, knockdown of PARP1 did not synergize with 10 μM ATL to yield synergistic cytotoxicity (Fig. 3c, e and Fig. S5A, C); on the contrary, PARP1 depletion greatly reduced the cytotoxicity of the ATL and olaparib combination (Fig. 3d and Fig. S5B). These results indicated that inhibition of PARP enzymatic activity was not important but the presence of the PARP1 protein was required for the cytotoxicity of the ATL and olaparib combination, thus supporting PARP-trapping as the principle mechanism underlying the synergy between ATL and olaparib.

Olaparib and veliparib are two different PARP inhibitors with equivalent enzymatic inhibition potency. However, olaparib is substantially more potent than veliparib at trapping PARP on damaged DNA [14, 15]. Consistently, in the presence of ATL, olaparib but not veliparib, greatly increased accumulation of PARP1 protein in the chromatin-bound fraction (Fig. 3f and Fig. S5D). And similar to PARP1 knockdown, veliparib did not synergize with ATL to inhibit the clonogenic survival of the PC-3, SW480 and A549 cancer cells (Fig. 2a, b and Fig. S3B), despite that both veliparib and olaparib efficiently suppressed PAR accumulation (Fig. 3f and Fig. S5D). Taking together, these results showed that the synergy between ATL and olaparib resulted primarily from the formation of trapped PARP-DNA complexes during the repair of ATL-induced oxidative DNA damage.

### Trapped PARP-DNA complexes induce intense replication stress and DSB

**Fig. 4:**
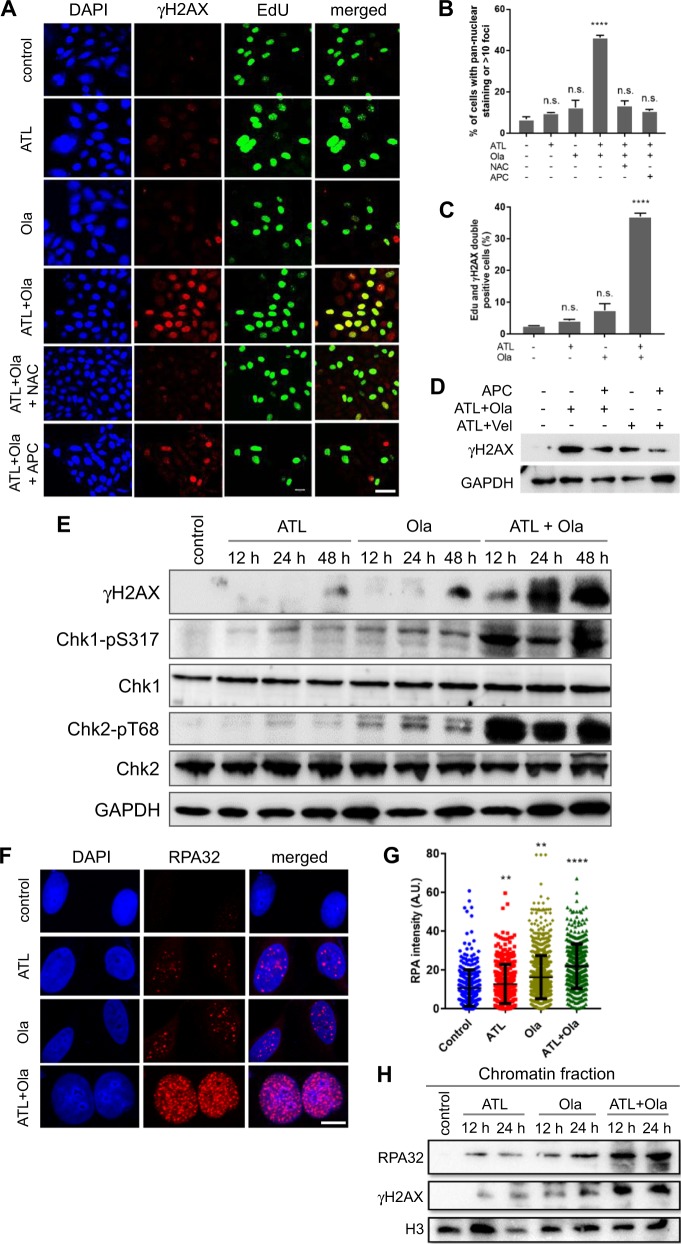
ATL synergizes with olaparib to induce intense replication stress in cancer cells. **a** Immunofluorescent staining of γH2AX and EdU. PC-3 cells were treated by 10 μM ATL, 10 μM olaparib (Ola) or the combination of the two, with or without 10 mM NAC or 5 μM aphidicolin (APC), for 12 h. At the end of drug treatment, cells were pulse-labeled with 10 µM EdU for 20 min (scale bar: 20 μm). **b**, **c** γH2AX and EdU positive cells were measured using the ImageJ software and the data were processed by the Prism software. **d** Western blot detection of γH2AX. PC-3 cells were treated by the combination of 10 μM ATL and 10 μM Ola or 10 μM ATL and 10 μM veliparib (Vel), with or without 5 μM aphidicolin (APC), for 24 h. **e** Western blot analysis of the indicated proteins. PC-3 cells were treated by 10 μM ATL, 10 μM olaparib (Ola) or the combination of the two for the indicated times. **f** Immunofluorescent staining of RPA32 foci. PC-3 cells were treated by 10 μM ATL, 10 μM olaparib (Ola) or the combination of the two for 12 h (scale bar: 10 μm). **g** Nuclear RPA32 intensity was measured using the ImageJ software and the data were processed by the Prism software. **h** Western blot detection of chromatin bound RPA32 and γH2AX. PC-3 cells were treated by 10 μM ATL, 10 μM olaparib (Ola) or the combination of the two for the indicated times. *n.s.* not significant, **p < 0.01, ****p < 0.0001 vs. vehicle control.

Next, we assessed the molecular consequences caused by the synergistic interactions between olaparib and ATL. Treatment by the combination of ATL and olaparib for 12 h, but not by each agent alone, resulted in a marked increase in the number of cancer cells with high-intensity, pan-nuclear γH2AX staining as well as the total number of γH2AX positive cancer cells (Fig. 4a, b and Fig. S6A), indicating induction of intense replication stress [50] and/or DSBs [51] specifically by the combination of ATL and olaparib. The staining intensity of γH2AX was significantly reduced by NAC (Fig. 4a, b and Fig. S6A), correlating it with oxidative DNA damage. Pulse-labeling of DNA replicating cells by EdU showed that most γH2AX positive cells were co-labeled by EdU (Fig. 4a–c and Fig. S6A), and the DNA replication inhibitor aphidicolin significantly reduced γH2AX staining (Fig. 4a, b and Fig. S6A) and γH2AX protein levels (Fig. 4d), suggesting that induction of γH2AX was highly specific to S-phase cells likely as a result of collision between DNA replication forks and the trapped PARP-DNA complexes. The combination of veliparib and ATL induced a much smaller increase in γH2AX levels (Fig. 4d), implicating inhibition of PARP enzymatic activity as a weak inducer of γH2AX in ATL-treated cells.

**Fig. 5:**
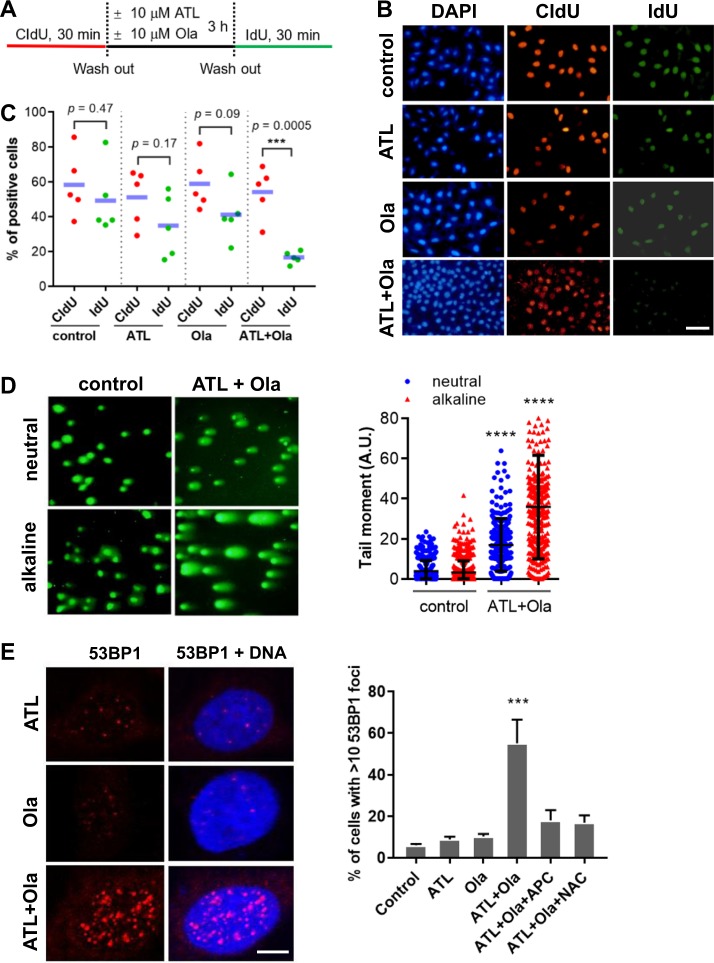
ATL synergizes with olaparib to induce replication fork stalling and DSB in cancer cells. **a** Schedule of CIdU and IdU labeling and drug treatment. **b** Representative images of CIdU and IdU positive PC-3 cells (scale bar: 25 μm). **c** The percentage of CIdU and IdU positive cells in three wells. **d** Representative images of neutral and alkaline comet assay. PC-3 cells were treated by vehicle control or the combination of 10 μM ATL and 10 μM olaparib (Ola) for 12 h. **e** Immunofluorescent staining of 53BP1. PC-3 cells were treated by 10 μM ATL, 10 μM olaparib (Ola) or the combination of the two, with or without 10 mM NAC or 5 μM aphidicolin (APC), for 12 h. 53BP1 positive cells were measured using the ImageJ software and the data were processed by the Prism software. ***p < 0.001, ****p < 0.0001 vs. vehicle control.

Consistent with the strong and time-dependent induction of γH2AX (Fig. 4e and Fig. S6B), protein levels of phosphorylated Chk1 and Chk2 were significantly increased by the combination of ATL and olaparib (Fig. 4e and Fig. S6B), demonstrating strong activation of both ATR-Chk1 and ATM-Chk2 DNA damage response pathways [52]. Furthermore, the amounts of chromatin-bound replication protein A (RPA) were markedly increased (Fig. 4f–h and Fig. S6C) in the combination treatment group, indicating mass production of single-stranded DNA (ssDNA), a sign of replication fork stalling and intense replication stress [53]. In addition, we labeled the PC-3 cells by CIdU for 30 min right before a 3-h drug treatment, and then by IdU for 30 min immediately after drug treatment (Fig. 5a). Almost the same population of cells were labeled by CIdU and IdU in the control and mono-agent treatment groups, however, compared to CIdU labeled cells, there was a sharp drop in the number of IdU positive cells as well as IdU staining intensity in the drug combination group (Fig. 5b, c), reflecting severe replication fork stalling.

Running the comet assay under a neutral condition, we found that a significant amount of DSBs were accumulated in the cancer cells treated by the combination of ATL and olaparib for 12 h (Fig. 5d and Fig. S7A). Bigger and longer comet tails were revealed by the alkaline comet assay (Fig. 5d and Fig. S7A), indicating the presence of a large number of SSBs. Moreover, 53BP1 foci, which represent sites of double strand DNA breaks [54], were markedly increased in the cancer cells treated by the combination of ATL and olaparib for 12 h and both NAC and the DNA replication inhibitor aphidicolin dramatically reduced the number of 53BP1 positive cells (Fig. 5e and Fig. S7B).

### Activation of DNA damage response leads to apoptosis after G_2_ arrest

**Fig. 6:**
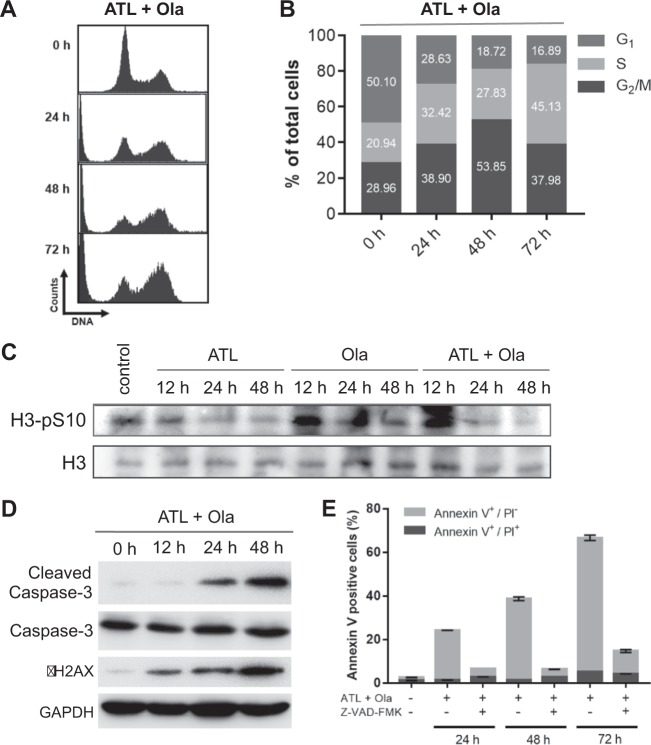
Induction of cell cycle arrest and apoptosis by ATL and olaparib. **a**, **b** Flow cytometry analysis of cell cycle distribution. Treatment by the combination of 10 μM ATL and 10 μM olaparib (Ola) caused progressive accumulation of PC-3 cells in the S and G_2_/M phases within the first 48 h, and then the G_2_/M but not the S population started to fall, meanwhile, the sub-G_1_ population increased steadily. **c** Western blot analysis of phosphorylated H3. The levels of H3-pS10 in PC-3 cells decreased sharply after treatment by the combination of 10 μM ATL and 10 μM olaparib (Ola) for 12 h and longer, indicating that the cells in the G_2_/M population were in early-to-mid G_2_ phase. **d** Western blot analysis of cleaved caspase 3. Treatment by the combination of 10 μM ATL and 10 μM olaparib (Ola) caused a rapidly progressive increase in the levels of cleaved caspase 3 in the PC-3 cell line. **e** Flow cytometry analysis of apoptosis in PC-3 cells. Treatment by the combination of 10 μM ATL and 10 μM olaparib (Ola) caused a time-dependent increase in Annexin V-positive cells, most of which were propidium iodide (PI) negative, indicating they were in early apoptosis. The pan-caspase inhibitor Z-VAD-FMK suppressed the increase in Annexin V-positive cells.

Given the intense replication stress and strong activation of both Chk1 and Chk2, we assessed cell cycle distribution in response to treatment by the combination of ATL and olaparib. Flow cytometry analyses showed that there was a fast, progressive accumulation of cells in the S and G_2_/M phases within the first 48 h of treatment (Fig. 6a, b and Fig. S8A). As the levels of phosphorylated histone H3 decreased sharply (Fig. 6c and Fig. S8B), suggesting the cells in the G_2_/M population were mainly in early-to-mid G_2_ phase [55], these results indicated activation of both the S and the G_2_/M cell cycle checkpoints. Interestingly, as the treatment continued beyond 48 h, the G_2_ but not the S population, started to shrink, showing a significant reduction by the end of 72 h of treatment (Fig. 6a, b and Fig. S8A); meanwhile, the sub-G_1_ population increased dramatically (Fig. 6a and Fig. S8A), suggesting increased cell death after G_2_ arrest.

To check if the cell death was caused by apoptosis, we tracked the dynamic changes of cleaved caspase 3 by Western blot and Annexin V-positive cells by flow cytometry in response to treatment by the combination of ATL and olaparib. The results showed a rapidly progressive increase in the levels of cleaved caspase 3 (Fig. 6d and Fig. S8C) and in the size of the Annexin V-positive cell population (Fig. 6e and Fig. S8D). By the end of 72 h of treatment, 67% of the PC-3 cancer cells were positive for Annexin V-FITC, however, most of them were propidium iodide (PI) negative, indicating they were in early apoptosis, a sign of rapid induction and turnover of apoptotic cells. Treatment by the pan-caspase inhibitor Z-VAD-FMK suppressed the increase in Annexin V-positive cells (Fig. 6e and Fig. S8D), confirming the presence of caspase-dependent apoptosis. Together, these results showed that the synergistic cytotoxicity of ATL and olaparib resulted from S and G_2_ cell cycle arrest and subsequent induction of apoptosis.

### Coadministration of ATL and olaparib suppresses tumor growth in vivo

**Fig. 7:**
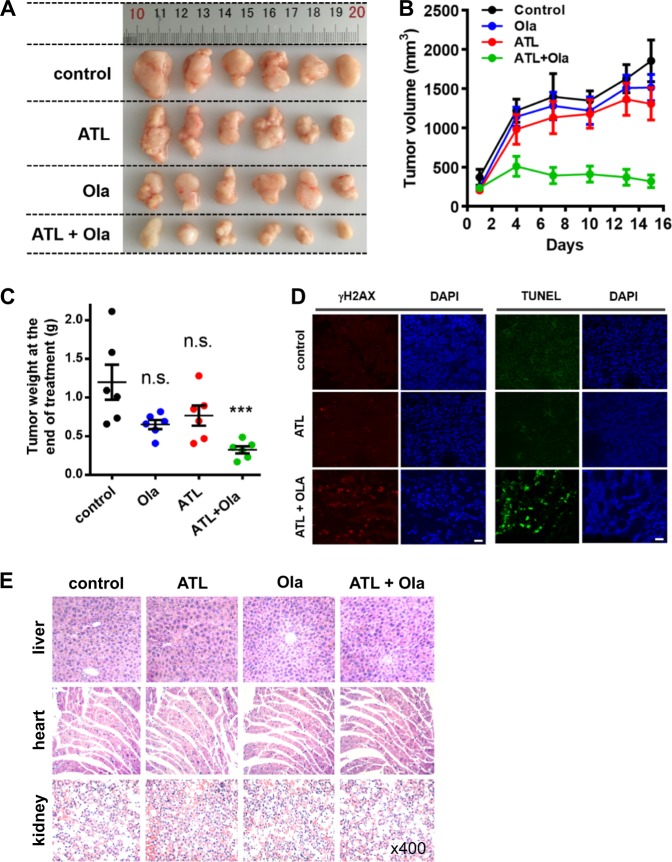
Coadministration of ATL and olaparib induces regression of tumor xenografts. PC-3 cells (2 × 10^6^) in 1:1 matrigel were inoculated subcutaneously into the left flanks of male athymic BALB/c nude mice. When the tumor volume reached approximately 150 mm^3^ (15 days after inoculation), mice were treated once daily with 50 mg/kg ATL oral gavage or 50 mg/kg olaparib intraperitoneal injection or both for 15 days. **a** Photograph of tumors dissected out from each mouse at the time of study termination. **b** Tumor volumes measured on the indicated days of treatment. Results were shown as mean ± SD. **c** Tumor weight measured at the end of the study. **d** Representative images of immunohistochemical staining of γH2AX and TUNEL in tumor tissues (scale bar: 20 μm). **e** Hematoxylin-eosin staining of liver, heart, and kidney tissue sections (magnification: ×400). *n.s.* not significant, ***p < 0.001 vs. vehicle control.

To assess the therapeutic effect of the ATL and olaparib combination in vivo, mice bearing PC-3 tumor xenografts were treated with either ATL (dosed once daily by oral gavage, 50 mg/kg), olaparib (dosed once daily by intraperitoneal injection, 50 mg/kg), or the combination of the 2 for 15 days. Treatment with either agent alone had no impact on tumor growth as compared to the vehicle control, however, the combination of ATL and olaparib completely inhibited the growth and caused substantial regression of the tumor (Fig. 7a–c). Immunohistochemical staining of the tumor tissues showed significantly increased γH2AX signals, which were accompanied by intense apoptosis revealed by TUNEL staining, only in the group treated by the combination of ATL and olaparib (Fig. 7d), suggesting that tumor regression likely resulted from DNA damage-induced apoptosis. No significant body weight loss was observed in the treated groups compared with the control group (data not shown), and microscopic examination of hematoxylin-eosin stained tissue sections of vital organs (liver, heart, and kidney) did not show any histological change that would indicate toxic effects of ATL (Fig. 7e), suggesting the combination of ATL and olaparib was well tolerated.

## Discussion

DNA damage and defective DNA repair promote mutations and tumorigenesis but also render cancer cells vulnerable to additional DNA damage or disruption of compensatory DNA repair pathways [1, 2, 56]. Exploiting such vulnerabilities to target cancer is emerging as a promising and highly selective anticancer strategy. Cancer cells with deficient HR are exquisitely sensitive to inhibition of PARP [12, 13] and several PARPis are now approved for the treatment of BRCA1/2-mutated ovarian and breast cancers, with dozens more in various stages of preclinical and clinical development [15, 16]. However, most ovarian and breast cancers, and nearly all other types of cancer, have normal BRCA1 and BRCA2; and even in BRCA1/2-mutant tumors, responses to PARPi are heterogeneous and initially responsive cancers eventually develop PARPi resistance. Thus, developing strategies to broaden the therapeutic potential of PARPis and to overcome PARPi resistance is of great importance.

Studies in the past have shown that inhibition of PARP can sensitize cancer cells to DNA damaging cytotoxic agents irrespective of HR status [17, 18], however, clinical use of PARPis in combination with DNA damaging chemotherapy is limited due to normal tissue toxicity [16]. ROS are a major class of DNA-damaging agents and play very important roles in suppressing cancer initiation and progression [25, 26, 57–60]. Owing to dysregulated proliferation, cancer cells exhibit abnormal metabolism and high levels of intrinsic oxidative pressure, leading to their dependence on antioxidant systems and DNA repair for survival [26, 59–62]. The thioredoxin antioxidant pathway is upregulated in tumors and simultaneous inhibition of the thioredoxin and glutathione antioxidant pathways causes synergistic cancer cell death [26]. These features render cancer cells more sensitive to exogenous oxidative insult than normal cells, thus providing a unique opportunity to induce DNA damage selectively in cancer cells by targeting antioxidant systems or promoting ROS generation [27, 41].

ATL is a natural sesquiterpene lactone that shares a α-methylene-γ-lactone moiety with known TrxR inhibitors [34]. It binds to TrxR and irreversibly inhibits the enzymatic activity of both purified and cellular TrxR, resulting in marked elevation of oxidized thioredoxin and ROS in cancer cells. Overexpression of functional TrxR attenuated ATL-induced accumulation of ROS and cancer cell toxicity [34]. Here we showed that a noncytotoxic dose of ATL induced a rapid increase in ROS levels specifically in cancer cells. The ATL-induced, cancer-specific ROS increase was followed by NAC-suppressible accumulation of 8-oxoG, DNA strand breaks and PAR, indicating generation of oxidative DNA damage and activation of PARP1/2. These results demonstrated the potential of using nontoxic doses of ATL to produce oxidative DNA damage selectively in cancer cells. Currently, pharmacokinetics (PK) data of ATL in humans are not available, however, studies in rat reported a plasma *C*_max_ of 1.103 mg/L (4.75 μM) after intravenous administration of Radix Inulae extract containing 3.43 mg/kg ATL [63] and 0.03 mg/L (0.12 μM) after oral administration at a dose of 50 mg/kg ATL [64]. Our results showed that a significant increase in ROS levels could be induced in cancer cells by an ATL concentration as low as 0.625 μM and strong synthetic lethality was achieved by 1 μM ATL combined with olaparib. Thus, it appears that an effective ATL concentration may be achievable in vivo at least through intravenous administration, and ATL could serve as a chemical scaffold to be further developed.

Remarkably, adding a nontoxic dose of the PARPi olaparib to nontoxic ATL resulted in potent synergistic cytotoxicity specifically in cancer cells. The toxicity was directly related to ATL-induced oxidative damage as it was significantly reduced by NAC. OGG1 depletion or inhibition significantly reduced the synergistic cytotoxicity, revealing that the lethality resulted primarily from the repair of oxidized DNA bases. The non-trapping PARPi veliparib did not synergize with ATL to cause cytotoxicity and depletion of PARP1 did not sensitize cells to nontoxic ATL, on the contrary, the synergistic toxicity between ATL and olaparib was abolished in the absence of PARP1, demonstrating that the lethality associated with the ATL and olaparib combination was dependent on PARP-trapping.

Trapped PARP-DNA complexes, together with unrepaired SSBs, collide with ongoing DNA replication forks to result in fork stalling and replication stress [3, 10]. In the cancer cells treated by the combination of ATL and olaparib, the markedly increased levels of γH2AX and chromatin-bound RPA indicated intense replication stress [50, 53], and the CIdU and IdU labeling experiment revealed severe fork stalling. Rapid generation of an excessive number of stalled replication forks, together with impaired fork reversal and restart associated with PARP inhibition, promote fork collapse and generation of lethal DSBs [3, 4]. The results of the comet assay and 53BP1 staining confirmed the presence of extensive DSBs. In addition, generation of ssDNA due to fork stalling may exhaust the RPA pool to result in replication catastrophe [53].

Oxidative DNA damage generated in some special situations has been shown to synergistically induce cancer cell lethality with PARPis [31–33, 65, 66]. Here, we show that combining the PARPi olaparib with the highly tumor-specific DNA damaging agent, ATL, results in synergistic lethality at nontoxic doses of both drugs. The combination exploits a cancer vulnerability driven by the high levels of intrinsic oxidative pressure in cancer cells. Normal tissues are spared due to their lower basal ROS output. As high oxidative pressure is a universal feature of tumors, both primary and relapsed, our findings may open new routes to broaden the therapeutic potential of PARP inhibitors.

## Materials and methods

### Cell line and cell culture

The SW480, A549, PC-3, and 293T cell lines were purchased from the American Type Culture Collection (ATCC, Manassas, VA, USA), BEAS-2B, NCM460 and all other cell lines were obtained from the Cell Bank of the Chinese Academy of Sciences (Shanghai, China). All cell lines were authenticated by STR profiling, routinely tested for mycoplasma, and maintained at 37 °C in a humidified incubator in 5% CO_2_.

### Materials

Olaparib (AZD2281, S1060), veliparib (ABT-888, S-1004), Z-VAD-FMK (S7023), and cisplatin (S1166) were purchased from Selleck (Houston, TX, USA). The OGG1 inhibitor O8 (SML1697), doxycycline (D1822), 5-chloro-2′-deoxyuridine (CIdU) (C6891), PEG 300 (90878) and *N*-acetyl-l-cysteine (NAC) (BP907) were purchased from Sigma-Aldrich (St. Louis, MO, USA). Alantolactone (B21267) was bought from Yuanye (Shanghai, China), idoxuridine (IdU) (HY-B0307) from MCE (Monmouth Junction, NJ, USA), auranofin (B7678) from APExBIO (Houston, TX, USA) and aphidicolin (ab142400) from Abcam (Cambridge, UK). Stock solutions of ATL and olaparib were made in 100% dimethyl sulfoxide (DMSO) (Sigma-Aldrich) and working solutions were prepared in complete cell culture medium. The solution with the same concentration of DMSO but without the test compound was used as vehicle control. Primary antibodies include γH2AX-pS139 polyclonal antibody (ab11174), poly(ADP-ribose) polymer (ab14459), and BrdU (ab6326, ab8152) (Abcam); cleaved caspase-3 (9664S), H3-pS10 (9706L), CHK1-pS317 (12302S), and CHK2-pT68 (2661S) (Cell Signaling, Danvers, MA, USA); RPA32 (bs-4182R), CHK1 (bs1681R), OGG1 (bs3687R), GAPDH (bs10900R), and β-actin (bsm33036M) (Bioss, Beijing, China); H3 (abs131869), H2AX (abs131731), CHK2 (abs131635) (Absin Bioscience, Shanghai, China); RPA32-pS4/8 (NBP1-23017) (NOVUS, Centennial, CO, USA), 53BP1 (A300-272A) (Bethyl, Montgomery, TX, USA), γH2AX-pS139 monoclonal antibody (14-9865-82) (ThermoFisher, Waltham, MA, USA). Secondary antibodies include goat anti-mouse-Alexa 488, goat anti-rabbit-Cy3, goat anti-rat-Alexa-488, goat anti-mouse-horseradish peroxidase and goat anti-rabbit-horseradish peroxidase (Jackson ImmunoResearch, West Grove, PA, USA).

### Cell viability assay

Cells were seeded in 96-well plates and at the end of drug treatment, 20 μl of 5 mg/ml 3-(4,5-dimethylthiazol-2-yl)-2,5-diphenyl tetrazolium bromide (MTT) (Sigma-Aldrich) was added to each well. The plates were incubated for 4 h at 37 °C and 100 μl of DMSO was added to each well. The plate was left on a plate shaker for 30 min with gentle shaking at room temperature. The absorbance of each well was measured at 595 nm.

### Colony formation assay

Cells were seeded into 12-well plates at 500 cells/well. Starting from the second day, cells were treated with drug for 7 days and then stained with crystal violet, dissolved in 70% ethanol and absorbance at 595 nm was obtained.

### Immunofluorescence microscopy

For detection of chromatin-bound RPA foci, cells were incubated in pre-extraction buffer (100 mM NaCl, 300 mM sucrose, 3 mM MgCl_2_, 1 mM EGTA, 0.2% Triton X-100 and 10 mM PIPES, pH 6.8) for 5 min; for Rad51 staining, cell monolayers were irradiated with 3 Gy X-rays delivered by the X-RAD 320ix biological irradiator or treated by 0.1 μM cisplatin for 24 h, followed by treatment with vehicle control, 10 μΜ ATL or 10 μM ATL combined with 10 μM olaparib for the indicated lengths of time; to label DNA replicating cells, cultures were incubated in 10 µM EdU for 20 min and processed with the Click-iT EdU Alexa Fluor 488 imaging kit (C0071S) (Beyotime Biotechnology, Shanhai, China). After the above treatments, cells were fixed in 4% paraformaldehyde for 20 min and then in ice-cold methanol:acetone for 20 min Subsequently, cells were blocked in PBST (0.05% Tween-20 in PBS) with 2% BSA for 1 h, followed by incubation in primary and secondary antibodies. For immunostaining of 8-oxoG, fixed cells were incubated in Cy3-conjugated avidin [67] (Rockland Immunochemicals, Limerick, PA, USA) (0.5 μg/ml) for 1 h at room temperature. TUNEL staining was performed using the one-step TUNEL kit (Beyotime Biotechnology) according to instructions provided by the manufacturer.

### Flow cytometry analysis of cell cycle and apoptosis

For cell cycle analysis, cells were fixed in 70% ethanol for 1 h at −20 °C, treated with 100 μg/ml RNase A (Sigma-Aldrich) for 30 min at 37 °C and stained with propidium iodide (100 µg/ml in 1% sodium citrate) for 15 min in the dark. Cell cycle profiles were analyzed by the MoFlo XDP Cell Sorter (Beckman Coulter, Indianapolis, IN, USA) with the FlowJo software (FlowJo, Ashland, OR, USA). For analysis of apoptosis, cells were stained with Annexin V-FITC and propidium iodide (PI) (Bestbio, Shanghai, Chain) at room temperature in the dark for 20 min, analyzed by the MoFlo XDP Cell Sorter using the CytExpert software (Beckman Coulter).

### DR-GFP HR repair assay

To establish stable cell lines harboring the DR-GFP gene cassette, the pDR-GFP plasmid (Addgene, #26475) was transfected into PC-3 or A549 cells to get puromycin-resistant clones. Next, 5 μg of pCBASce1 (Addgene, #26477) and 1.5 μg of pDsRed-N1 (Clontech, Mountain View, CA, USA) were co-transfected into the stable cell lines, and cells were treated with drugs or vehicle control for 12 h.

### Measurement of cellular ROS

Intracellular ROS levels were measured by flow cytometry using a cell-based ROS assay kit (S0033) (Beyotime Biotechnology). Cells grown in six-well plates were incubated with 10 μM dichlorofluorescein diacetate (DCFH-DA) for 30 min at 37 °C and analyzed by the MpFlo XDP Cell Sorter.

### Lentiviral shRNA knockdown

PARP1 or OGG1 knockdown was achieved via transfection of cells with doxycycline inducible specific shRNA lentiviruses. The human PARP1 and OGG1-specific shRNA sequences were synthesized based on information validated by Sigma-Aldrich (PARP1: TRCN0000007929, TRCN0000007932; OGG1: TRCN0000314672, TRCN0000314739). These sequences were inserted into the pTet-pLKO-puro plasmid (Addgene, #21915) and lentiviral particles were produced in 293T cells transfected with the Tet-pLKO1-puro vector and the packaging vectors pMD2.G (Addgene, #12259) and psPAX2 (Addgene, #12260).

### Detection of chromatin-bound proteins

After washing with PBS, cells were resuspended in 200 µl of solution A (10 mM HEPES at pH 7.9, 10 mM KCl, 1.5 mM MgCl_2_, 0.34 M sucrose, 10% glycerol, 1 mM DTT, 10 mM NaF, 1 mM Na_2_VO_3_ and protease inhibitors). After adding Triton X-100 to a final concentration of 0.1%, the cells were left on ice for 5 min and then centrifugated at 1400 × *g* for 4 min to separate the cytoplasm from nuclei. The nuclei fraction was thoroughly washed with solution A and resuspended in 200 µl of solution B (3 mM EDTA, 0.2 mM EGTA, 1 mM DTT and protease inhibitors). After incubation at 4 °C for 30 min, chromatin was separated from soluble nuclear substances by centrifugation at 1700 × *g* for 4 min After washing three times with solution B, the chromatin fraction was collected by centrifugation at 1700 × *g* for 4 min, resuspended in 200 µl of PBS and sheared by sonication. Protein binding in the chromatin fraction was assessed by Western blot.

### Comet assay

Five hundred cells were added to 1% low melting point agarose maintained 37 °C, laid on frosted slides (ThermoFisher) and froze at 4 °C for 20 min in the dark, followed by incubation in precooled lysis buffer (2.5 M NaCl, 100 mM EDTA, 10 mM Tris-HCl and 1% sodium laurylsarcosine, pH 7.5 for neutral comet assay; pH 10.0 for alkaline comet assay) overnight. Triton X-100 was added to a final concentration of 1% before cooling. Slides were equilibrated for 20 min in precooled running buffer (90 mM Tris-HCl, 90 mM boric acid, 2 mM EDTA, pH 7.5 for neutral comet assay; 300 mM NaOH, 1 mM EDTA, pH > 13 for alkaline comet assay) and electrophoresis was run at 20 V for 30 min The slides were washed in neutralizing buffer (0.4 M TRIS, pH 7.5), placed in cold 70% ethanol for 5 min, dried and stained with Vista Green DNA dye. The tail moment was defined as percentage of tail DNA × tail length, quantified using the TriTek CometScore sofware (TriTek Corp., Sumerduck, VA, USA).

### Pulse-labeling of DNA replication by CIdU and IdU

Cells were labeled with 250 μM CIdU for 30 min, incubated in fresh medium with or without drug for 3 h, followed by incubation in fresh medium containing 25 μM IdU for 30 min The cells were fixed in methanol:acetone (3:1) for 15 min, followed by blocking with 3% BSA containing 0.03% Triton-X 100 for 30 min and incubation with primary and secondary antibodies.

### Tumor xenograft experiments

All mouse studies followed the protocols approved by the Institutional Animal Care and Use Committee of Jilin University. PC-3 cell suspensions were prepared in 1:1 matrigel (CORNING, Corning, NY, USA) and 2 × 10^6^ cells were inoculated subcutaneously into the left flanks of male athymic BALB/c nude mice (6–8 weeks old) (Charles River, Boston, MA, USA). Tumors were measured with calipers and the tumor volume was calculated according to the formula *V* = 1/2 length × width^2^. When the tumor volume reached approximately 150 mm^3^ (15 days after inoculation), mice were randomized into treatment and control groups (*n* = 6 each group) (no statistical methods were used to pre-determine sample size). The mice were treated once daily with 50 mg/kg ATL (1% DMSO + 40% PEG 300) oral gavage (p.o.) or 50 mg/kg olaparib (4% DMSO + 30% PEG 300) intraperitoneal injection (i.p.) or combination of both for 15 days. Tumor volume and body weight were measured every three days, and tumor weight was measured at the end of the study. The investigators performing tumor measurements were blinded to the experimental design and the identity of treatment.

### Histology

Heart, liver, and kidney tissues were fixed in 10% formalin, processed and embedded in paraffin. After deparaffinization and rehydration, 5 μm-thick sections were stained with hematoxylin solution for 5 min, followed by five dips in 1% acid ethanol (1% HCl in 70% ethanol) and then rinsed in distilled water. The sections were then stained with eosin solution for 3 min.

### Statistical analysis

All data were presented as mean ± SD of three independent experiments. Statistical comparisons were performed with the GraphPad Prism 7 software using the two-tailed Student’s *t*-test. *p* < 0.05 was considered to indicate a statistically significant difference.

## Supplementary information

Supplementary figure legends

Supplementary figure 1

Supplementary figure 2

Supplementary figure 3

Supplementary figure 4

Supplementary figure 5

Supplementary figure 6

Supplementary figure 7

Supplementary figure 8
