# Supplementary material for: Synergistic lethality between PARP-trapping and alantolactone-induced oxidative DNA damage in homologous recombination-proficient cancer cells
Source: Oncogene. 2020 Feb 6;39(14):2905–20. doi: 10.1038/s41388-020-1191-x (PMC7118026; doi:10.1038/s41388-020-1191-x)
Supplement: Supplementary file 1 — Supplementary figure legends [file 41388_2020_1191_MOESM1_ESM.docx]

**Supplementary Fig. 1** Induction of ROS elevation in cancer cells by a nontoxic dose of ATL. **A** Colony formation assay. PC-3, SW480, A549, HCC827, Caco-2, SW1116, HCT116, T47D, M21 and PANC-1 cancer cells were treated by 10 μM ATL for 7 days, stained by crystal violet and then dissolved in 70% ethanol. Absorbance at 595 nm was measured using a microplate reader. Data were presented as mean ± SD of three independent experiments. 10 μM ATL had no significant impact on the clonogenic growth of the cancer cells. **B** Measurement of ROS by flow cytometry. Treatment by 10 μM ATL for 30 min resulted in a significant increase in ROS levels in SW480 and A549 cancer cells. NAC blocked the ROS increase in these cancer cells. **C** Flow cytometry measurement of ROS in additional cancer and noncancerous cell lines. A significant increase in ROS levels was induced within 30 min of treatment by 10 μM ATL in all the cancer but not the BEAS-2B and HEK293 noncancerous cell lines. **D** Dose-dependency of ATL-induced ROS increase. Treatment by sublethal concentrations of ATL for 30 min dose-dependently increased ROS levels in PC-3 and A549 cancer cells, and a dose of ATL as low as 0.625 μM induced a significant increase in ROS levels. n.s.: not significant, *: *p* < 0.05, **: *p* < 0.01, ***: *p* < 0.001, ****: *p* < 0.0001 vs vehicle control.

**Supplementary Fig. 2** Induction of oxidative DNA damage and PARylation in SW480 and A549 cancer cells by a nontoxic dose of ATL. **A** Immunofluorescent staining of cellular 8-oxoG by Cy3-conjugated avidin in A549, SW480 and NCM460 cells treated by 10 μM ATL for 12 h in the presence or absence of 10 mM NAC. Nuclear intensity was measured using the ImageJ software and the data were processed by the Prism software. **B** Detection of DNA strand breaks in A549 and SW480 cells by alkaline comet assay. Treatment by 10 μM ATL for 12 h resulted in a prominent increase in the number of DNA strand breaks (DSBs and SSBs) which was blocked by 10 mM NAC. **C** Western blot analysis of PAR in A549, SW480 and NCM460 cells. 10 μM ATL resulted in a time-dependent increase in PAR levels in the A549 and SW480 cancer but not the NCM460 noncancerous cells. NAC blocked the PAR increase in the cancer cells. n.s.: not significant, **: *p* < 0.01, ***: *p* < 0.001 vs vehicle control.

**Supplementary Fig. 3** ATL synergizes with olaparib to induce cancer cell lethality. **A** MTT assay. PC-3, SW480 and A549 cancer cells were treated by olaparib for 72 h, the IC_50_ values were derived by the Prism software. **B** Colony formation assay. SW480, A549 and BEAS-2B cells were treated by 10 μM ATL, 10 μM olaparib (Ola), or combination of 10 μM ATL and 10 μM Ola or 10 μM ATL and 10 μM veliparib (Vel) for 7 days. Cells were stained by crystal violet and dissolved in 70% ethanol, absorbance at 595 nm was measured using a microplate reader. Data were presented as mean ± SD of three independent experiments. The combination of 10 μM ATL and 10 μM Ola, but not 10 μM ATL and 10 μM Vel, completely inhibited the clonogenic growth of SW480 and A549 but not the BEAS-2B cells and 10 mM NAC blocked the inhibition. **C** MTT proliferation assay. SW480 and A549 cells were treated by 1, 2, 4, 8, or 16 μM olaparib alone or combined with 10 μM ATL for 72 h. Olaparib in combination with 10 μM ATL dose-dependently inhibited the growth of SW480 and A549 cells, while olaparib alone had no impact. **D** Determination of combination index (CI) values. SW480 and A549 cells were treated by 10 μM ATL and the indicated concentrations of olaparib for 72 h. The CI values were determined by the Chou-Talalay method using the CompuSyn software. n.s.: not significant, **: *p* < 0.01, ***: *p* < 0.001 vs vehicle control.

**Supplementary Fig. 4** Untreated and treated PC-3, SW480 and A549 cells have normal HR. **A** RAD51 foci formation after ionizing radiation (IR) exposure. SW480 and A549 cells were irradiated with 3 Gy X-rays, treated with vehicle control, 10 μM ATL alone or in combination with 10 μM olaparib (Ola), and immunostained at the indicated time points. Upper panel: representative micrographs of SW480 and A549 cells stained with anti-RAD51 and counterstained with DAPI 2 h after IR (scale bar: 10 μm). Lower panel: quantification of the percentage of cells with more than 10 RAD51 foci at the indicated times after IR. **B** Induction of RAD51 foci by cisplatin. A549 cells were treated with 0.1 μM cisplatin for 24 h, followed by treatment with vehicle control, 10 μM ATL alone or in combination with 10 μM olaparib (Ola) for the indicated lengths of time. **C** DR-GFP reporter assay. PC-3 and A549 cells harboring the DR-GFP reporter were transiently transfected with plasmids expressing I-SceI and DsRed for 24 h and then treated with vehicle control or combination of 10 μM ATL and 10 μM olaparib (Ola) for 12 h. Left: representative images of red cells (expressing I-SceI and RFP) and green cells (expressing GFP recovered through HRR) (scale bar: 25 μm). Right: flow cytometry analysis of red and green A549 cells. **D** Synergy between ATL and olaparib in BRCA-deficient cancer cells. The cytotoxicity of olaparib in the BRCA1-deficient MDA-MB-436 breast cancer cells was significantly enhanced by both 2 μM and 4 μM ATL. n.s.: not significant, *: *p* < 0.05, ***: *p* < 0.001 vs vehicle control.

**Supplementary Fig. 5** Synergy between olaparib and ATL results from PARP-trapping. **A** Western blot verification of shRNA-mediated knockdown of OGG1 and PARP1 in SW480 and A549 cells. **B** MTT proliferation assay. Wild-type, OGG1-depleted or inhibited by 8 μM O8, and PARP1-depleted SW480 and A549 cells were treated by 2, 4, 8, or 16 μM olaparib in combination with 10 μM ATL for 72 h. **C** MTT assay. Wild-type or PARP1 depleted SW480 and A549 cells were treated by 10 μM ATL or the combination of 10 μM ATL and 10 μM olaparib (Ola) for 72 h. PARP1 knockdown did not sensitize SW480 and A549 cells to 10 μM ATL. **D** Detection of chromatin-bound PARP1 by Western blot. A549 cells were treated by 10 μM ATL, 10 μM olaparib (Ola), 10 μM veliparib (Vel) or the combination of 10 μM ATL with 10 μM olaparib or veliparib for 24 h. n.s.: not significant, ***: *p* < 0.001 vs vehicle control.

**Supplementary Fig. 6** ATL synergizes with olaparib to induce intense replication stress in cancer cells. **A** Immunofluorescent staining of γH2AX and EdU. A549 cells were treated by 10 μM ATL, 10 μM olaparib (Ola) or the combination of the two, with or without 10 mM NAC or 5 μM aphidicolin (APC), for 12 h. At the end of drug treatment, cells were pulse-labeled with 10 µM EdU for 20 min (scale bar: 20 μm). γH2AX and EdU positive cells were measured using the ImageJ software and the data were processed by the Prism software. **B** Western blot analysis. A549 cells were treated by 10 μM ATL, 10 μM olaparib (Ola) or the combination of the two for the indicated times. **C** Immunofluorescent staining of RPA32 foci. SW480 and A549 cells were treated by 10 μM ATL, 10 μM olaparib (Ola) or the combination of the two for 12 h (scale bar: 10 μm). Nuclear RPA32 intensity was measured using the ImageJ software and the data were processed by the Prism software. *: *p* < 0.05, **: *p* < 0.01, ***: *p* < 0.001, ****: *p* < 0.0001 vs vehicle control.

**Supplementary Fig. 7** ATL synergizes with olaparib to induce DSB in cancer cells. **A** Neutral and alkaline comet assay. SW480 and A549 cells were treated by vehicle control or the combination of 10 μM ATL and 10 μM olaparib (Ola) for 12 h. **B** Immunofluorescent staining of 53BP1. SW480 and A549 cells were treated by 10 μM ATL, 10 μM olaparib (Ola) or the combination of the two, with or without 10 mM NAC or 5 μM aphidicolin (APC), for 12 h. 53BP1 positive cells were measured using the ImageJ software and the data were processed by the Prism software. ***: *p* < 0.001, ****: *p* < 0.0001 vs vehicle control.

**Supplementary Fig. 8** Induction of cell cycle arrest and apoptosis by ATL and olaparib. **A** Flow cytometry analysis of cell cycle distribution. Treatment by the combination of 10 μM ATL and 10 μM olaparib (Ola) caused progressive accumulation of SW480 and A549 cells in the S and G_2_/M phases within the first 48 h, and then the G_2_/M but not the S population started to fall, meanwhile, the sub-G_1_ population increased steadily. **B** Western blot analysis of phosphorylated H3. The levels of H3-pS10 in A549 cells decreased sharply after treatment by the combination of 10 μM ATL and 10 μM olaparib (Ola) for 12 h and longer, indicating that the cells in the G_2_/M population were in early-to-mid G_2_ phase. **C** Western blot analysis of cleaved caspase 3. Treatment by the combination of 10 μM ATL and 10 μM olaparib (Ola) caused a rapidly progressive increase in the levels of cleaved caspase 3 in the A549 cells. **D** Flow cytometry analysis of apoptosis in SW480 and A549 cells. Treatment by the combination of 10 μM ATL and 10 μM olaparib (Ola) caused a time-dependent increase in Annexin V-positive cells, most of which were propidium iodide (PI) negative, indicating they were in early apoptosis. The pan-caspase inhibitor Z-VAD-FMK suppressed the increase in Annexin V-positive cells.
