# Supplementary figures and images for: Synergistic lethality between PARP-trapping and alantolactone-induced oxidative DNA damage in homologous recombination-proficient cancer cells
Source: Oncogene. 2020 Feb 6;39(14):2905–20. doi: 10.1038/s41388-020-1191-x (PMC7118026; doi:10.1038/s41388-020-1191-x)

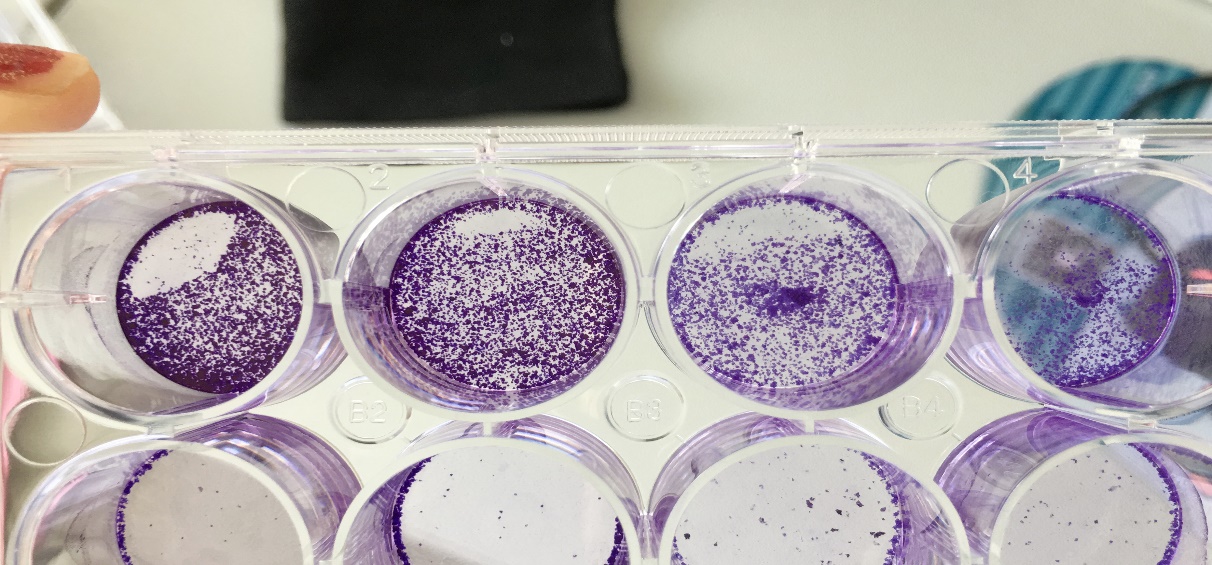

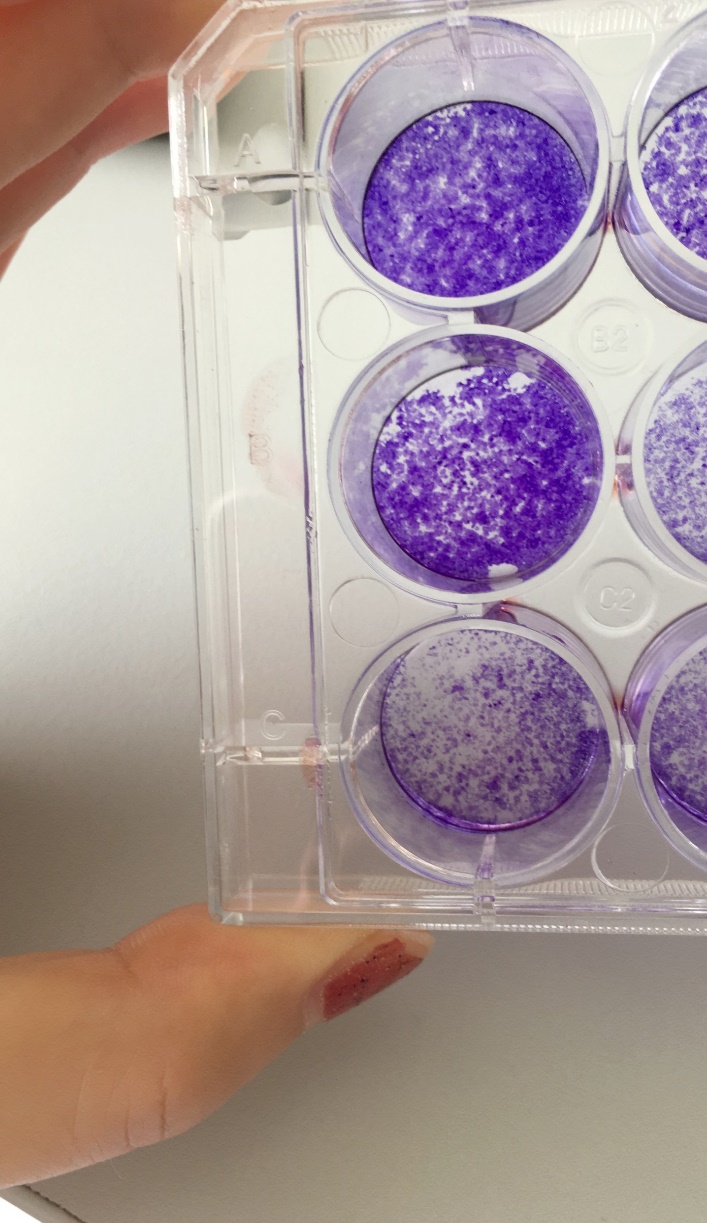

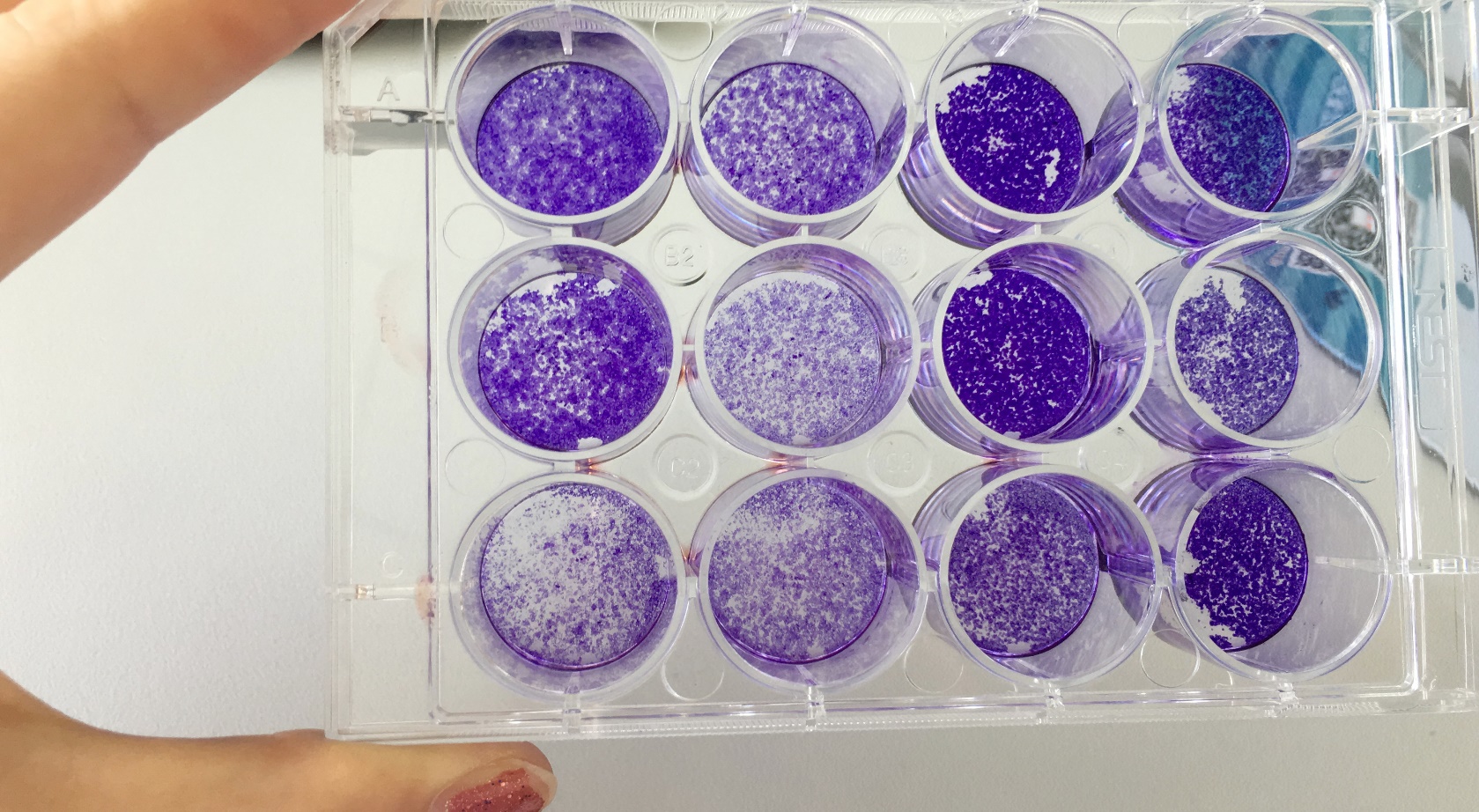

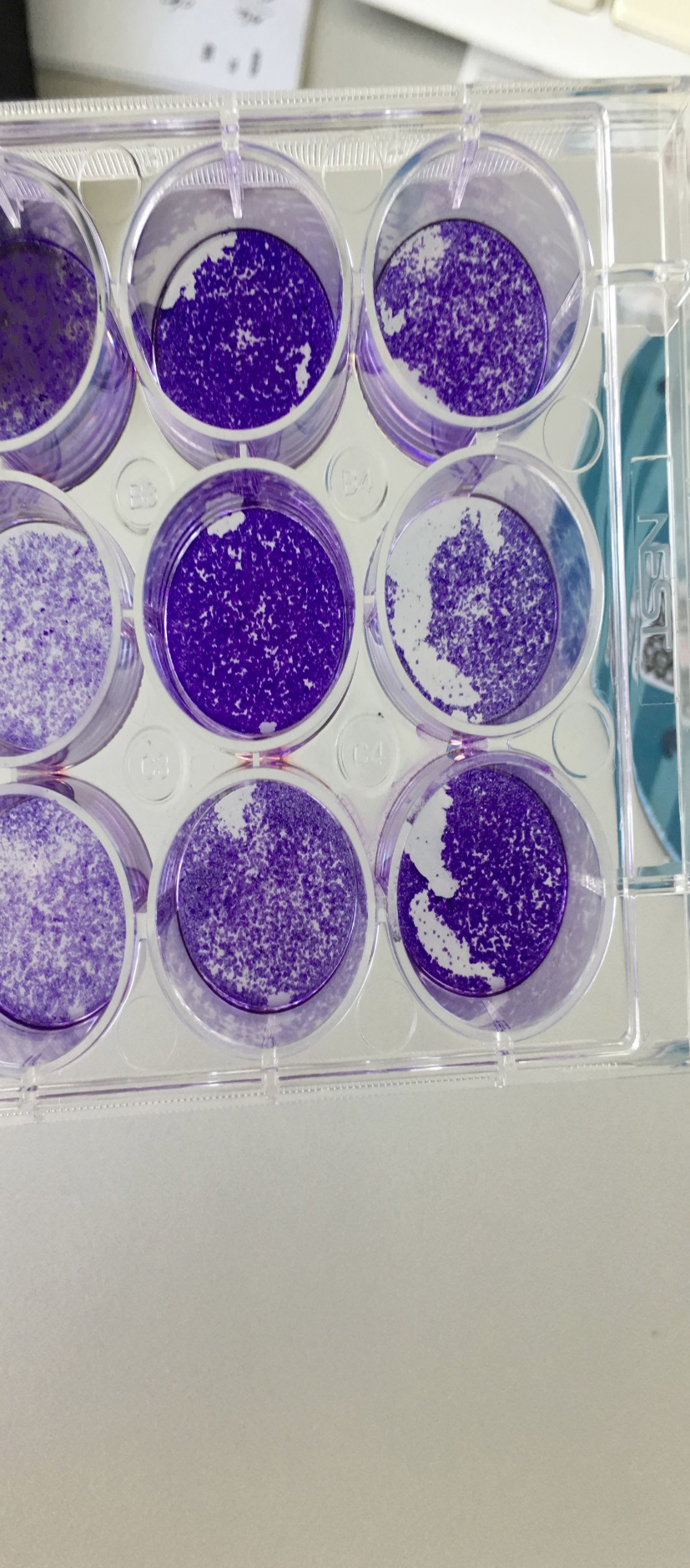

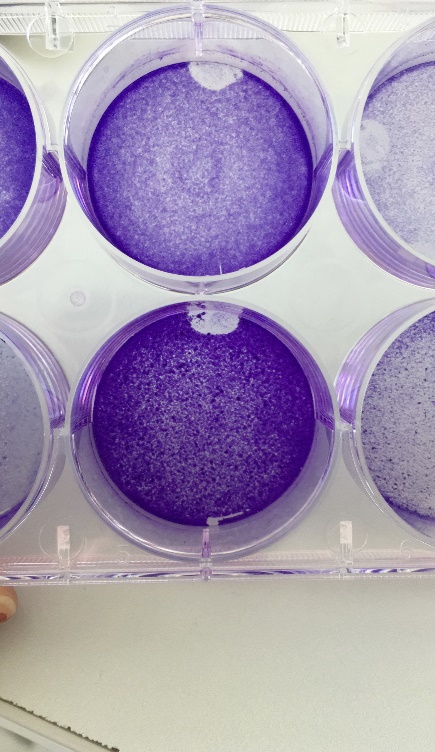


**B**


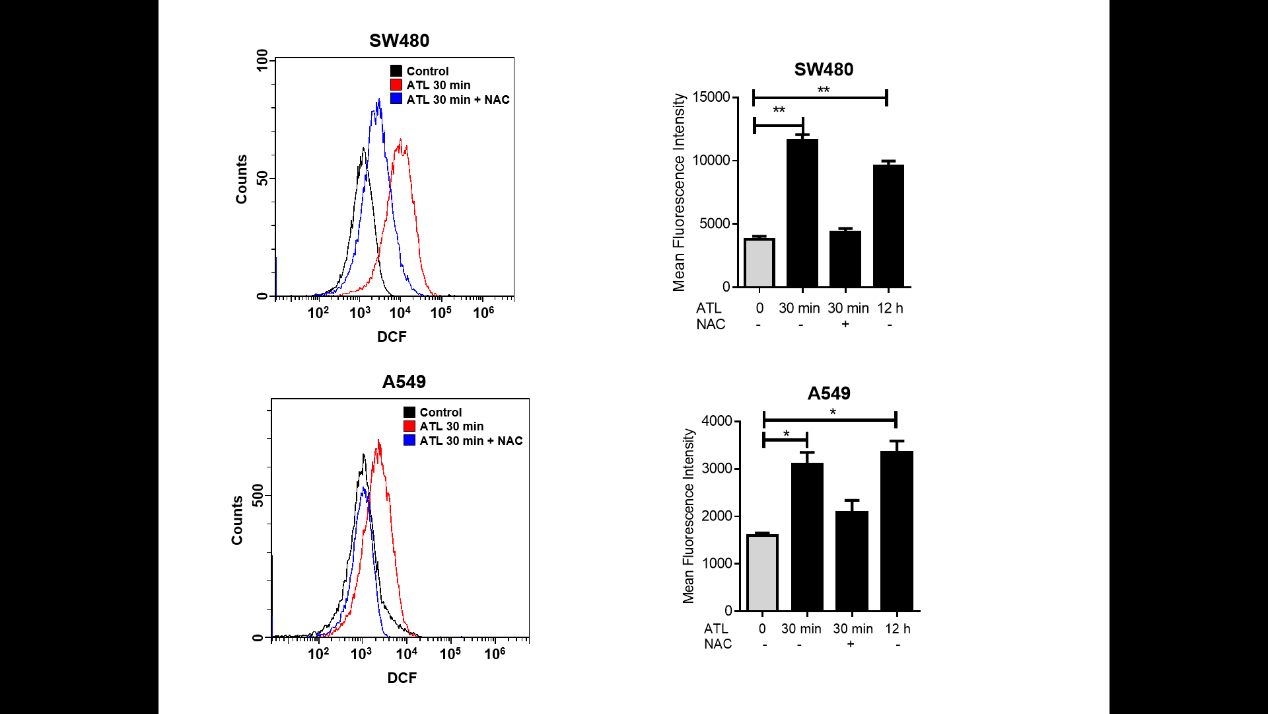

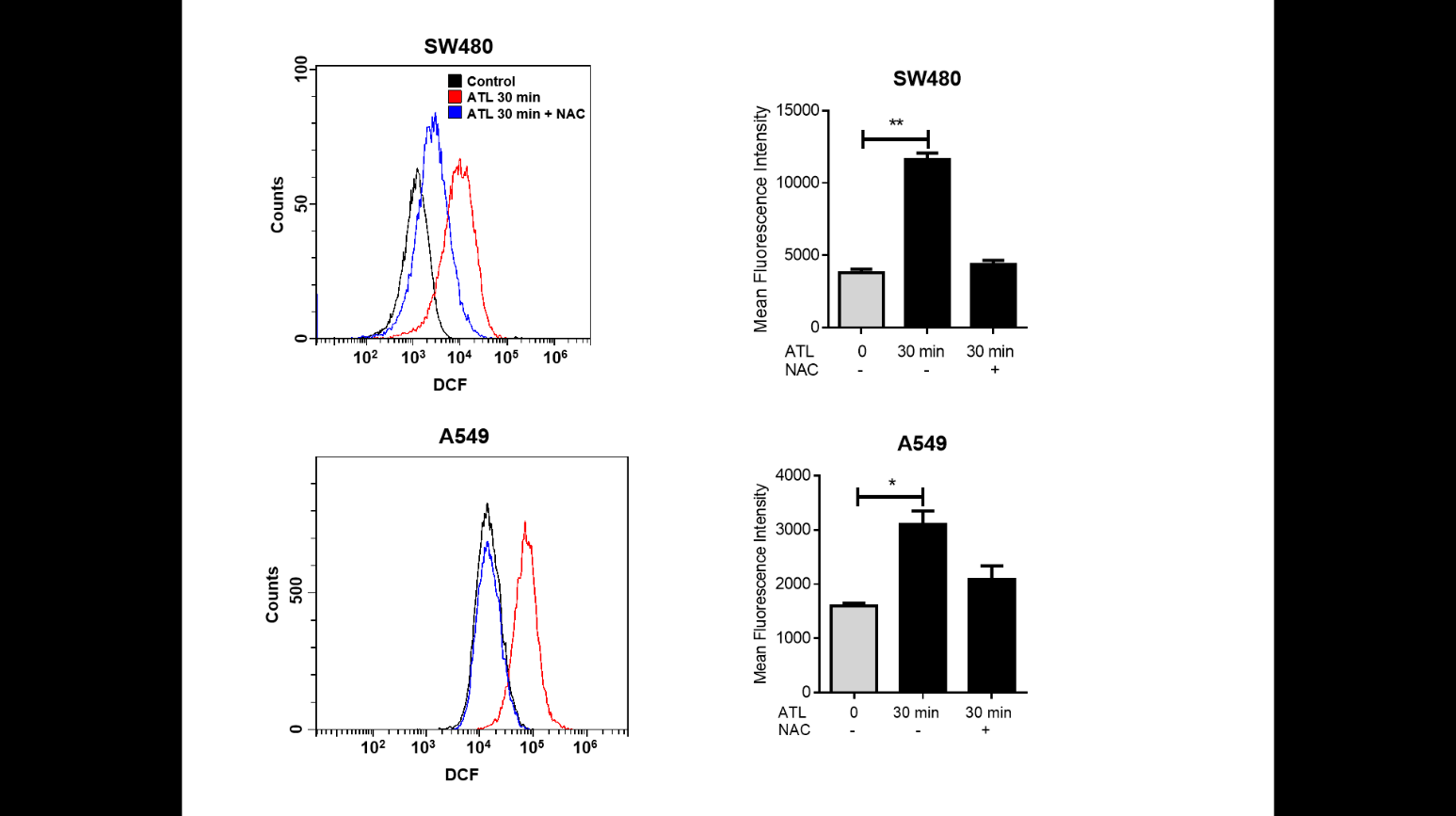

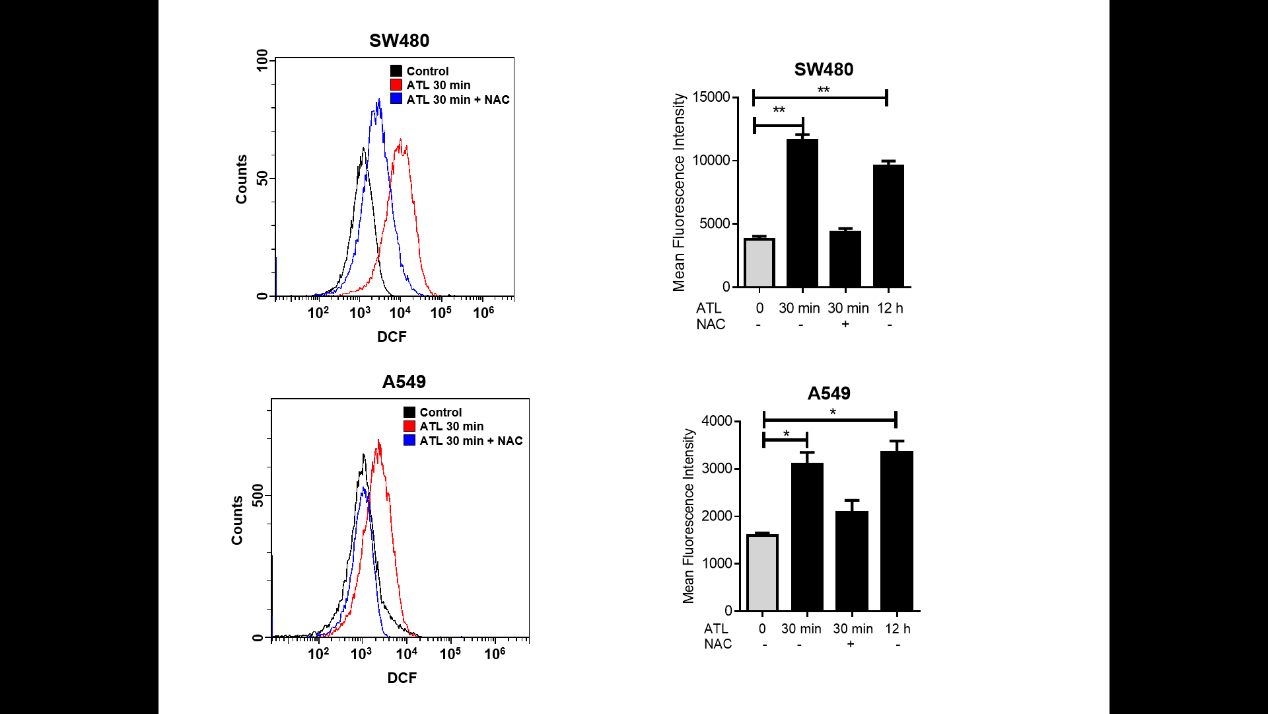


**D**


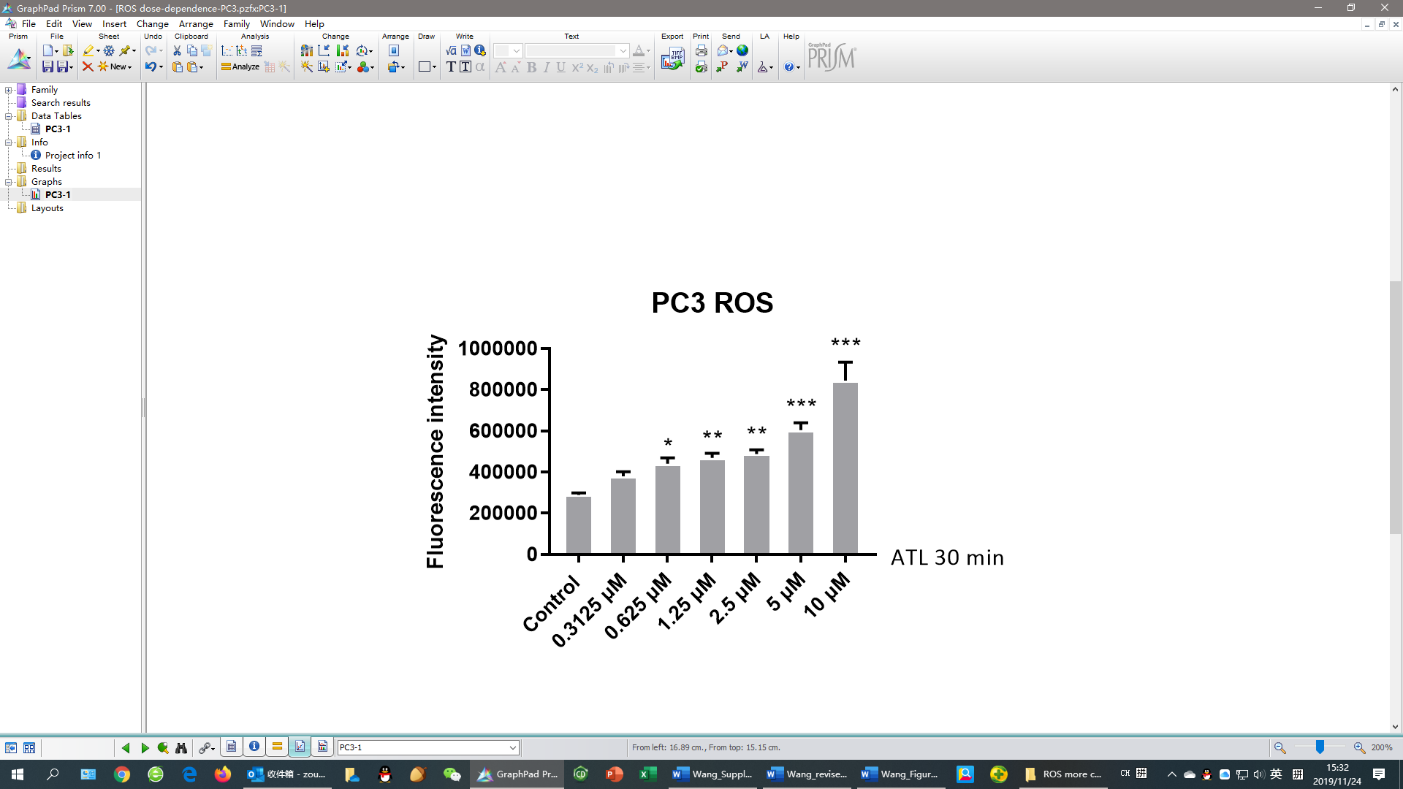


**PC-3**


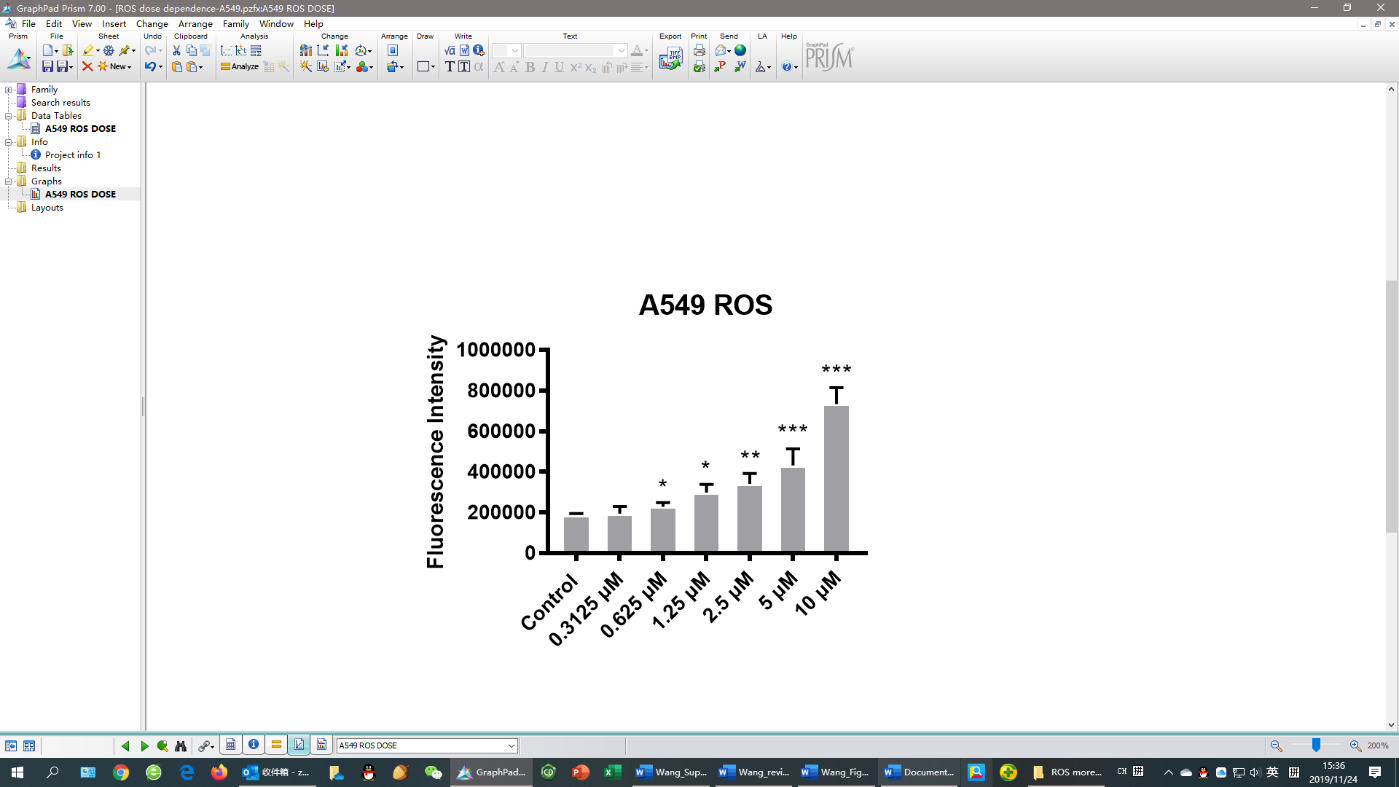


**SW480**

**A**

**Control**

**ATL**


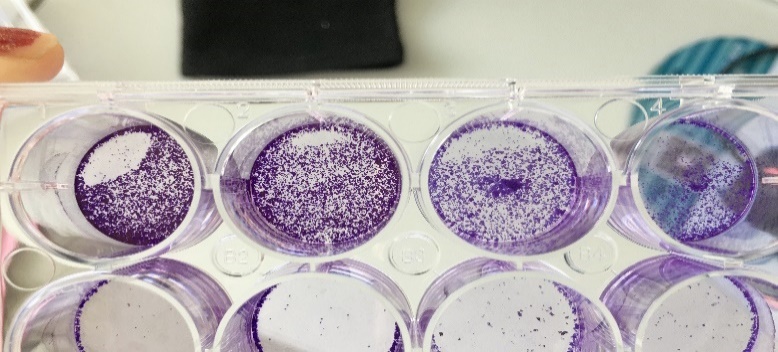

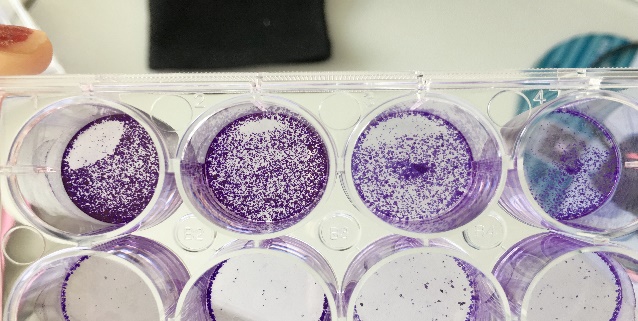

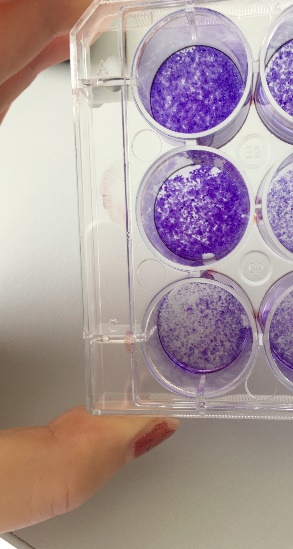

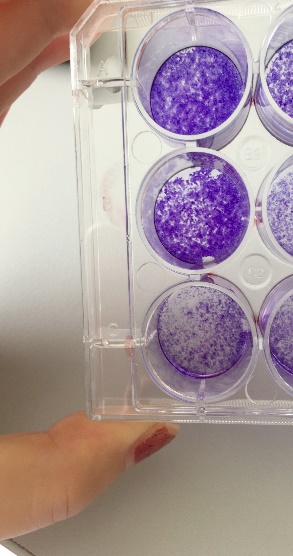

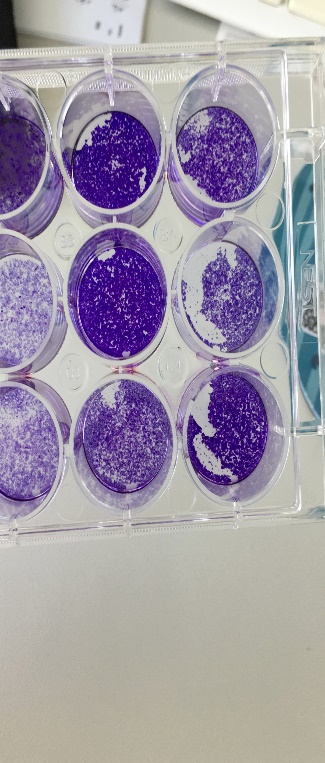

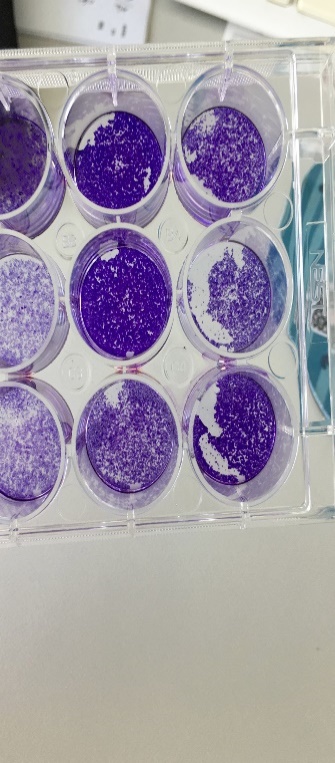

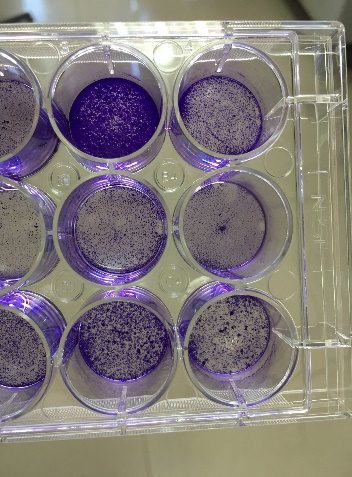

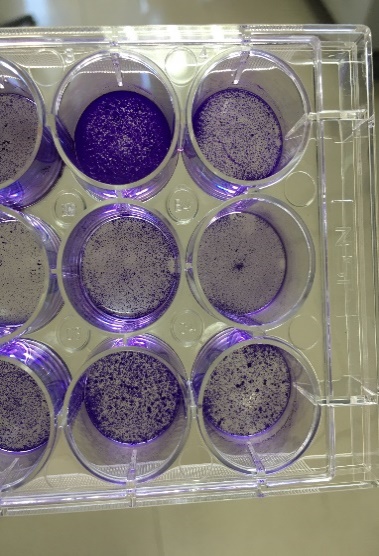

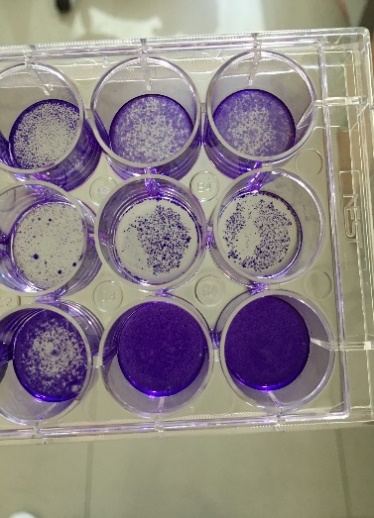

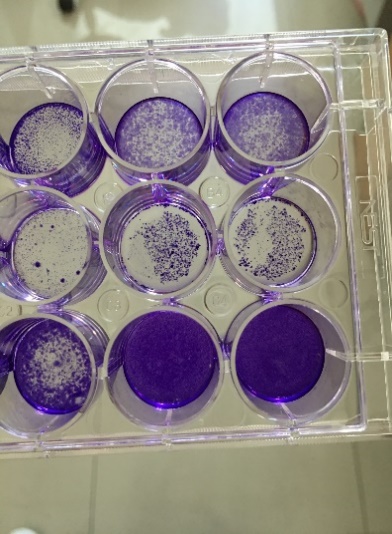

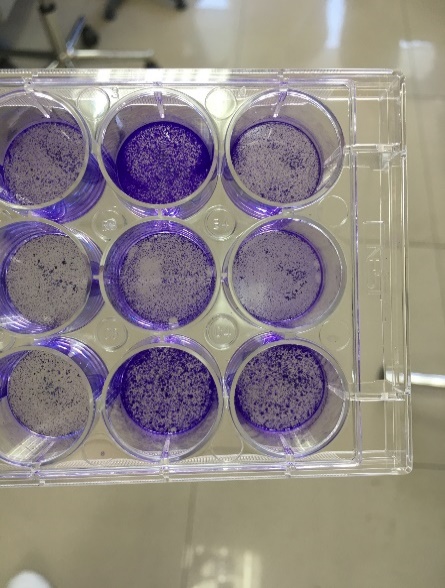

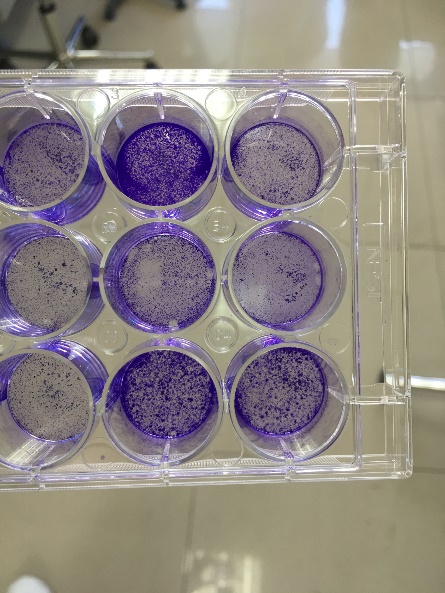

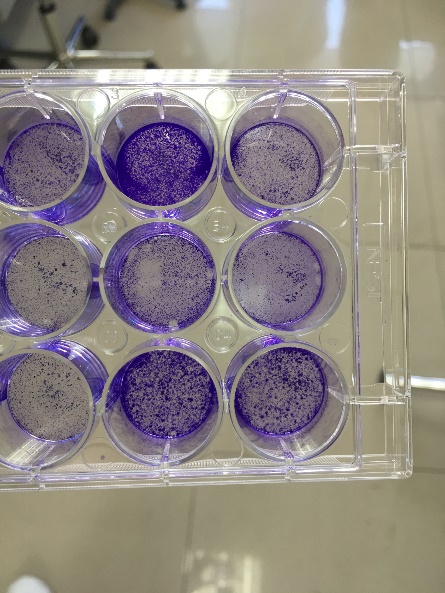

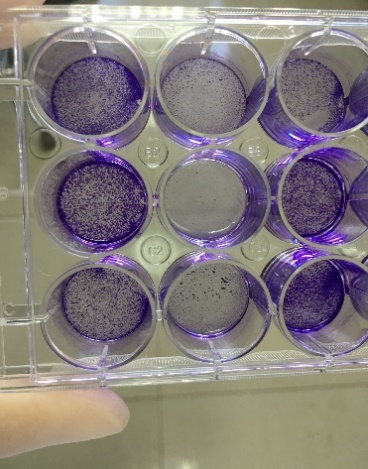

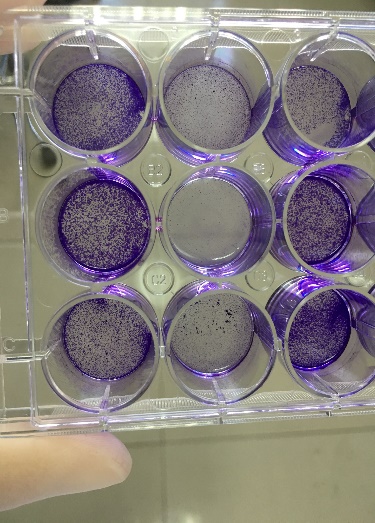

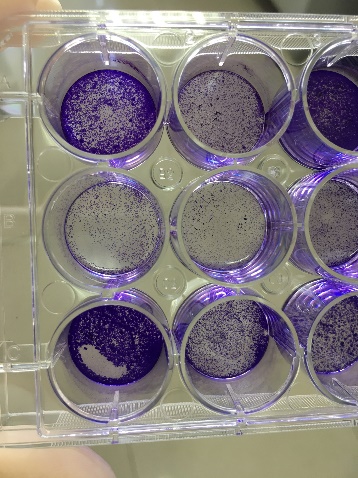

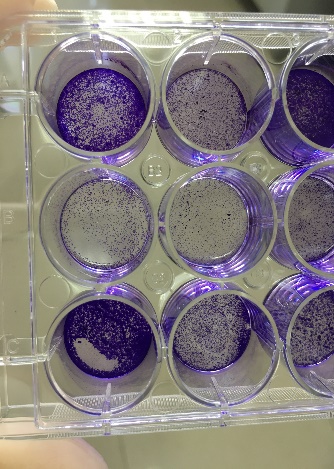

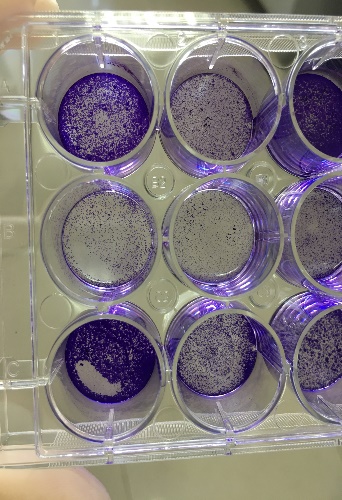

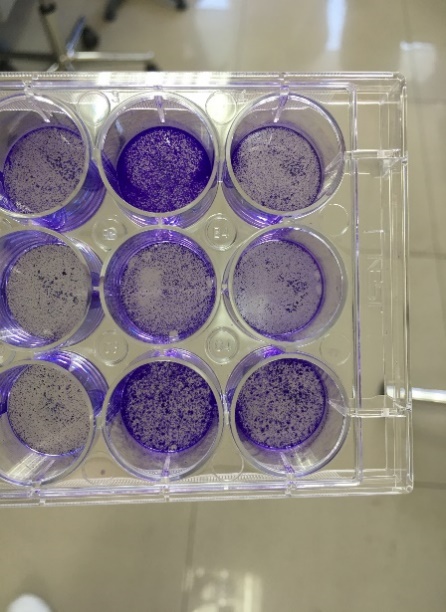

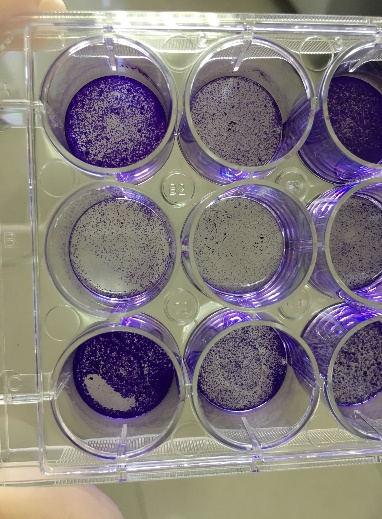


**SW480**

**T47D**

**M21**

**PANC-1**

**HCT116**

**PC-3**

**A549**

**HCC827**

**Caco-2**

**SW1116**


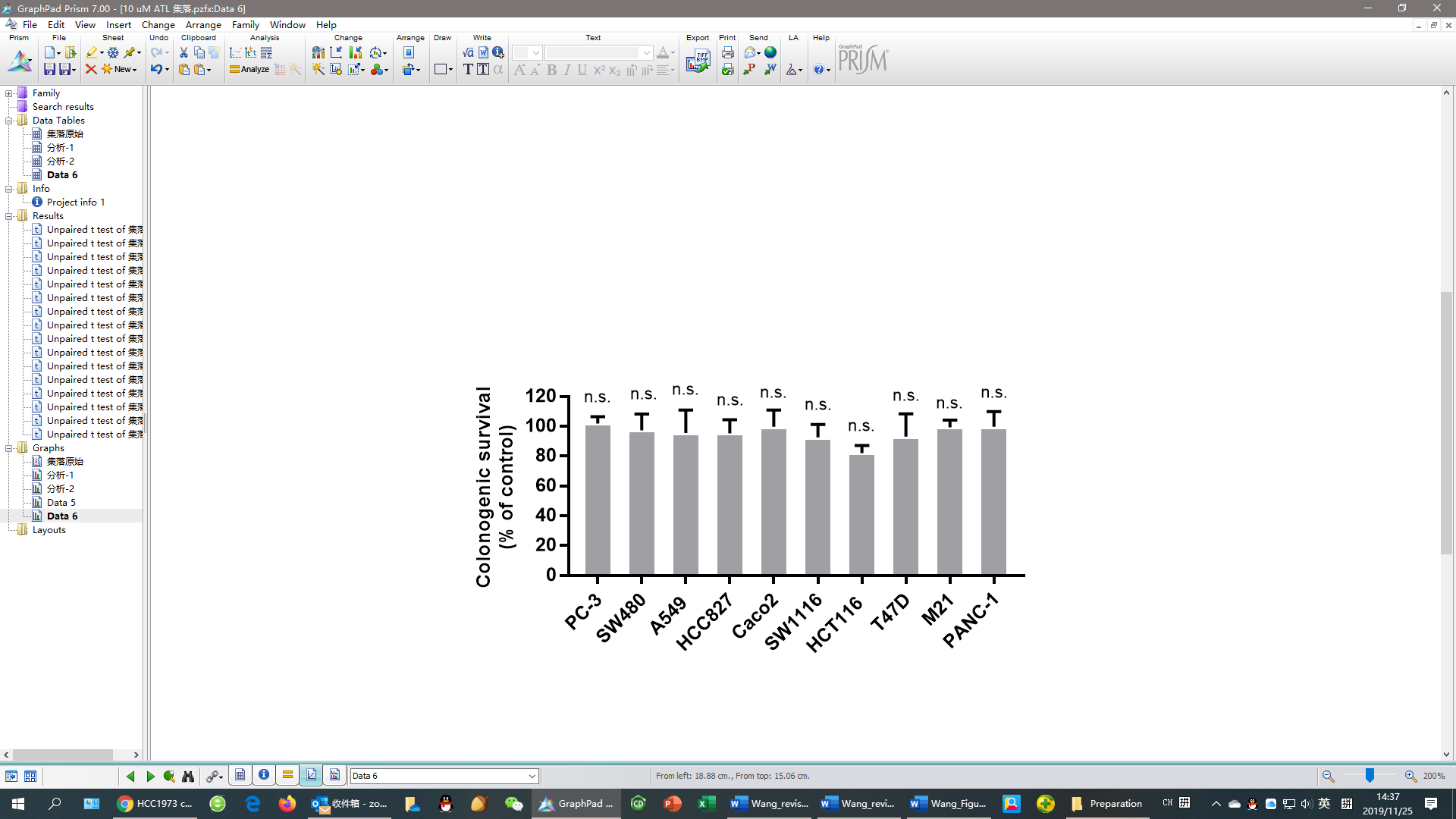


**C**


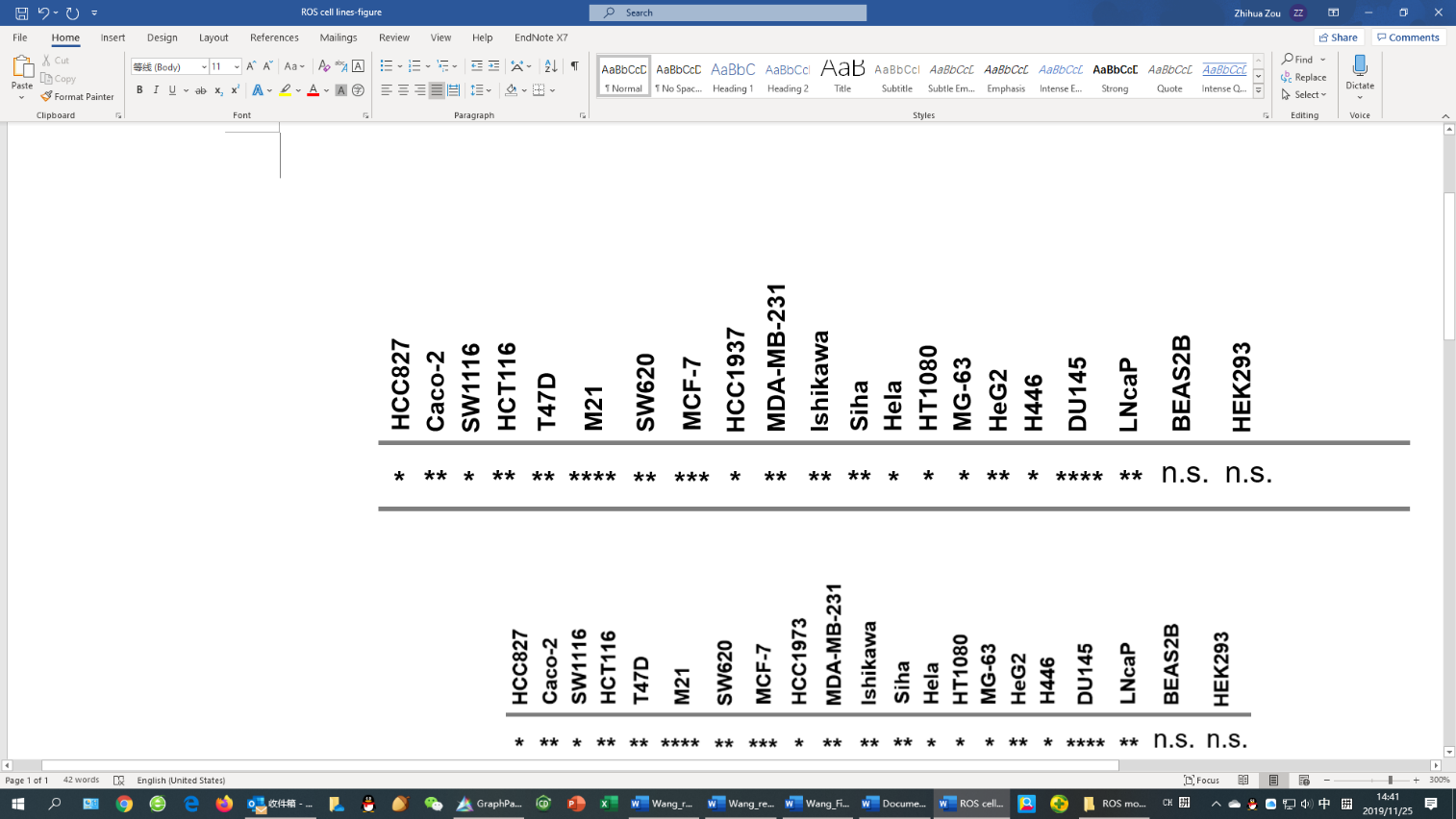


**Figure S1**

Supplement: Supplementary file 2 — Supplementary figure 1 [file 41388_2020_1191_MOESM2_ESM.docx]

**Figure S2**

**A549**

**SW480**

control

ATL


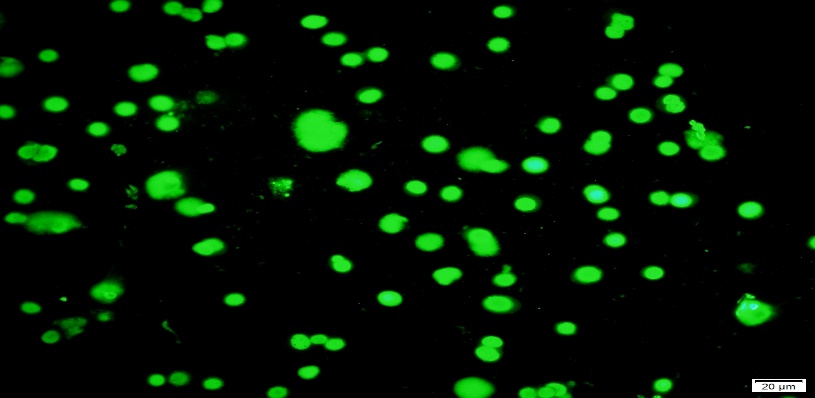

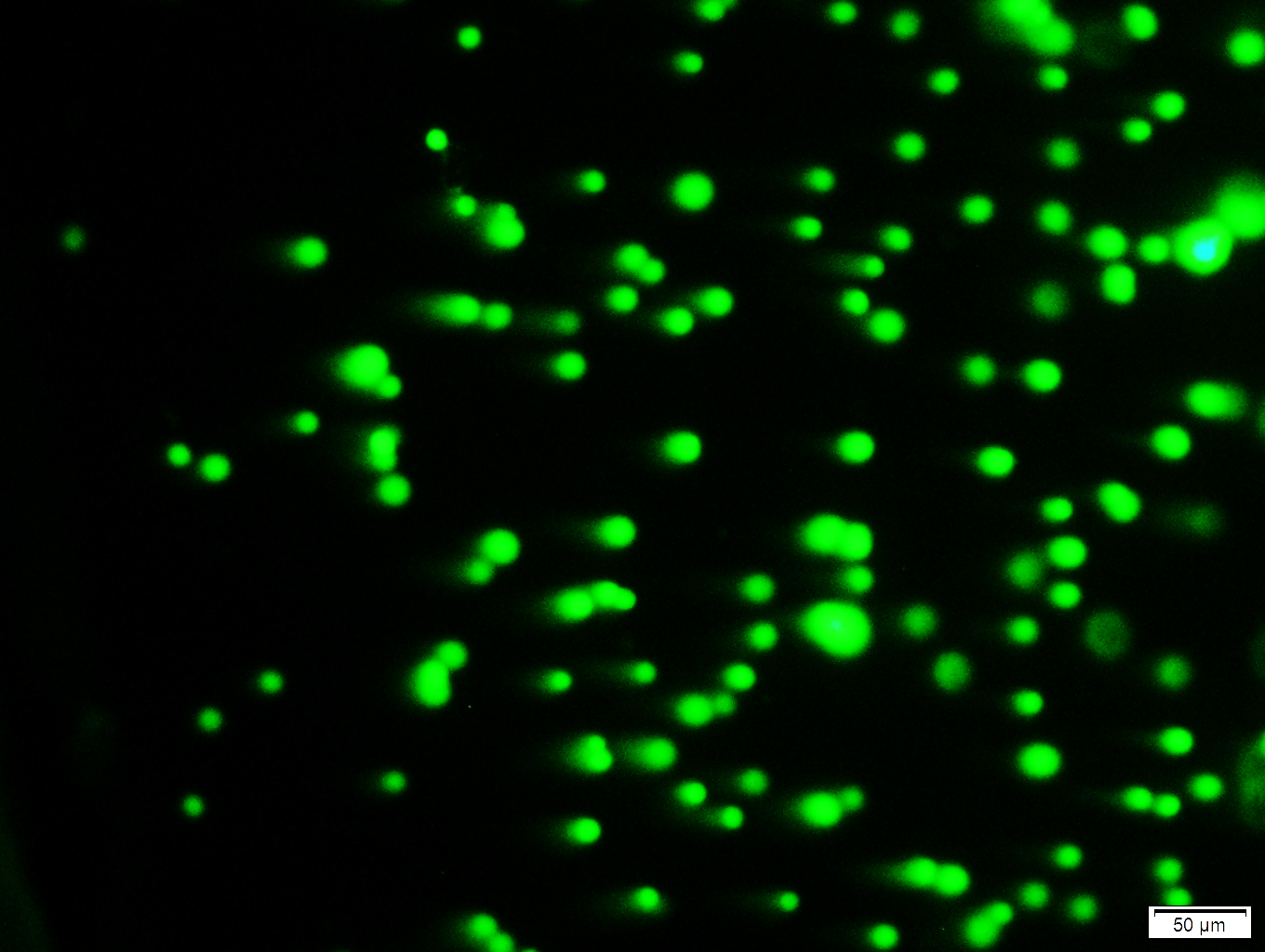

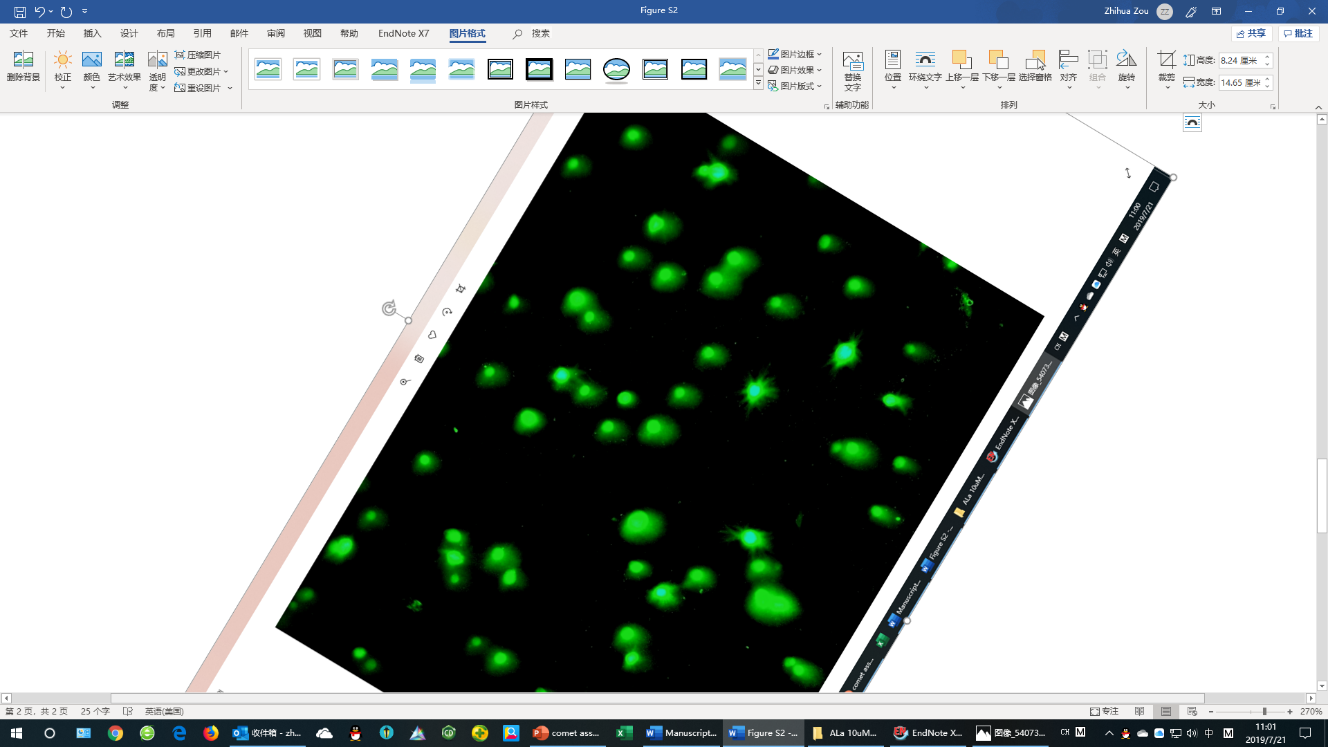

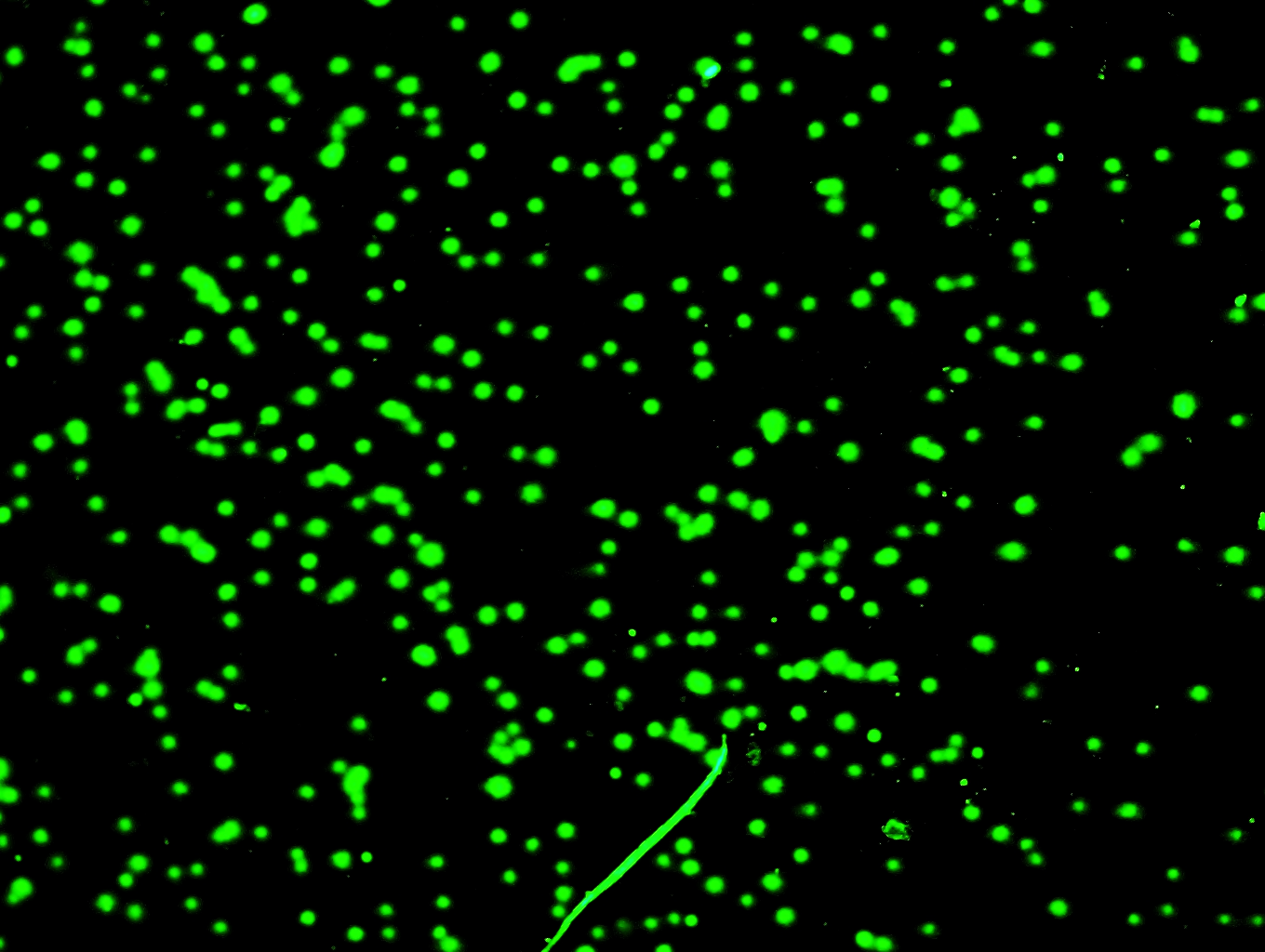


**A549**

**SW480**

**B**

PAR

β-actin

0 h

6 h

12 h

170 kDa

PARP1


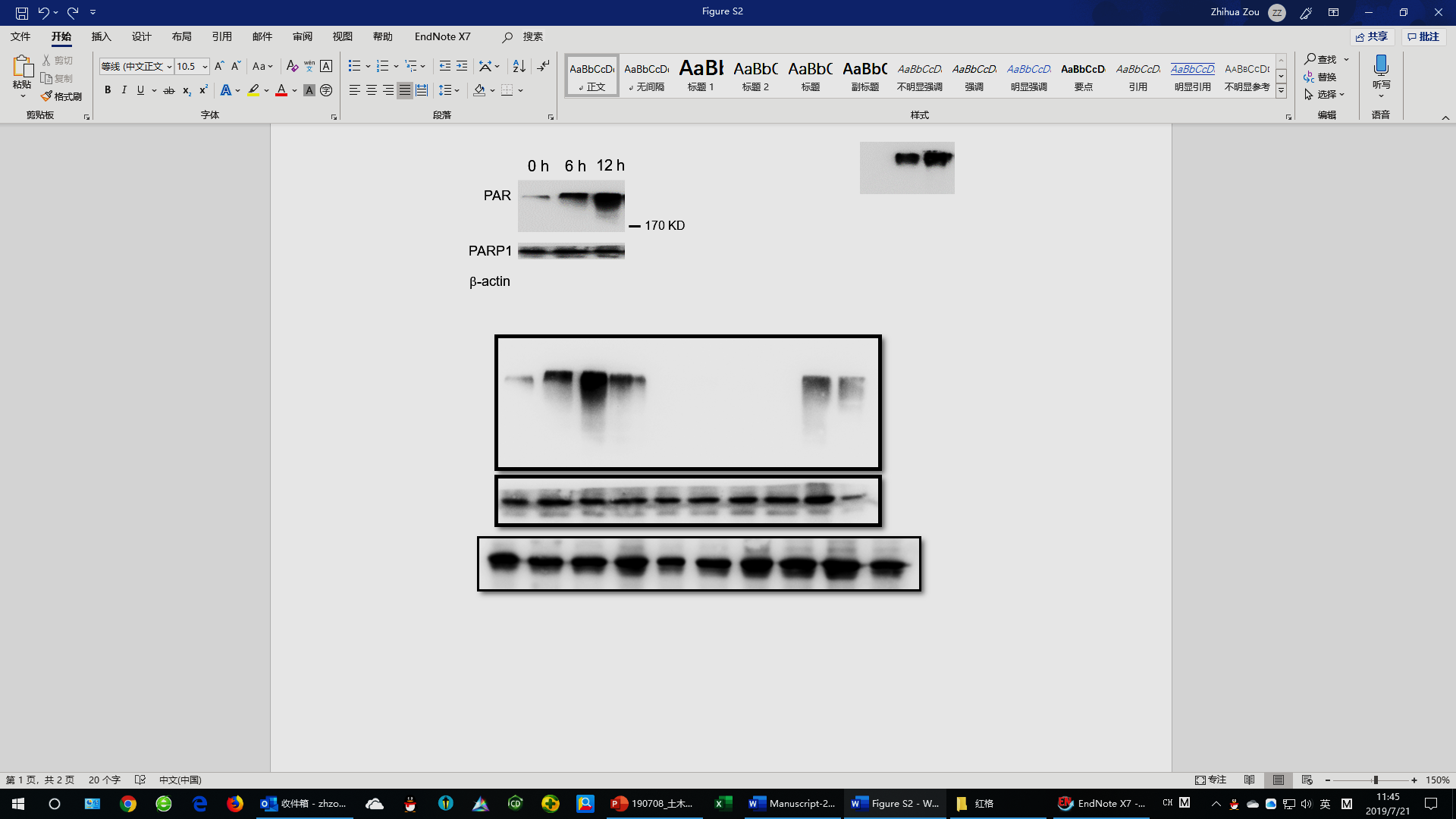

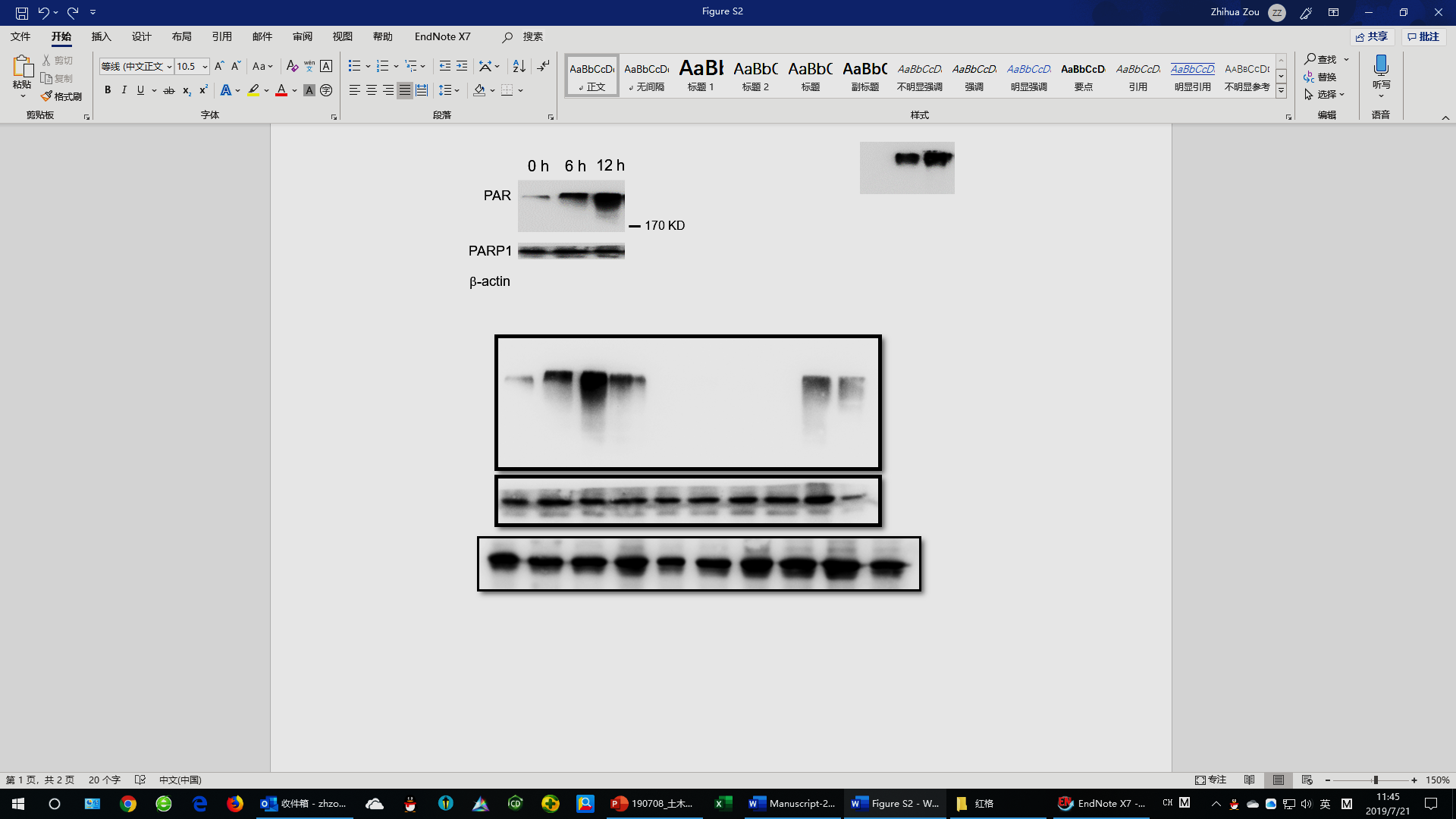

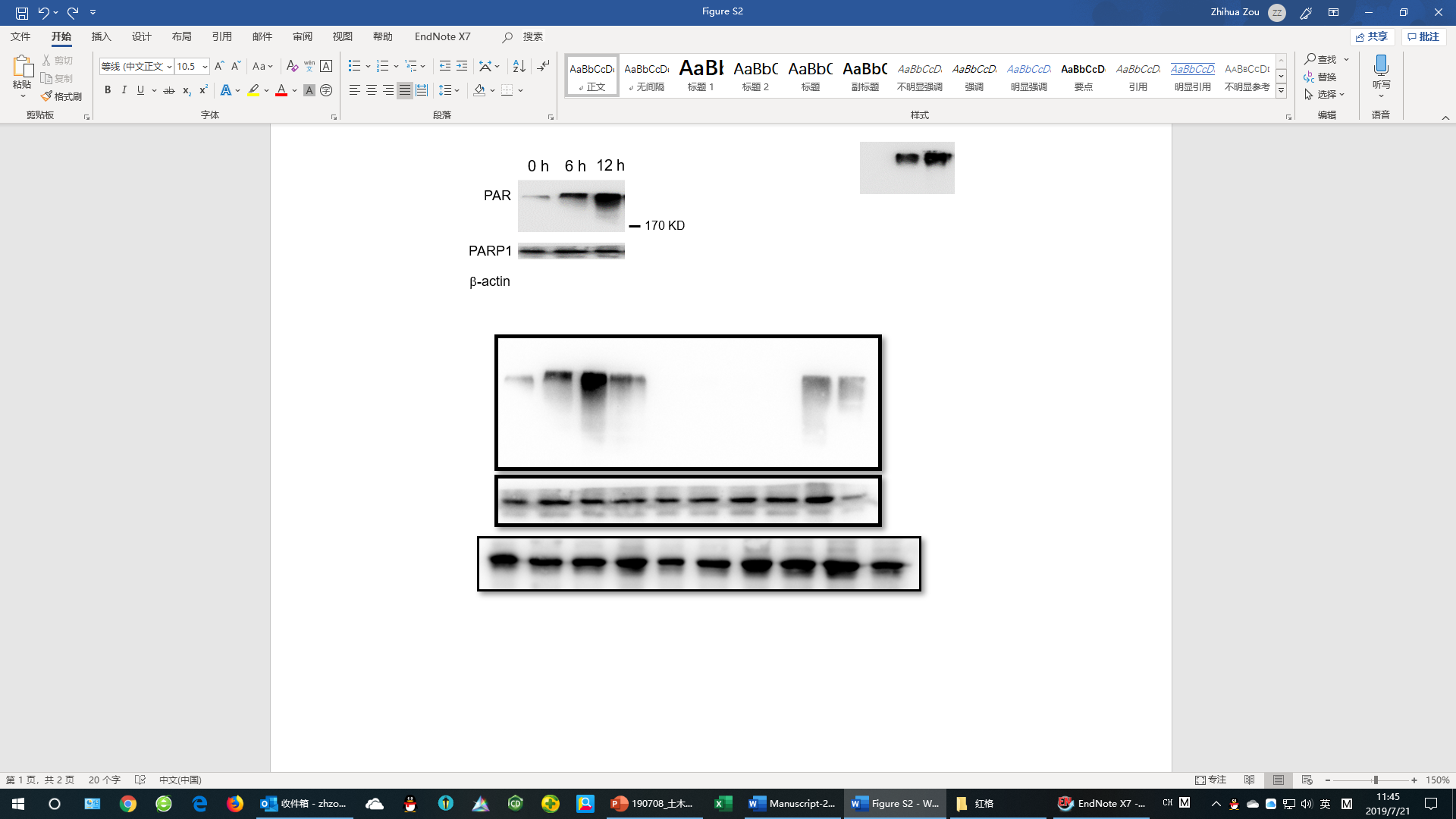

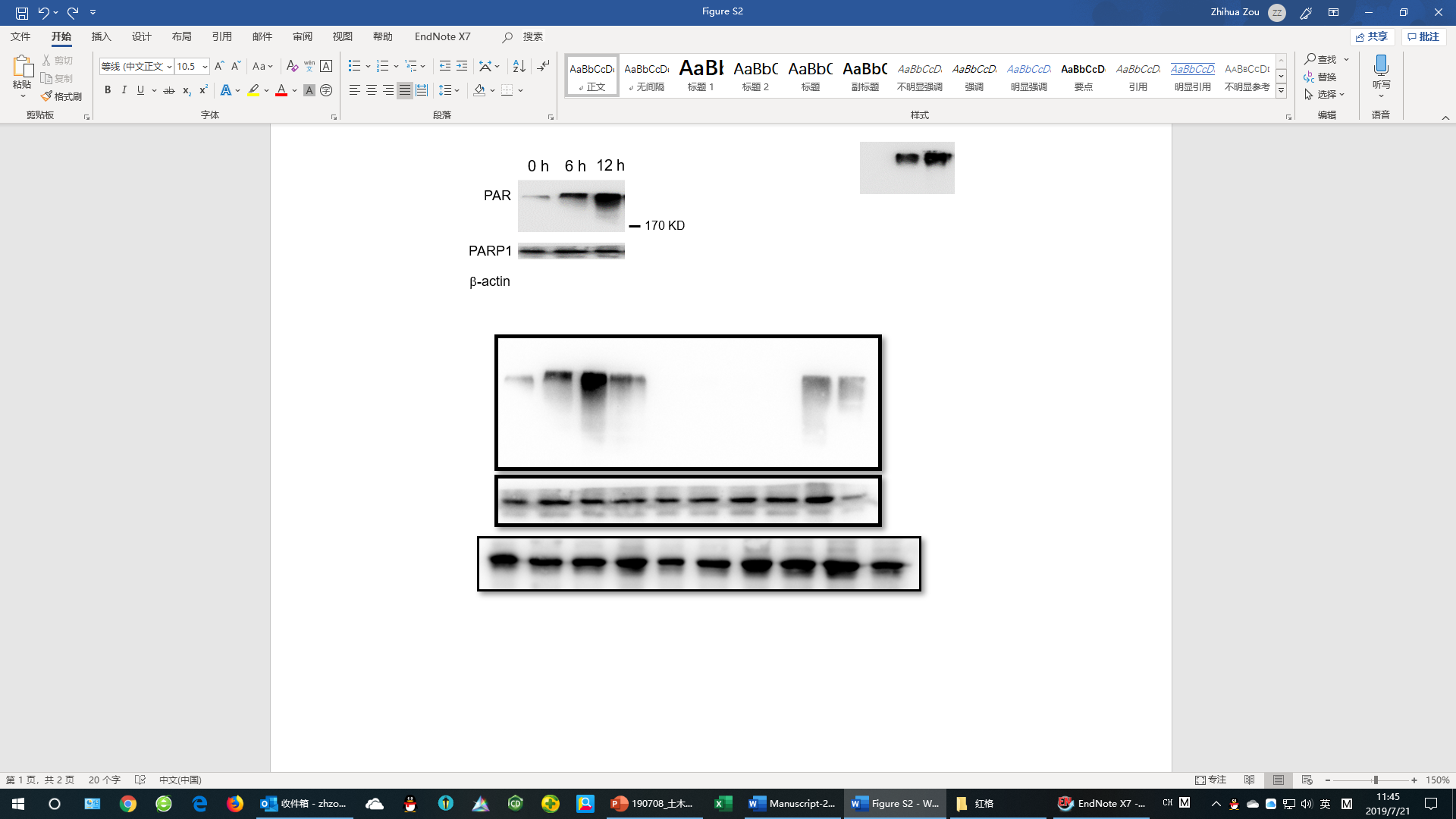

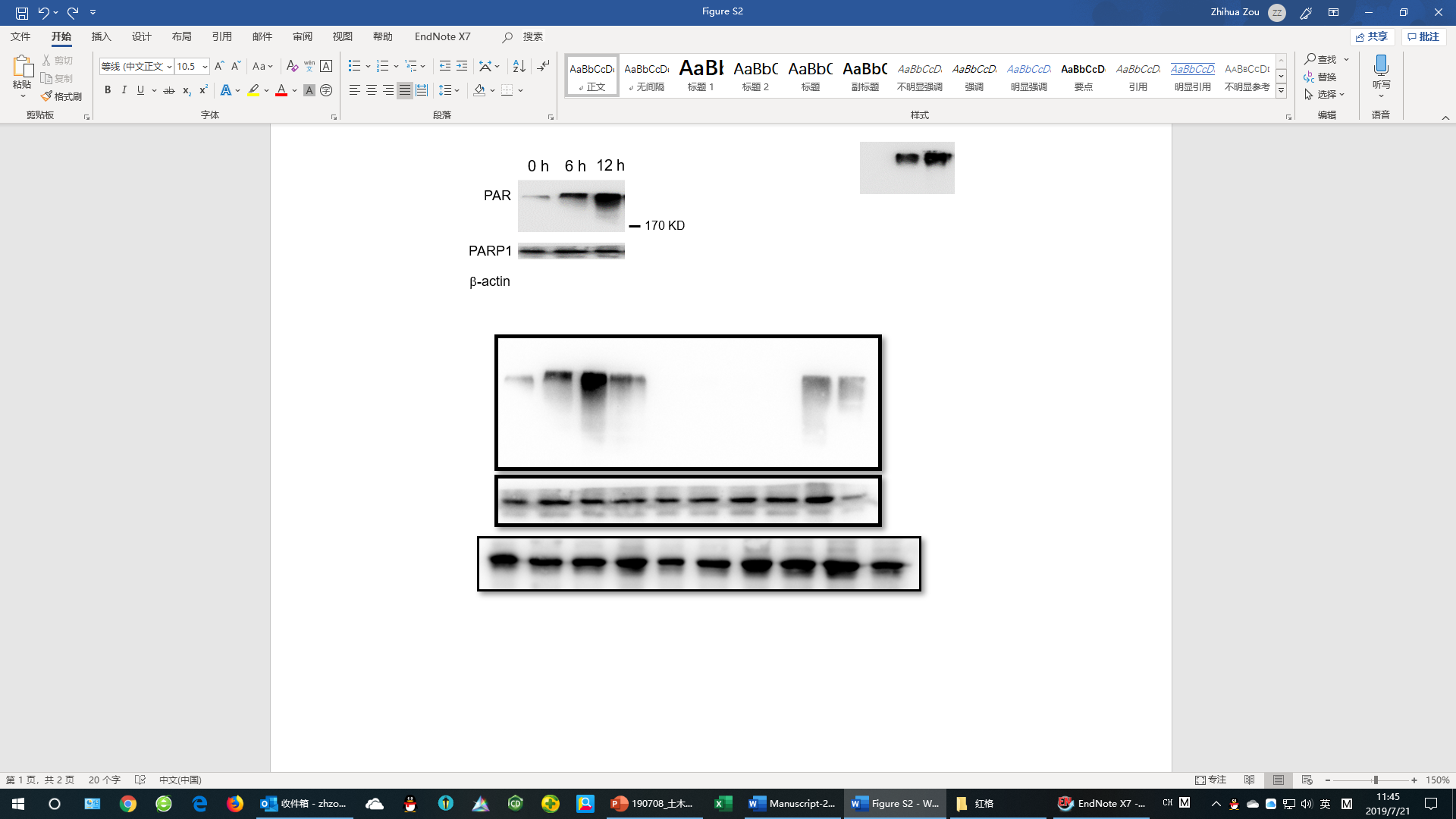

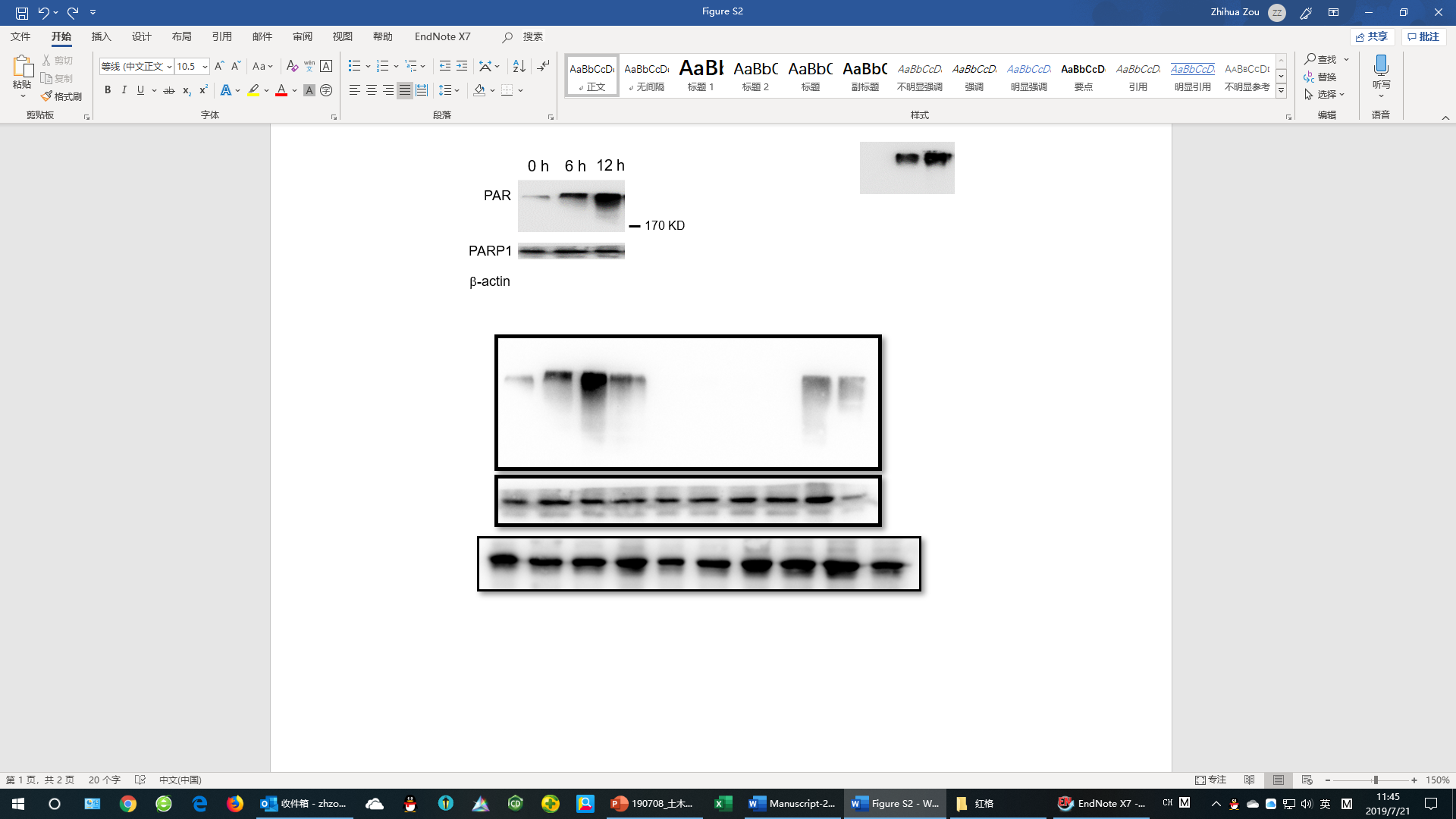


6 h

12 h

+ NAC

**A549**


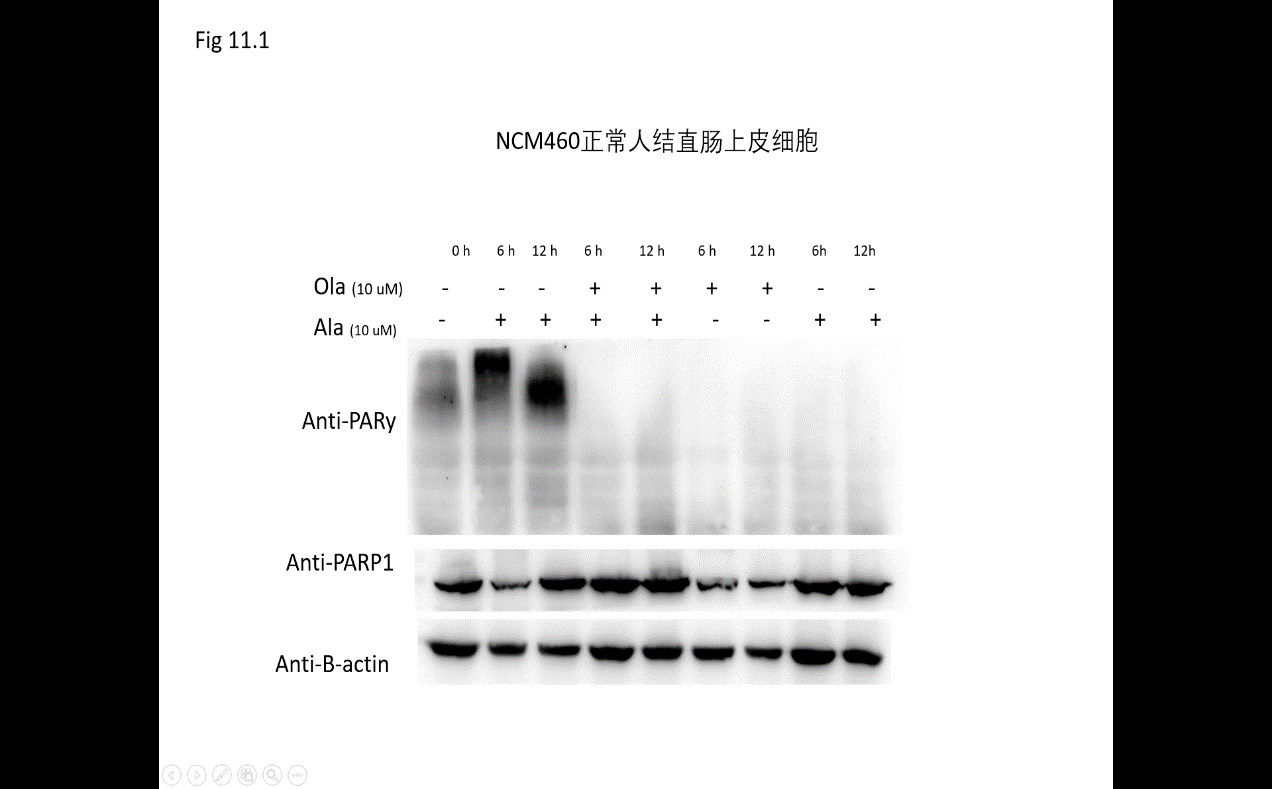


**NCM460**


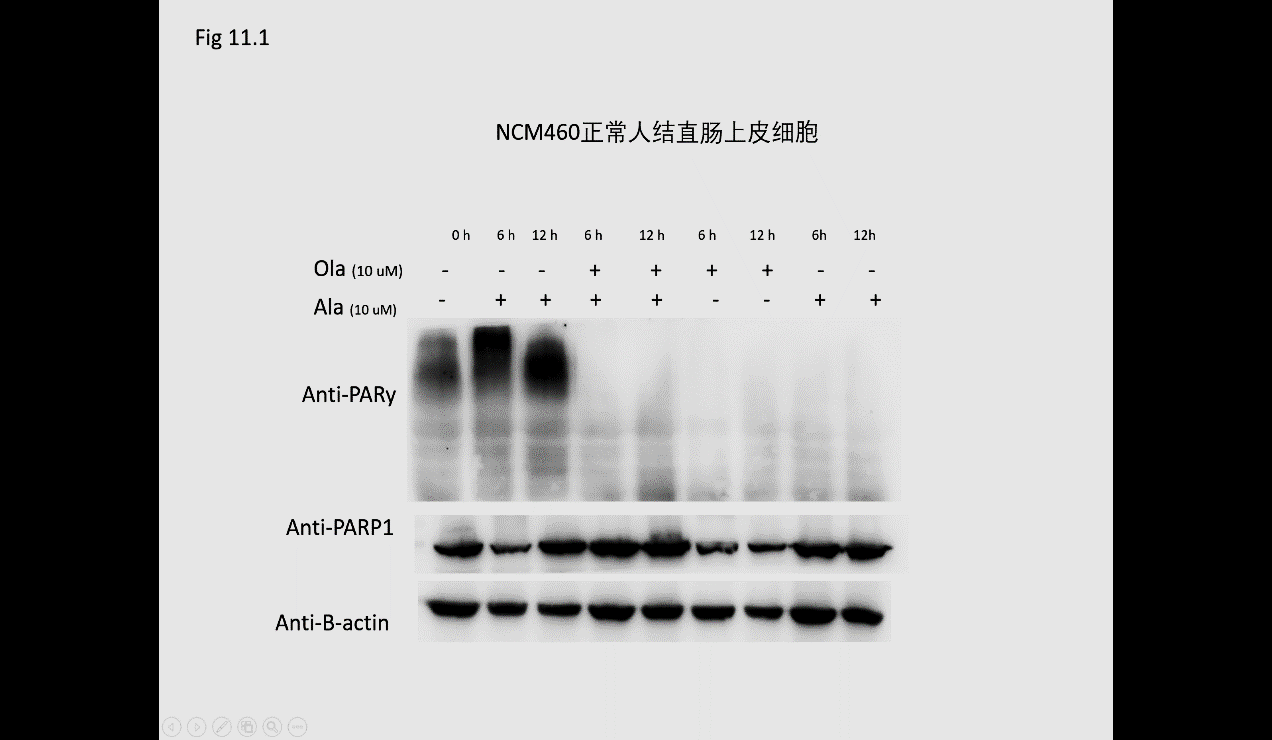


0 h

6 h

12 h

0 h

6 h

12 h

6 h

12 h

+ NAC


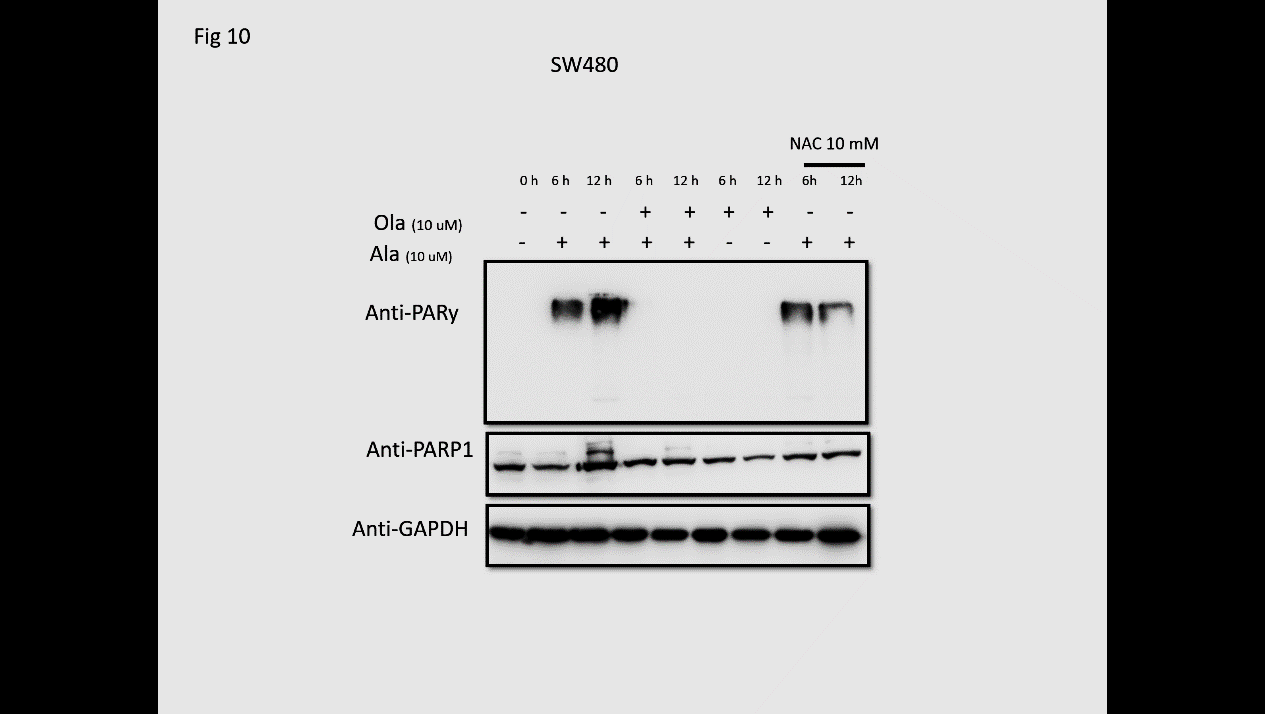

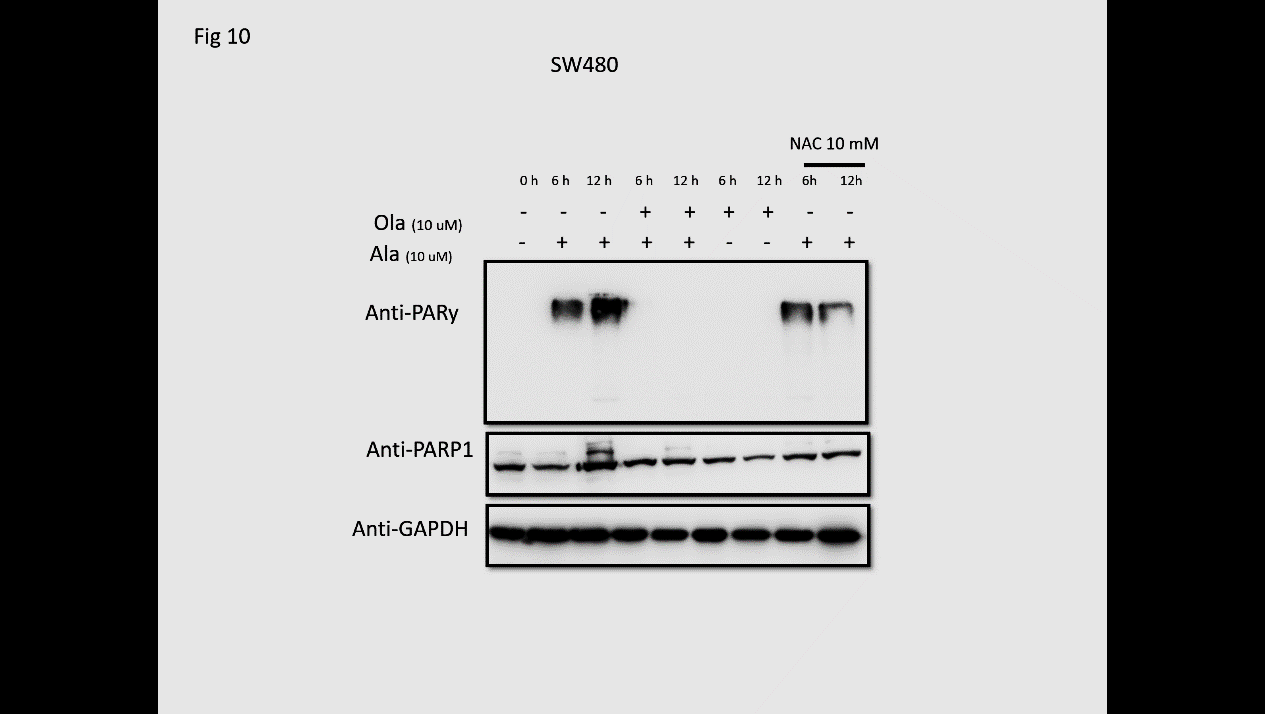

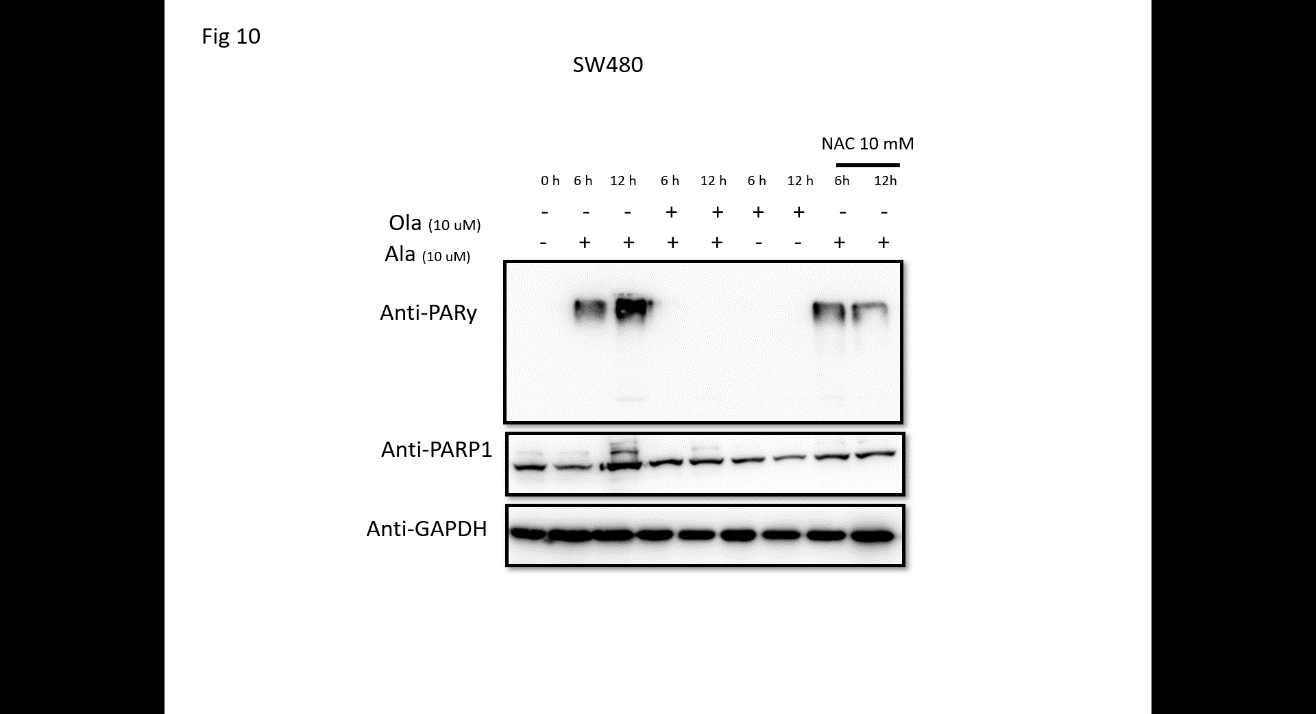

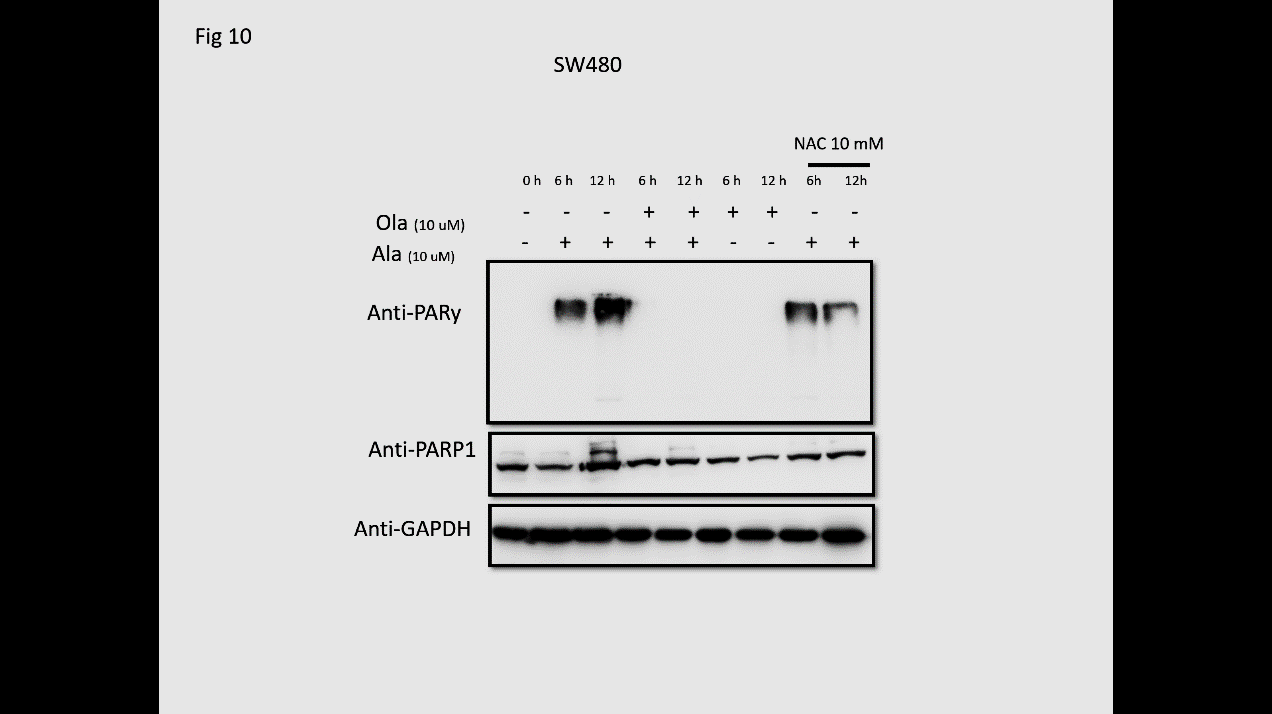

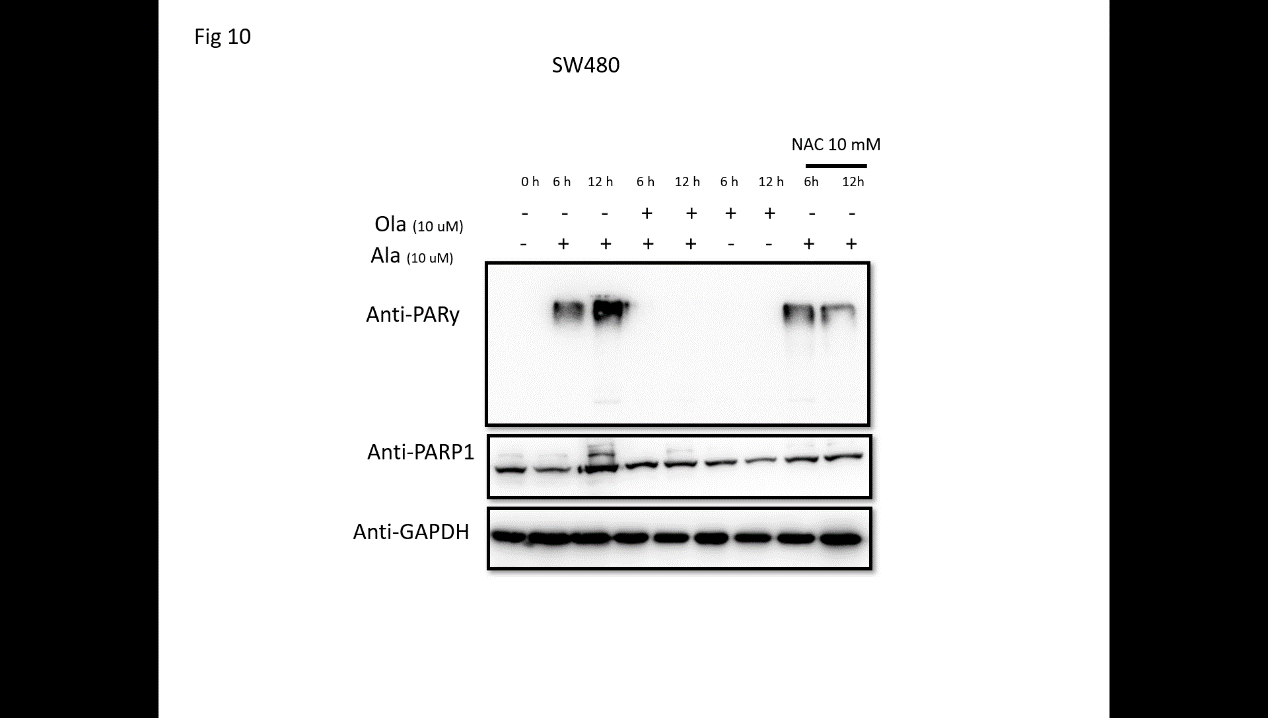

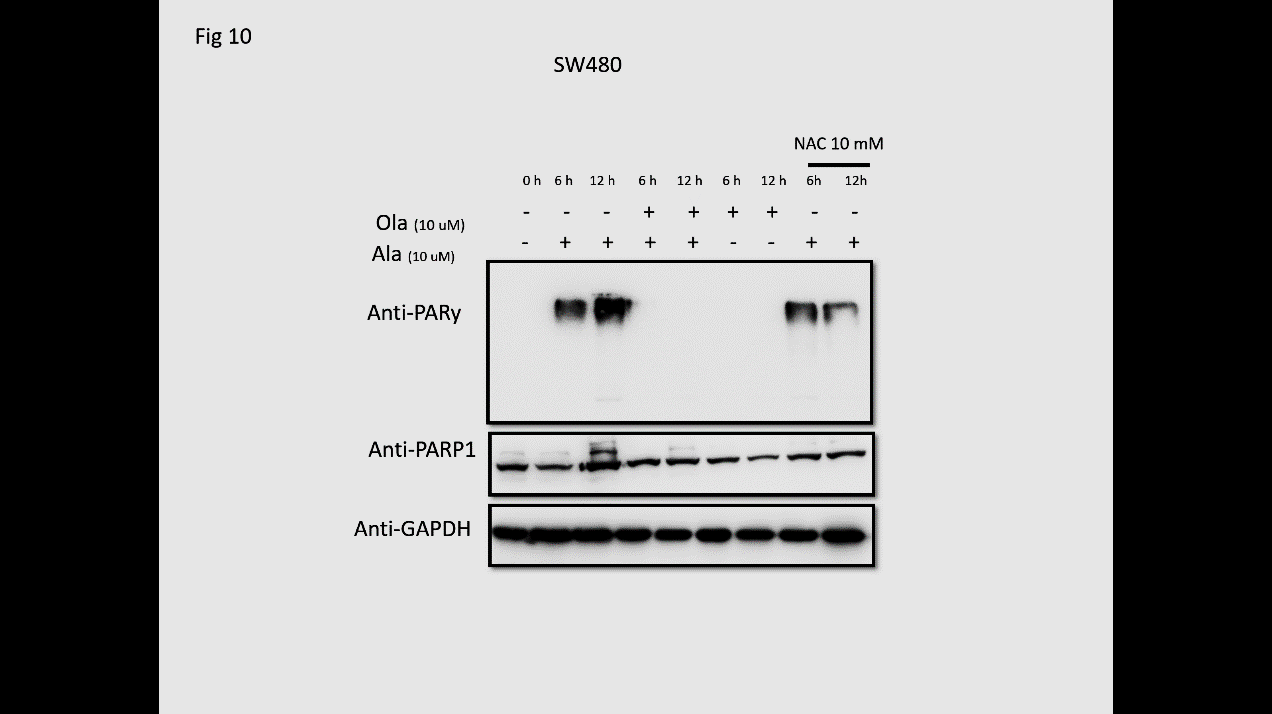


**SW480**

**C**

**A**


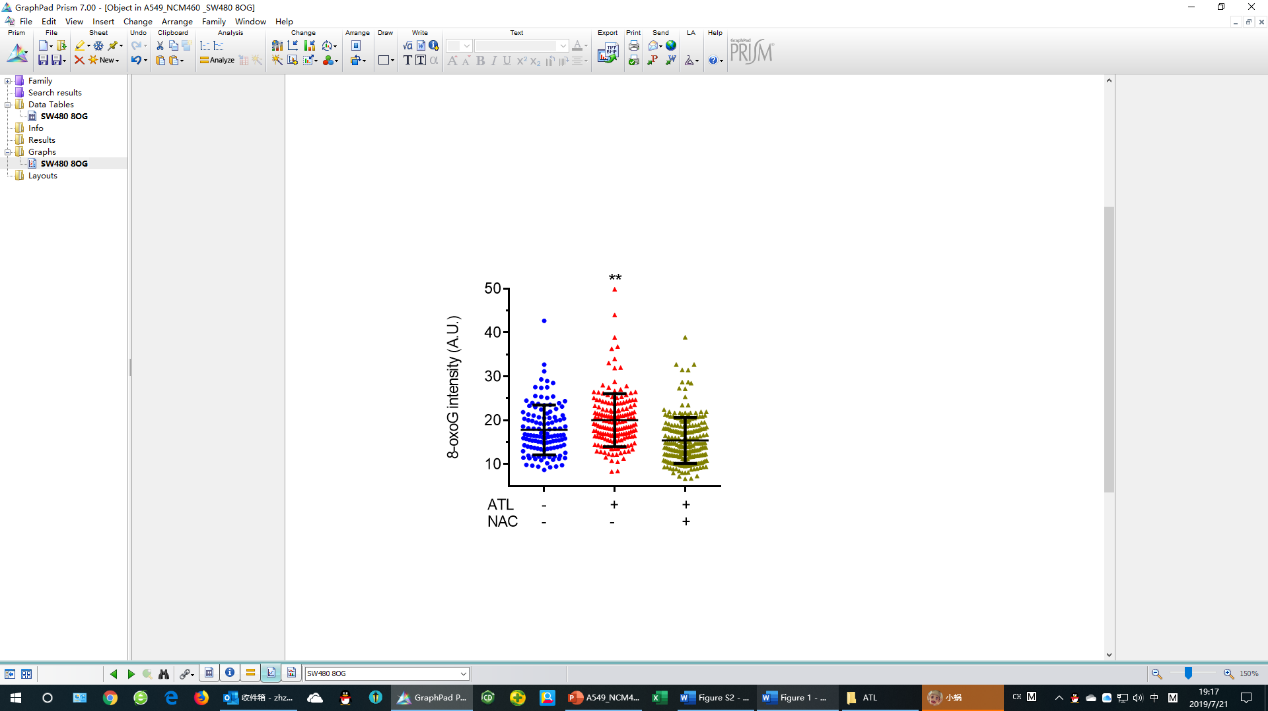

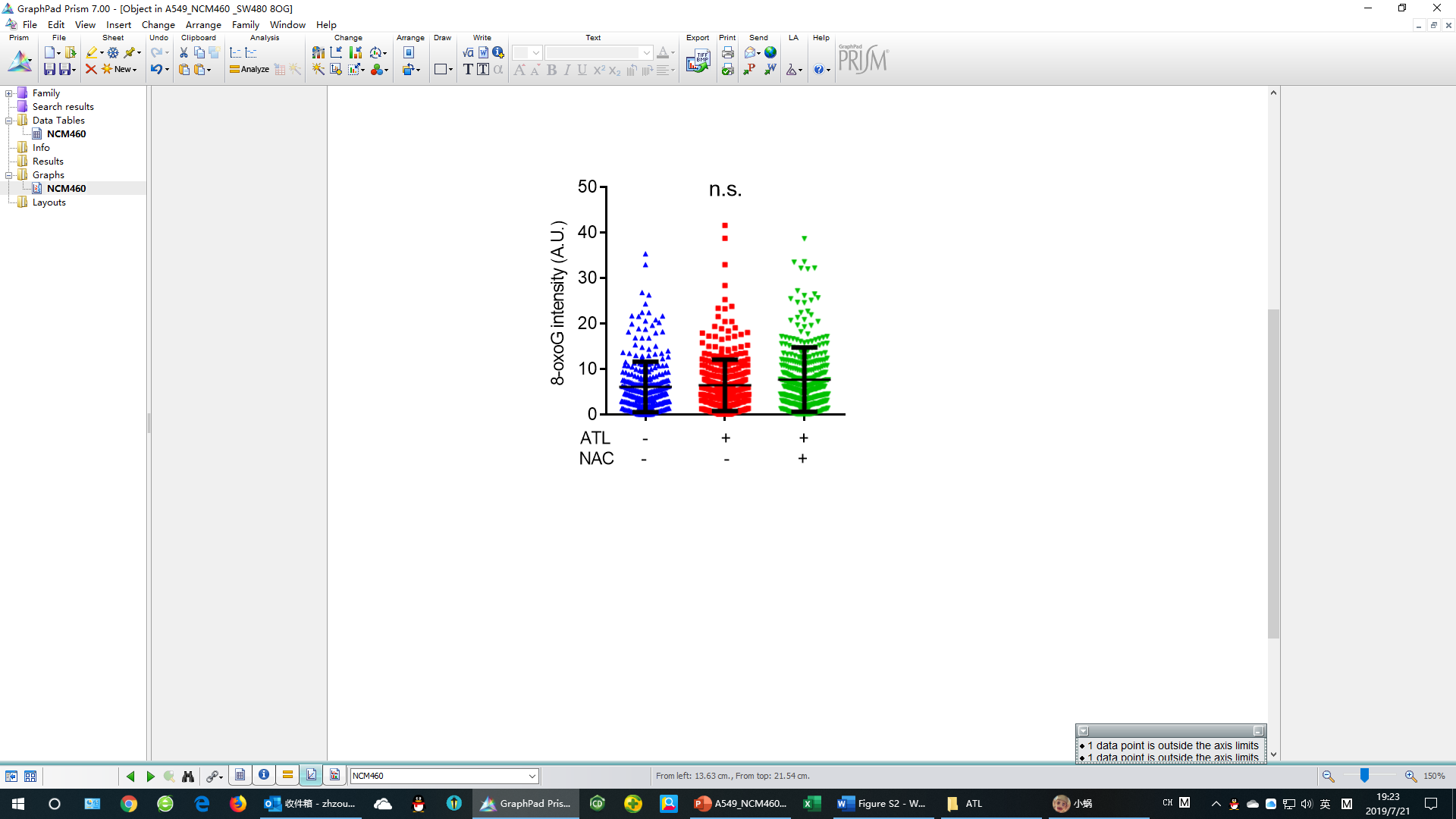

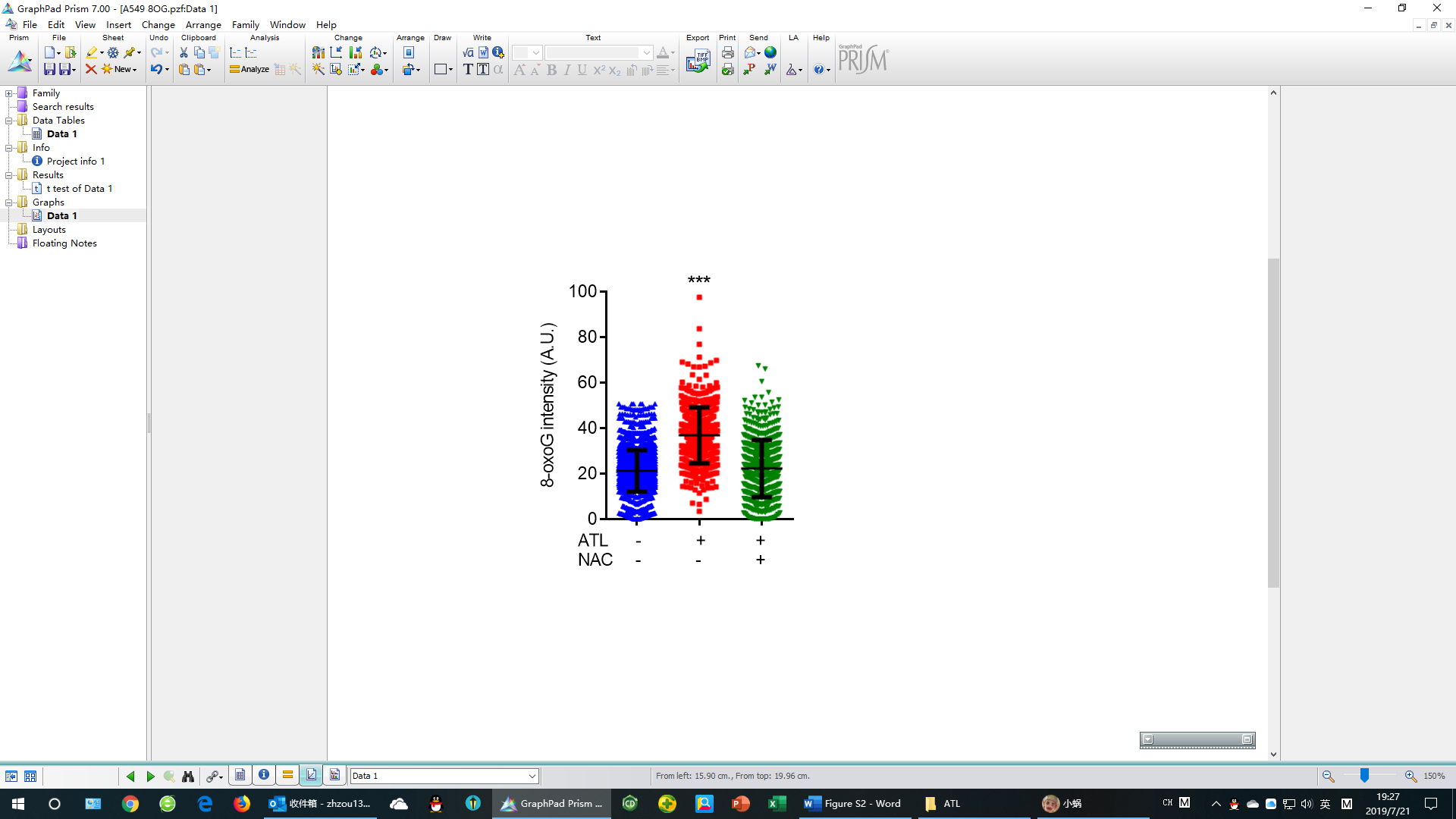


**A549**

**SW480**

**NCM460**


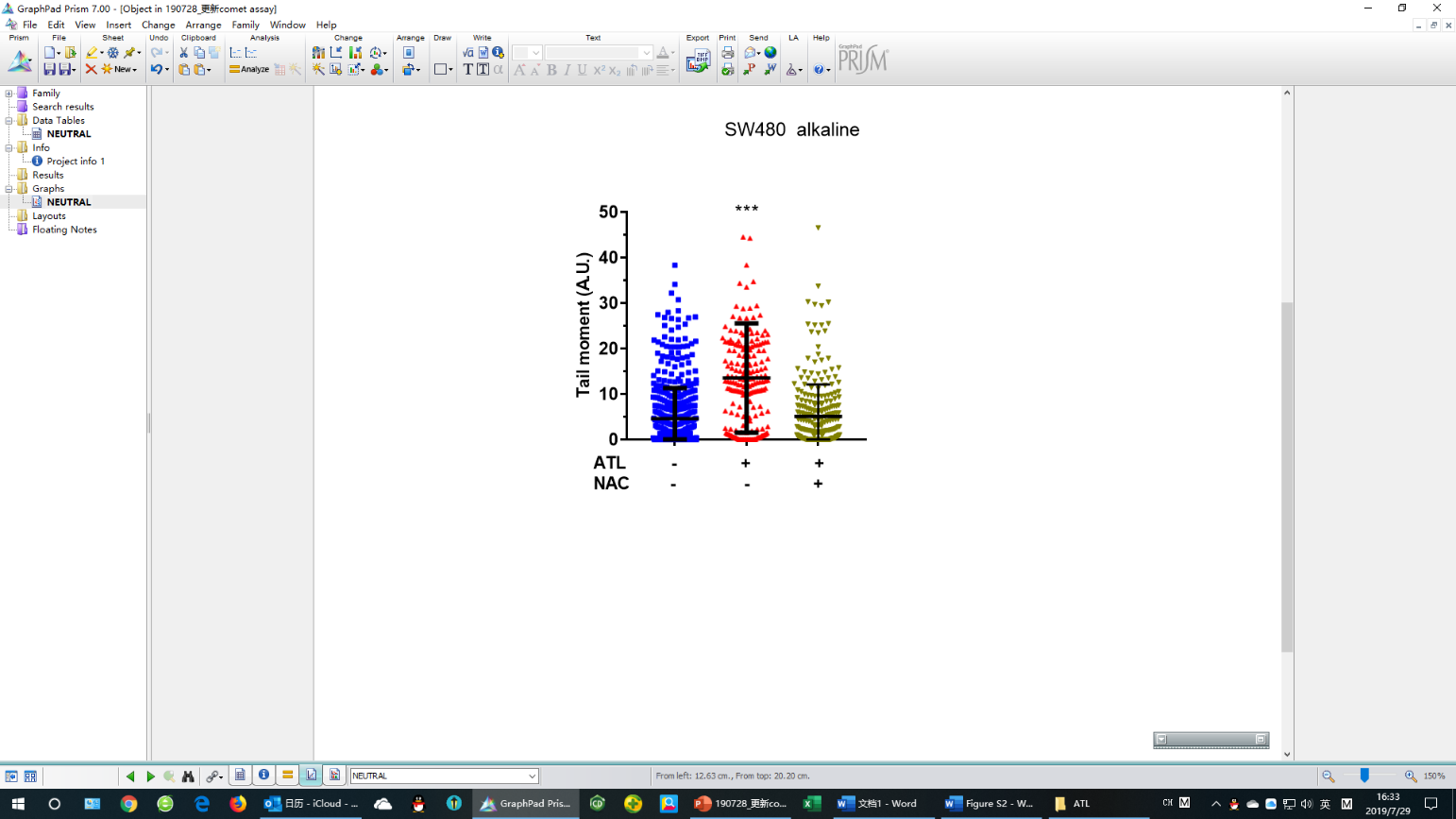

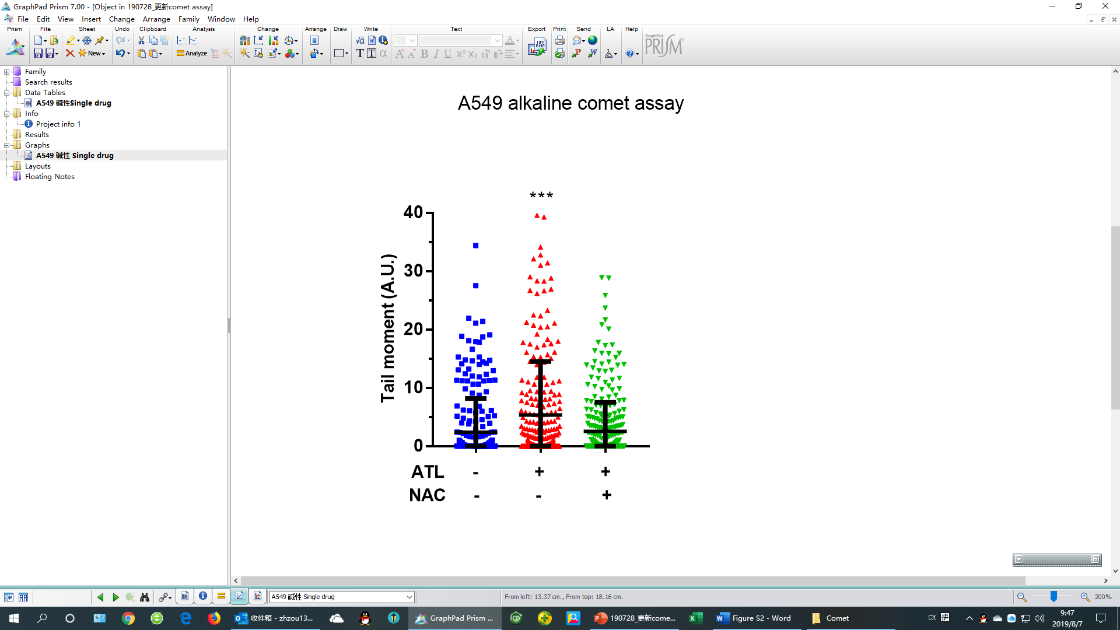

Supplement: Supplementary file 3 — Supplementary figure 2 [file 41388_2020_1191_MOESM3_ESM.docx]

**Figure S3**


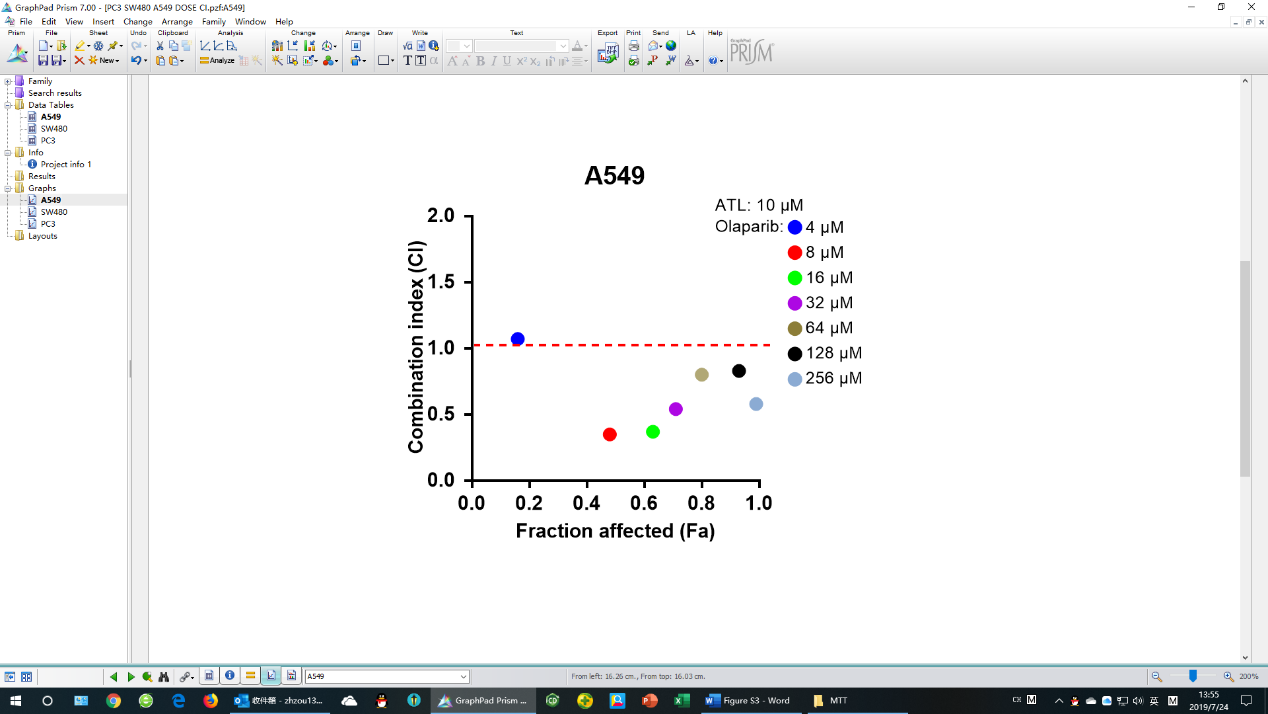


**A549**


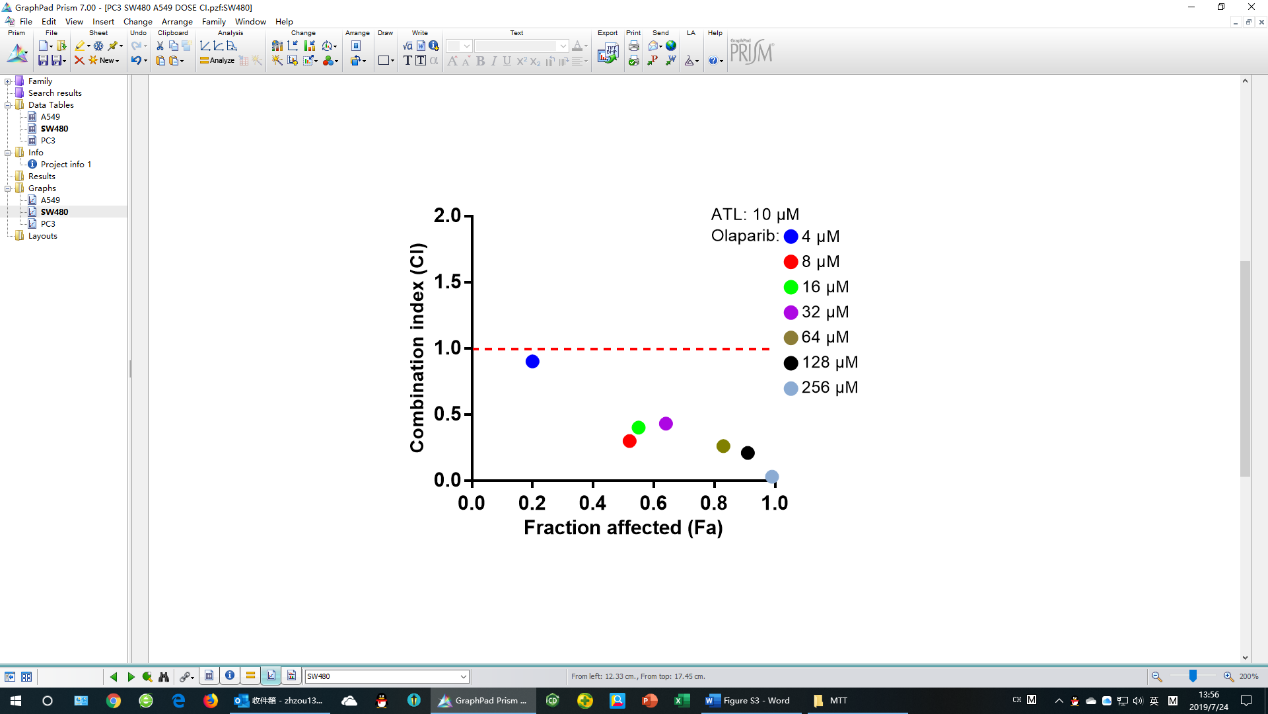


**SW480**

**A**


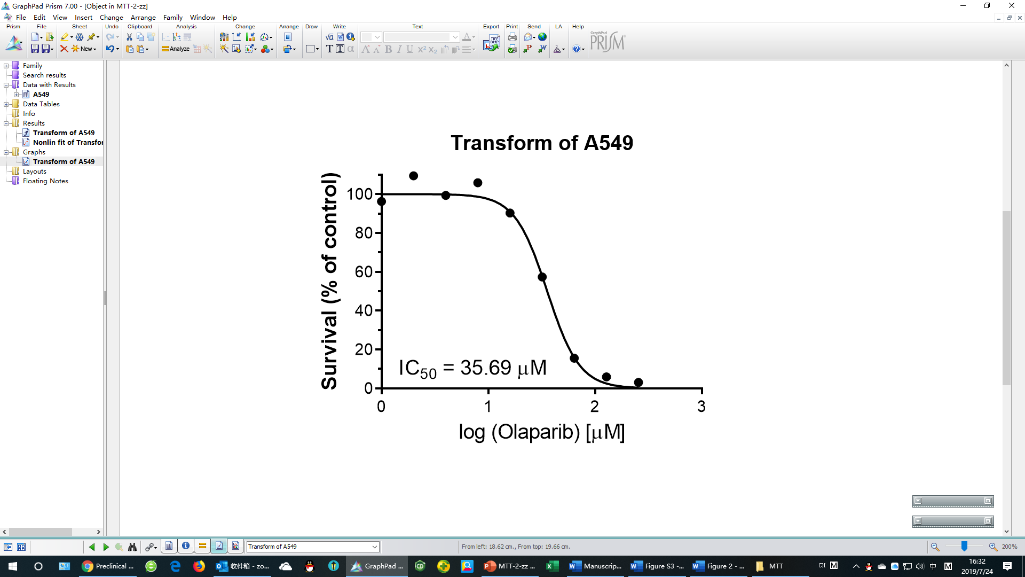


**A549**


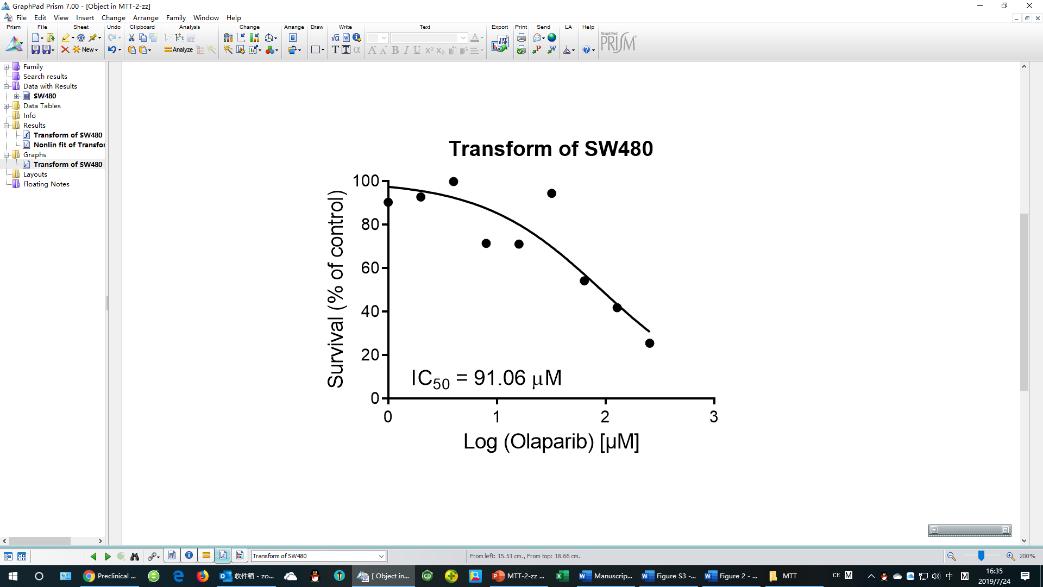


**SW480**

**B**

control

ATL

Ola

ATL+Ola

ATL+Ola

+ NAC

**SW480**

**A549**

**BEAS-2B**


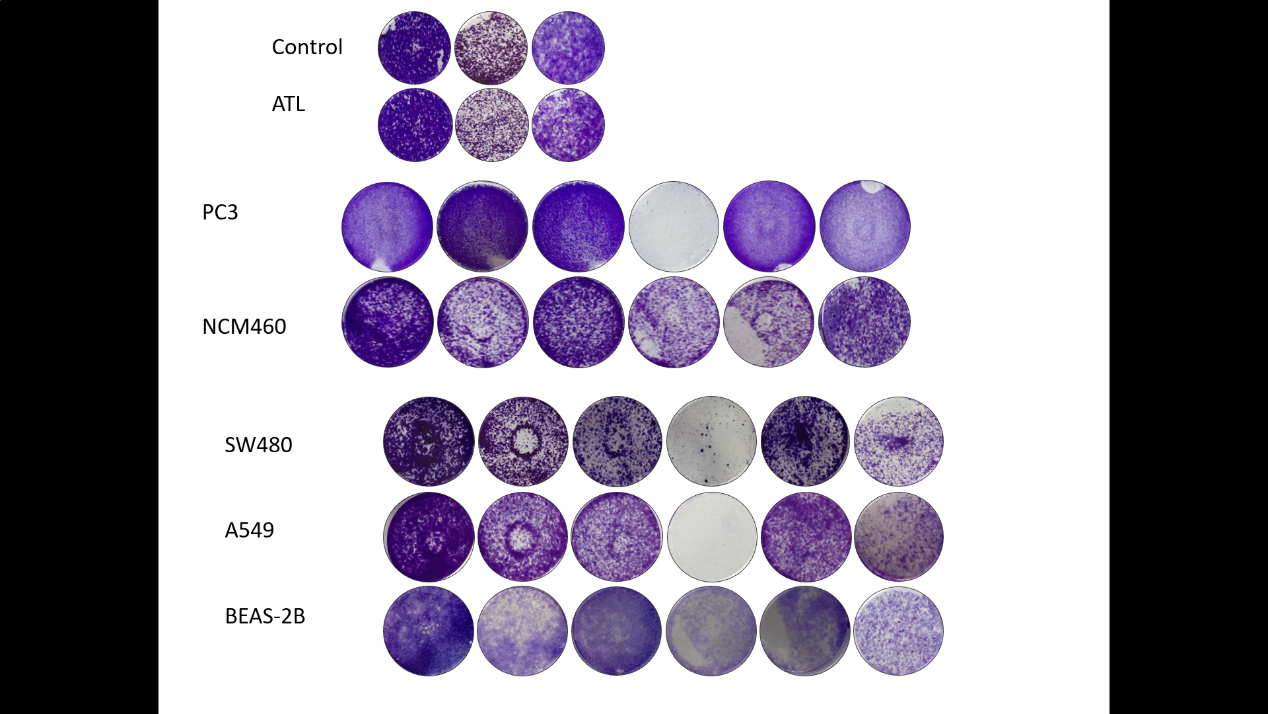


**C**


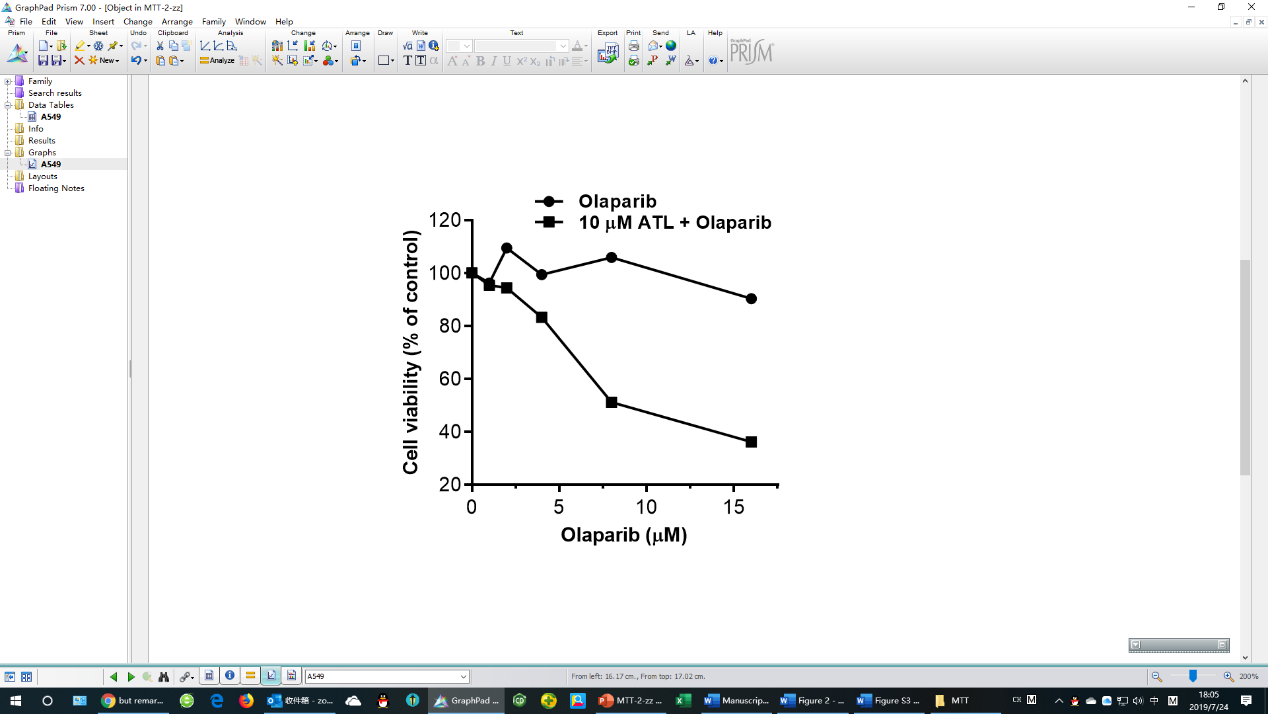


**A549**

**D**


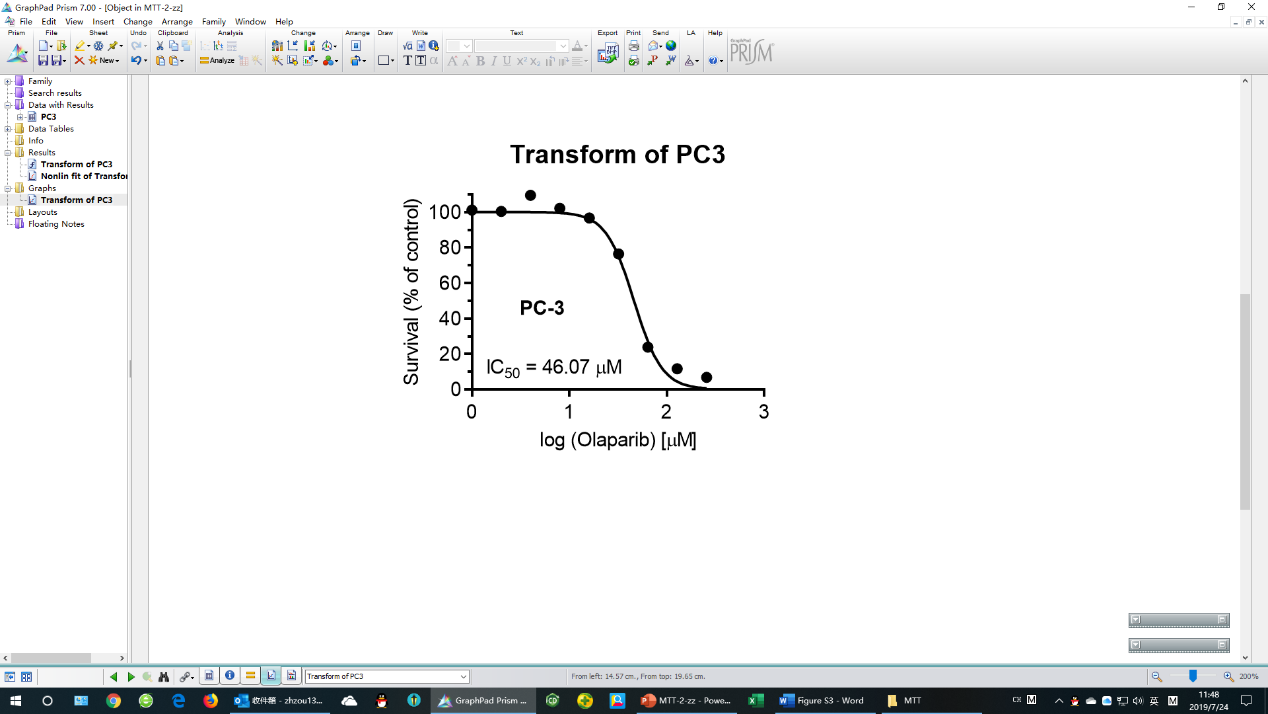


**PC-3**


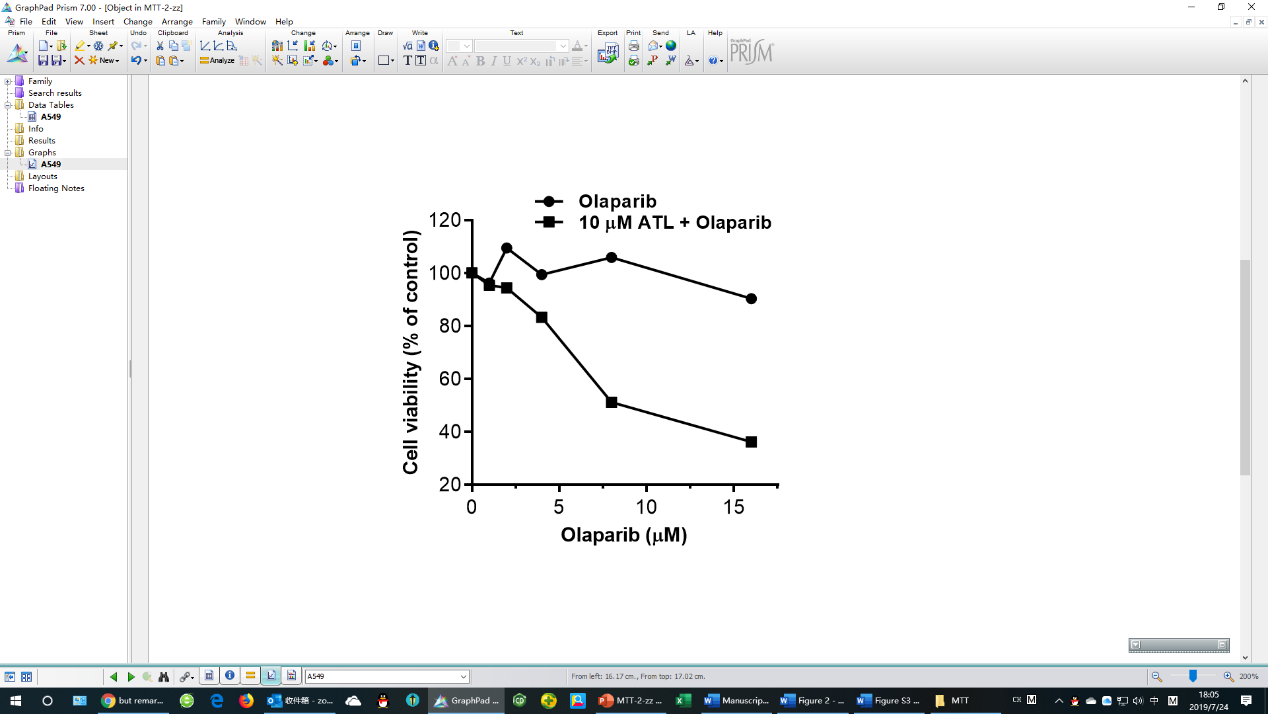


ATL+Vel


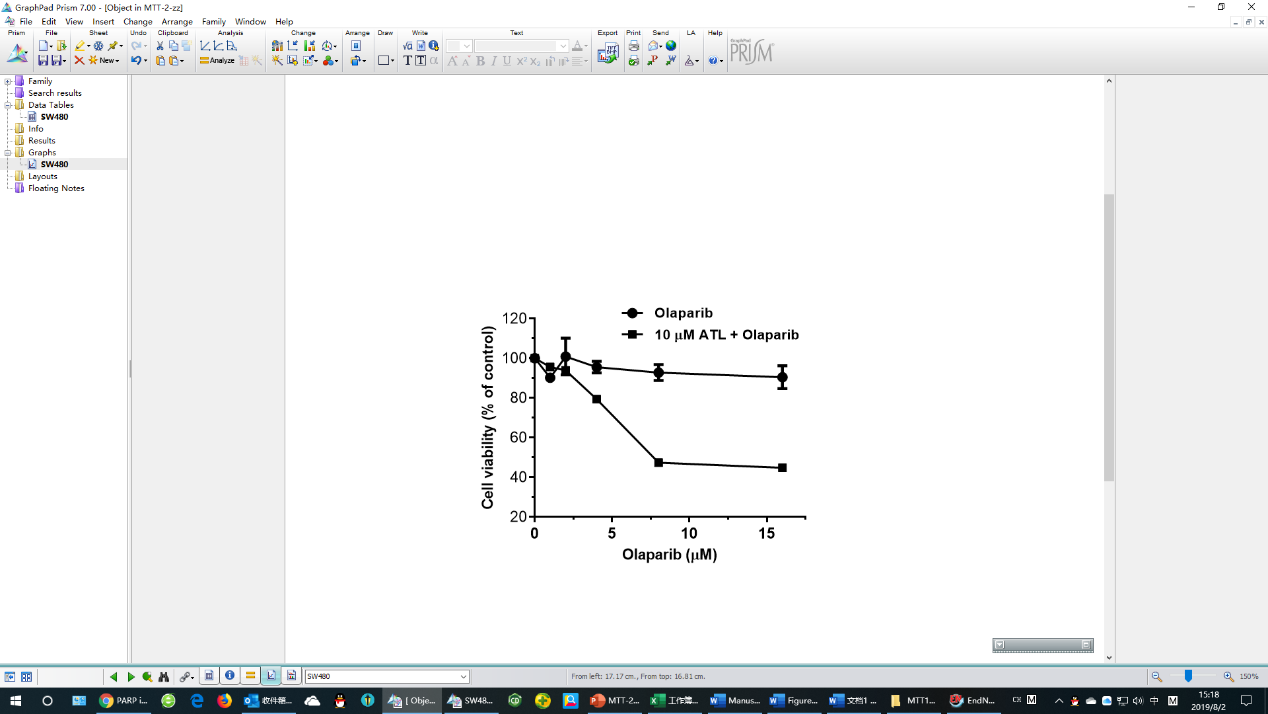


**SW480**


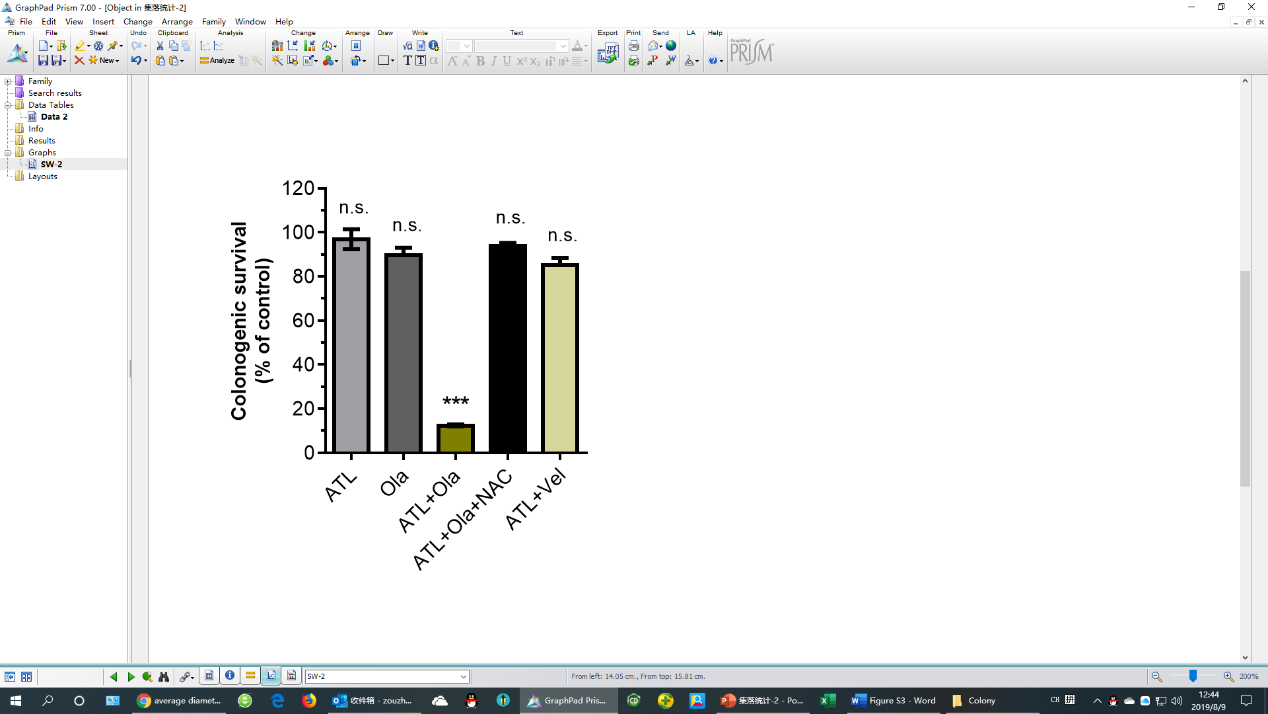


**SW480**


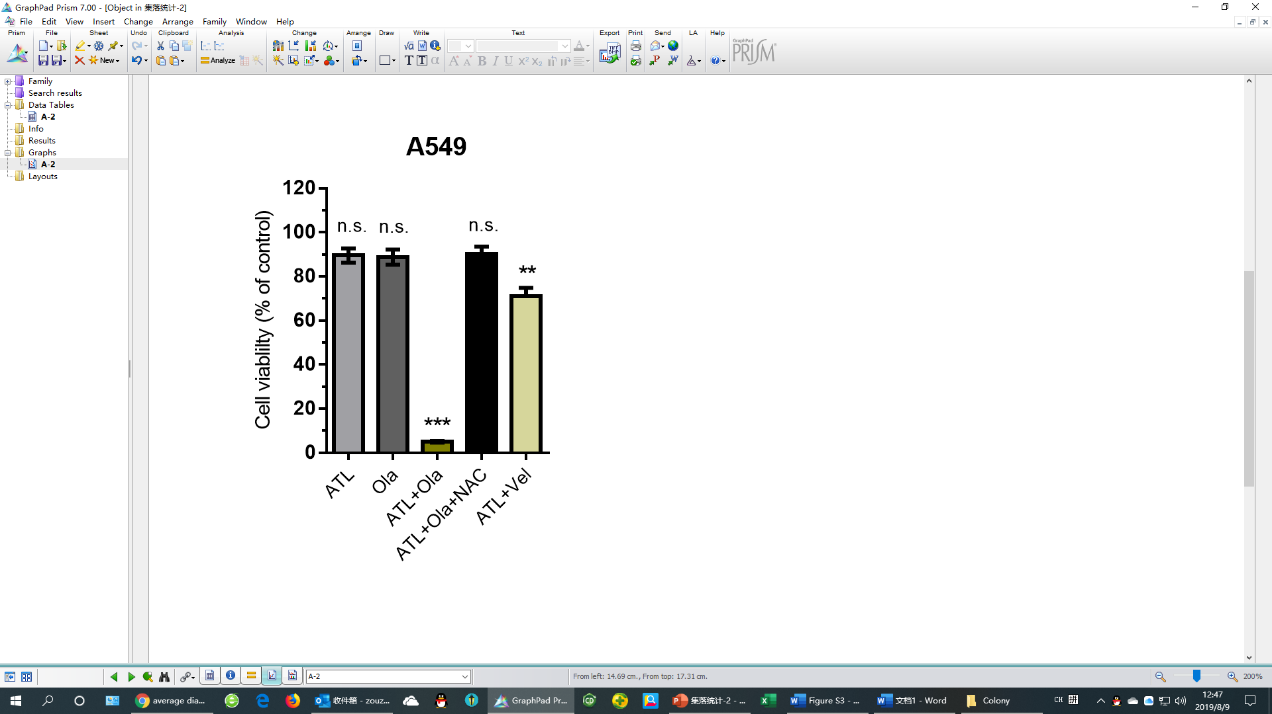


**A549**


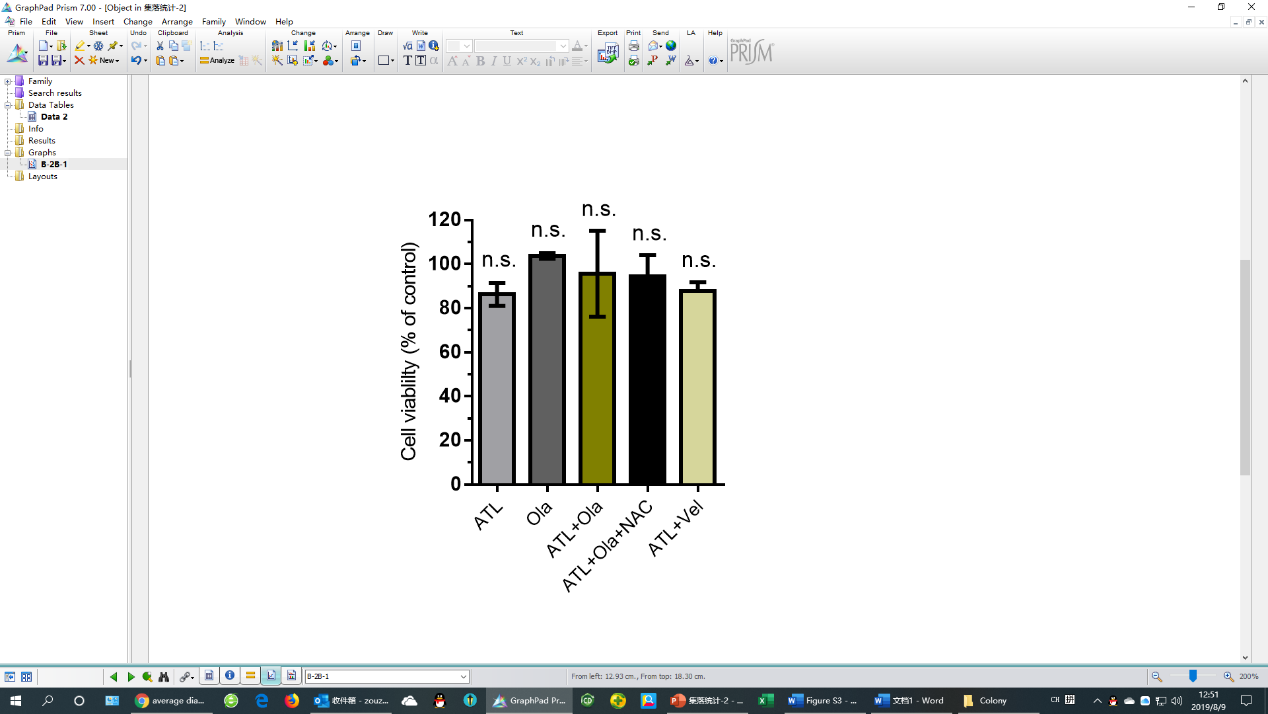


**BEAS-2B**

Supplement: Supplementary file 4 — Supplementary figure 3 [file 41388_2020_1191_MOESM4_ESM.docx]

**Figure S4**

**C**


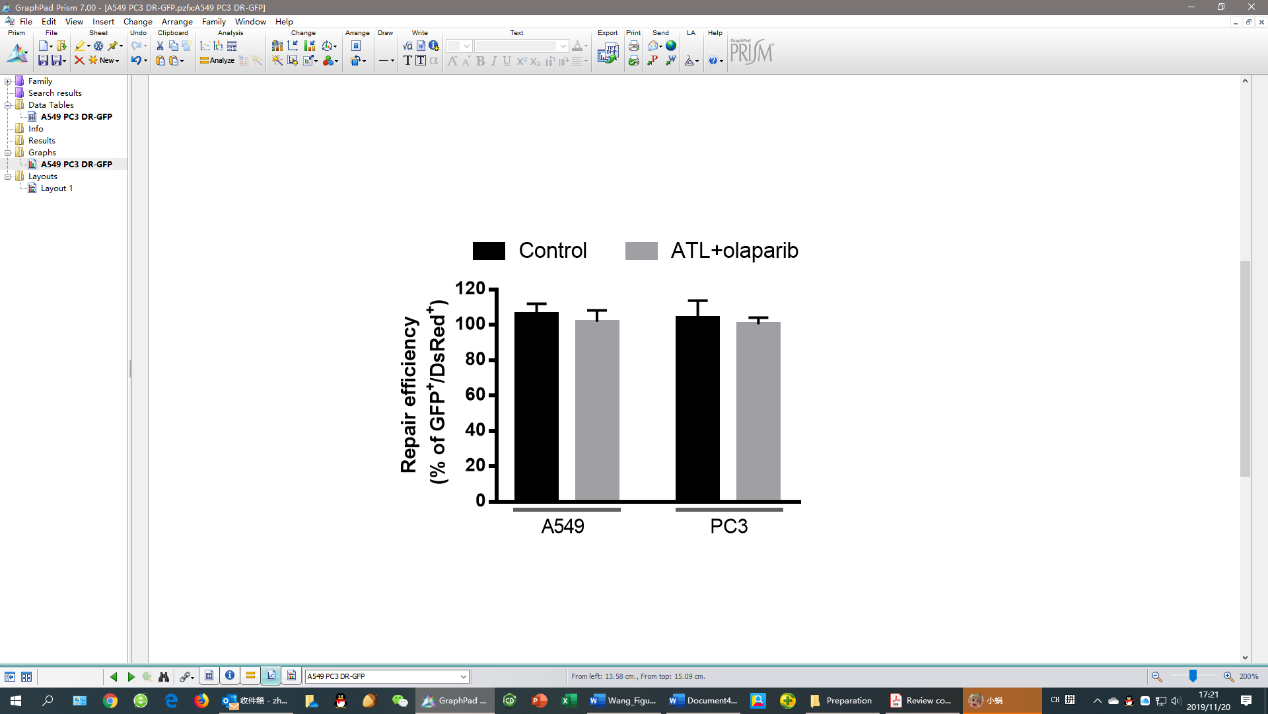

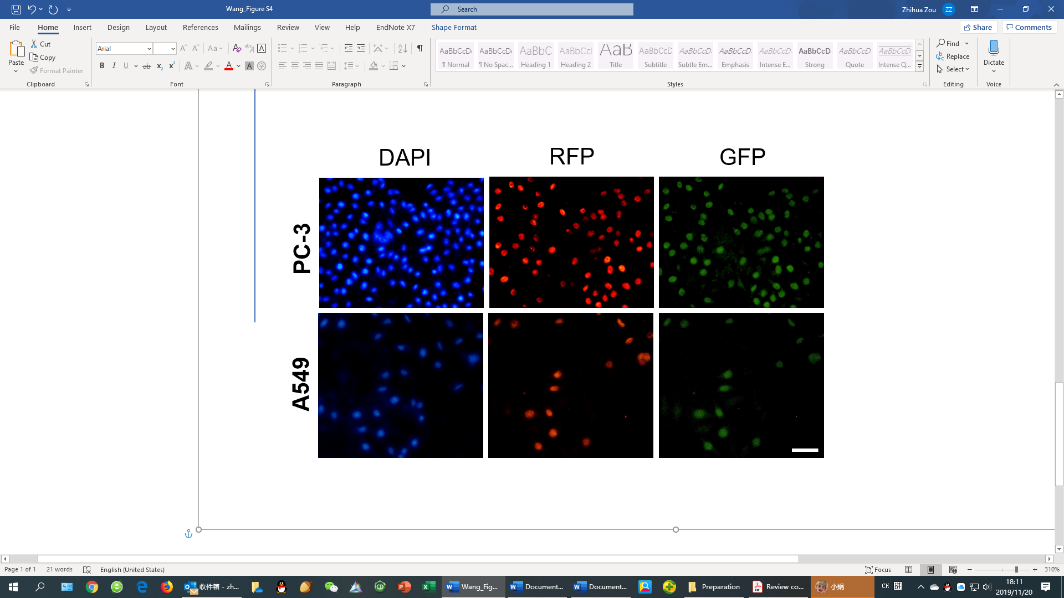


**D**


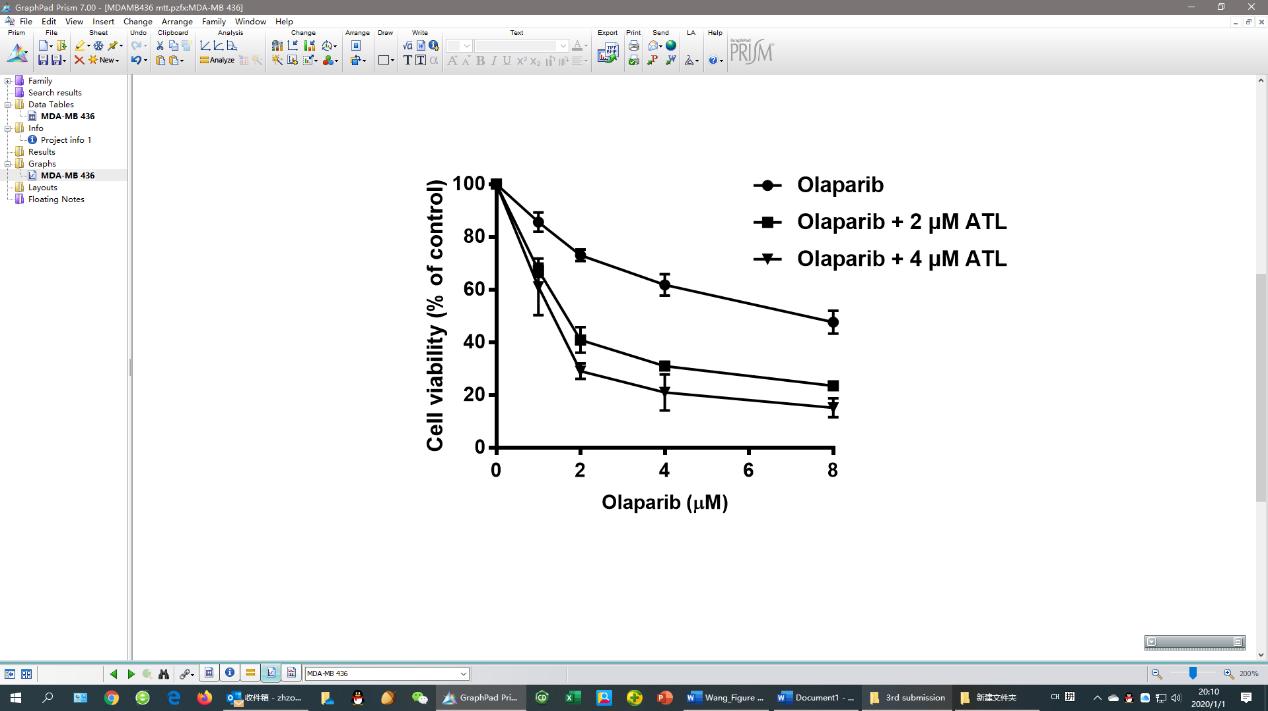


**A**


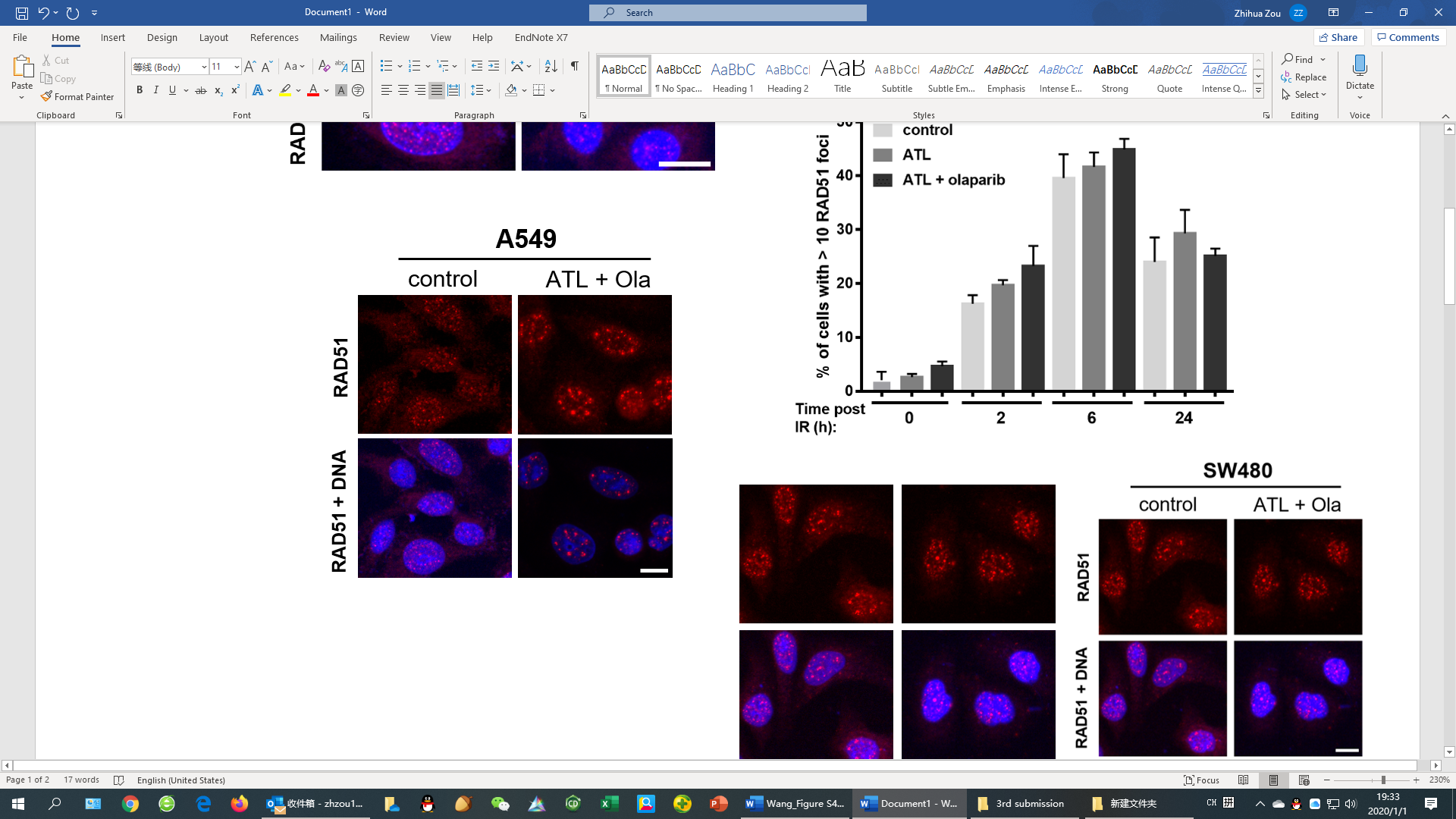

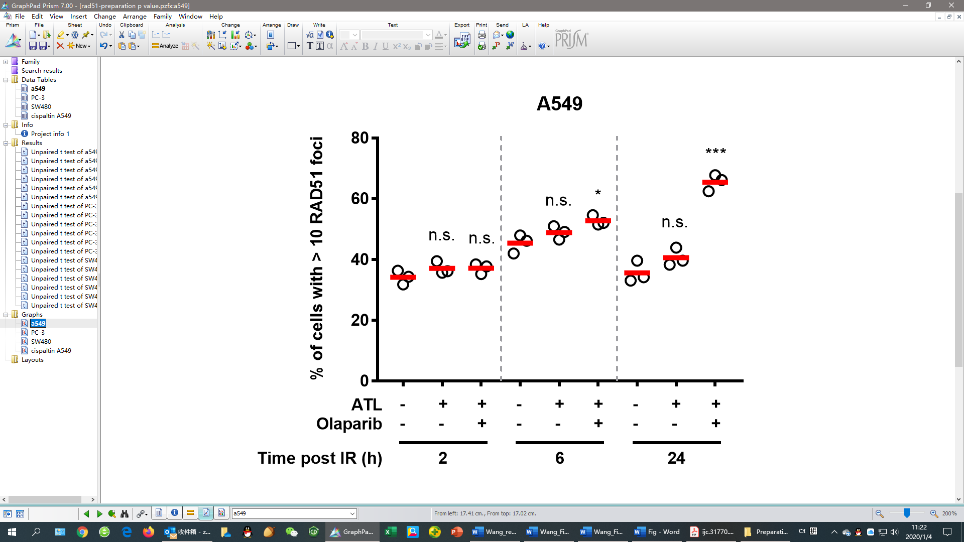

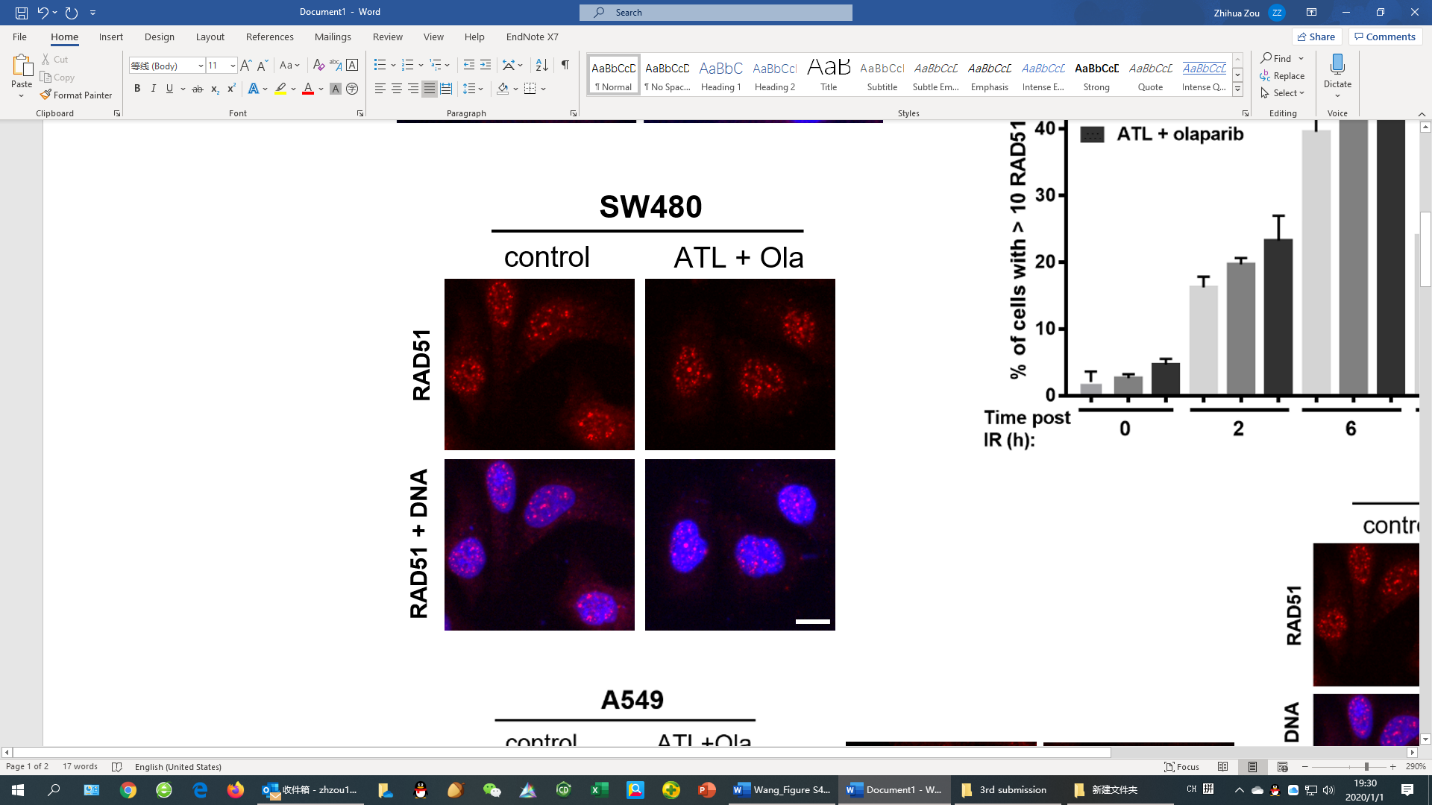

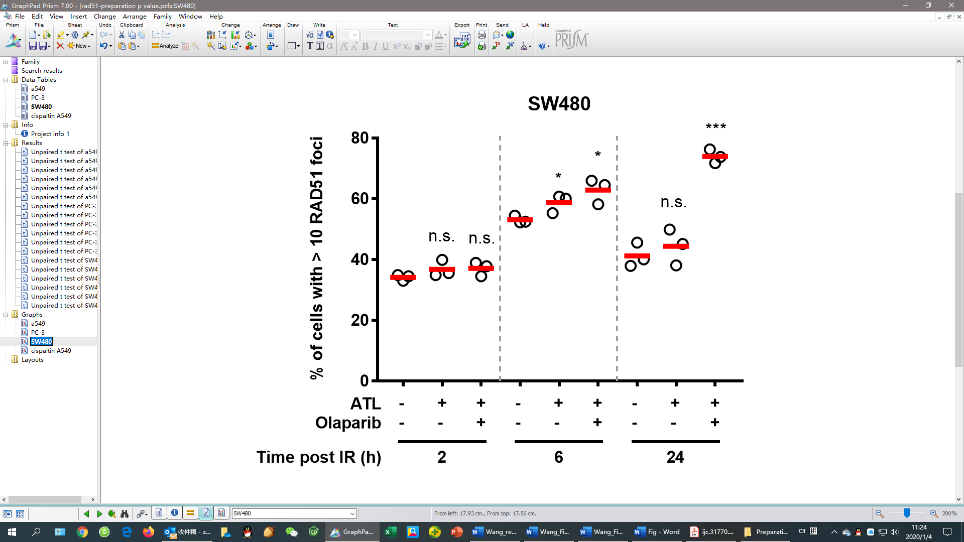


**B**


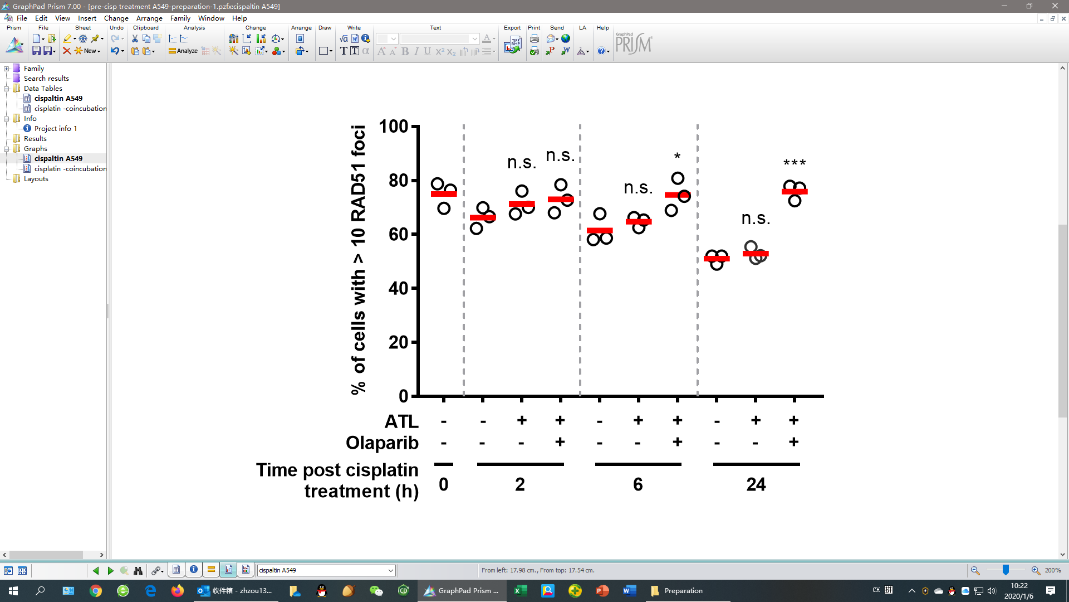

Supplement: Supplementary file 5 — Supplementary figure 4 [file 41388_2020_1191_MOESM5_ESM.docx]

**Figure S6**


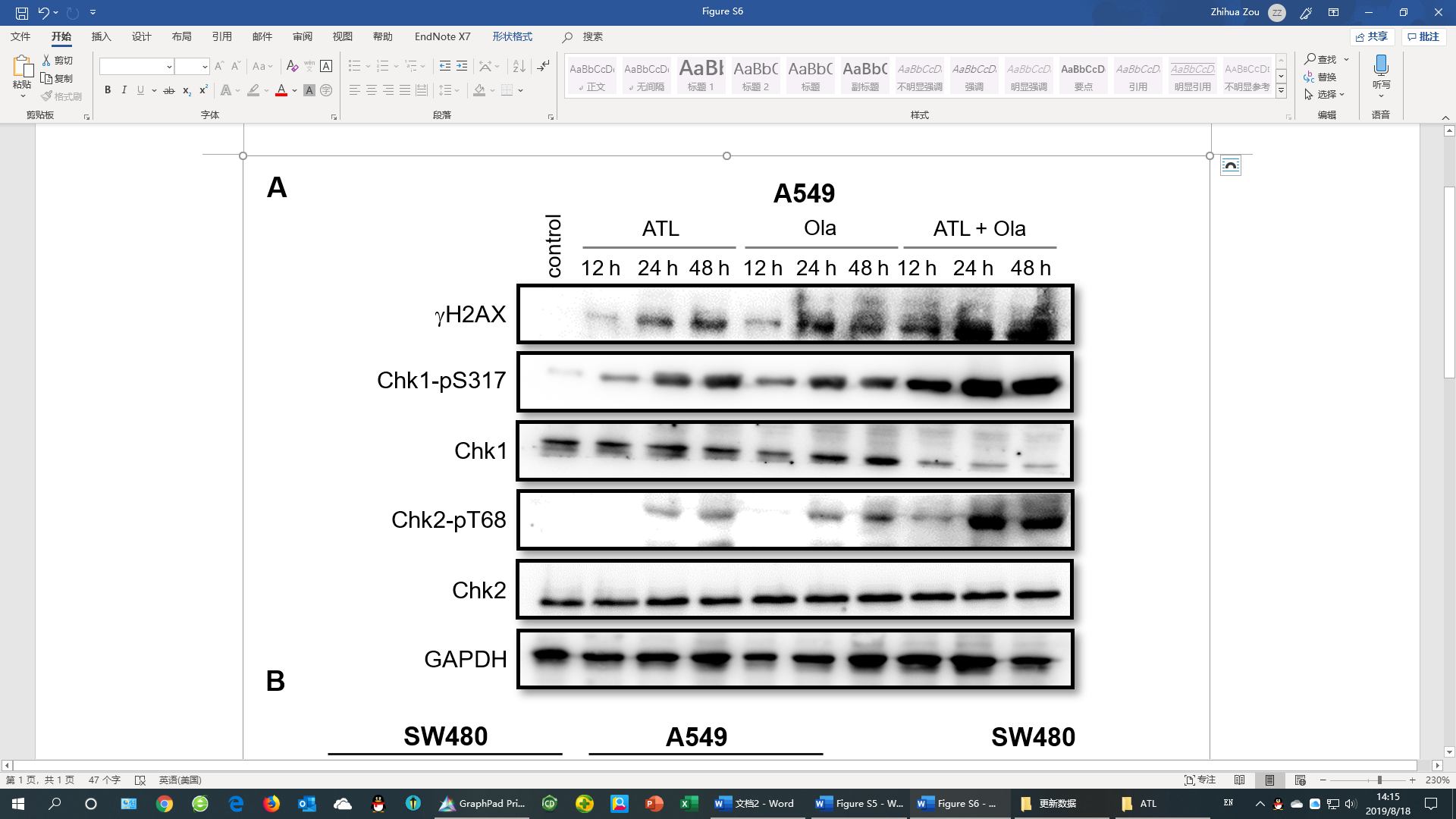


**B**


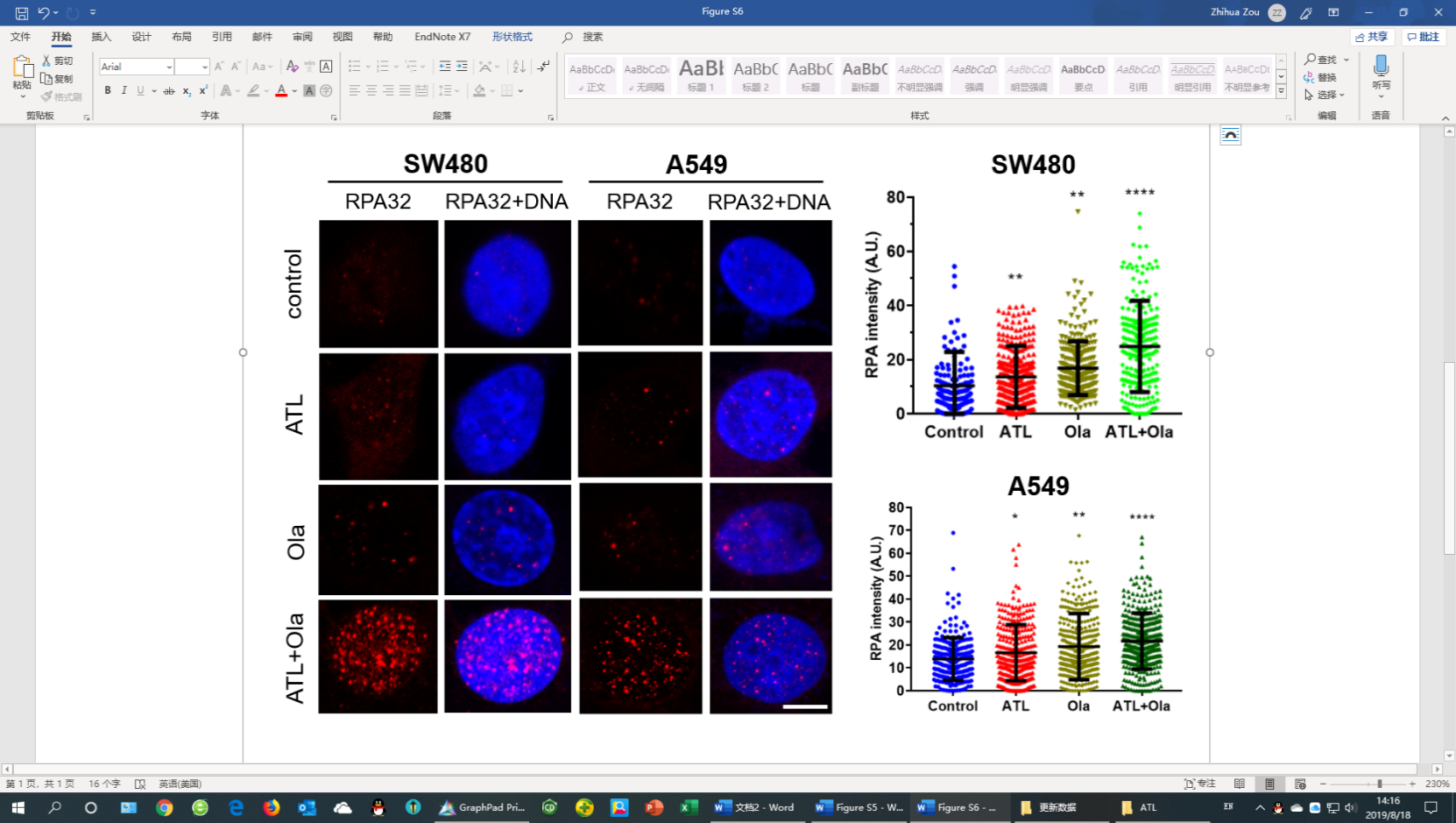


**C**

**A**


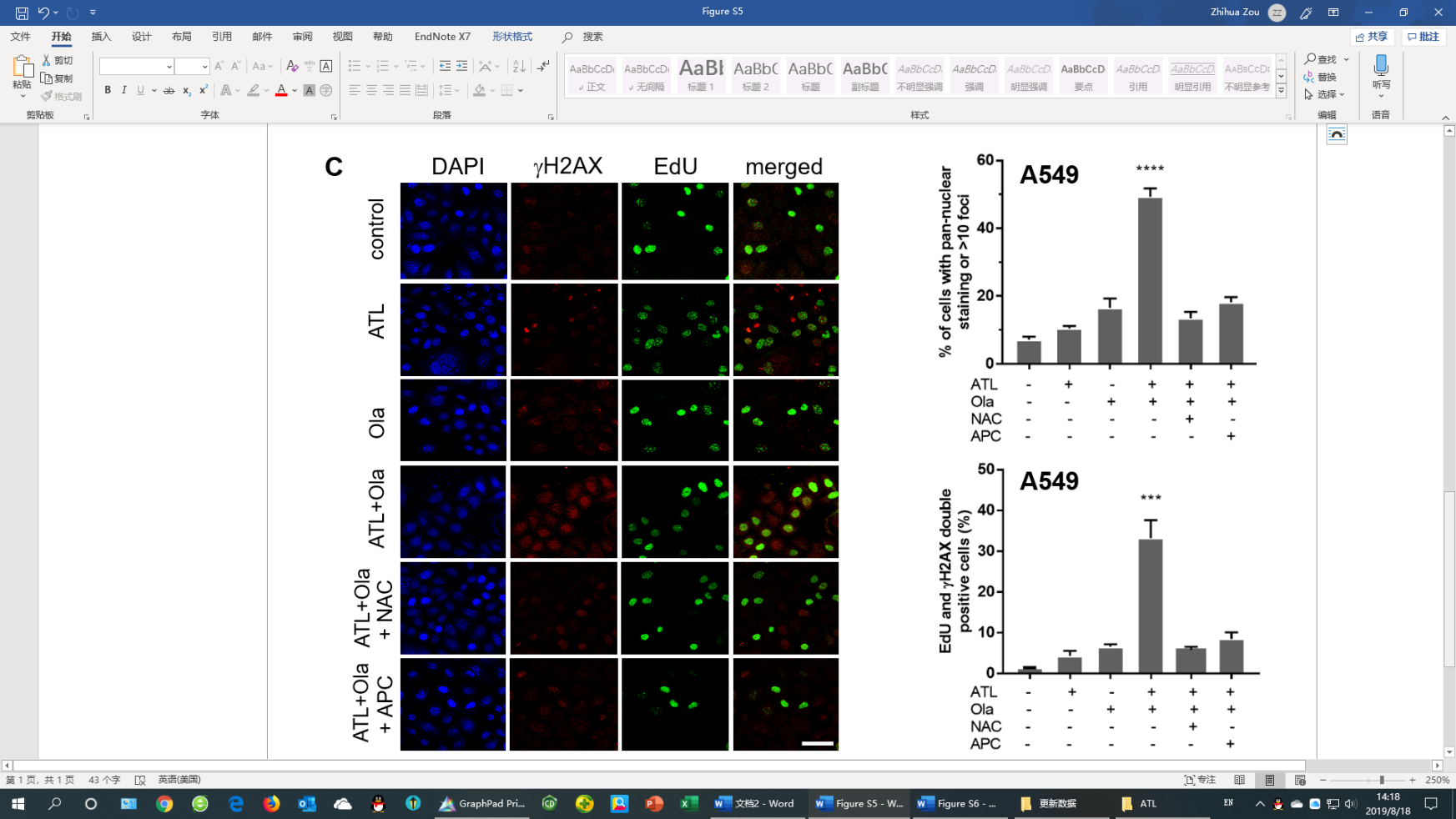

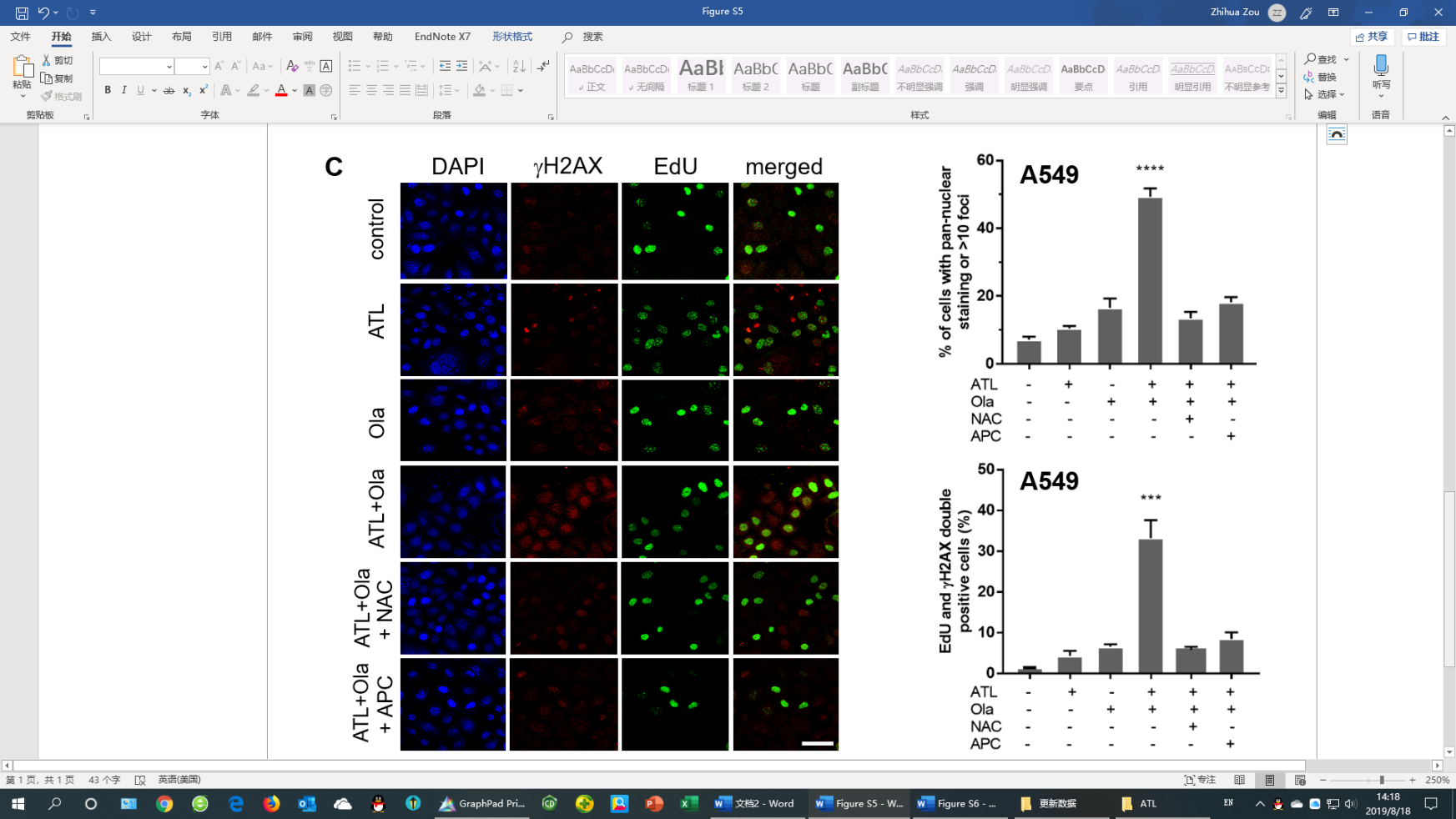

Supplement: Supplementary file 7 — Supplementary figure 6 [file 41388_2020_1191_MOESM7_ESM.docx]

**A**

**B**

53BP1

53BP1 + DNA

ATL

Ola

ATL+Ola


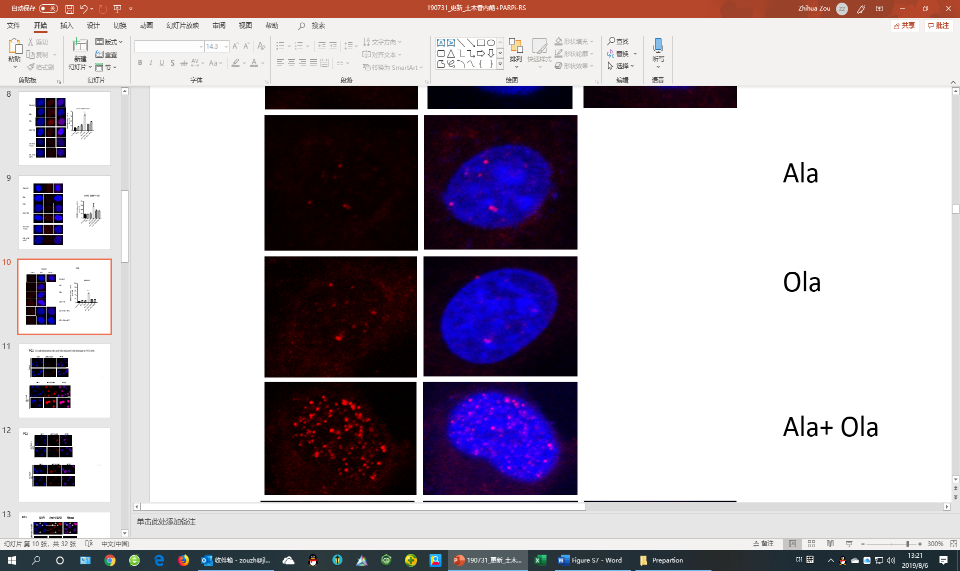


**SW480**

**A549**

53BP1

53BP1 + DNA


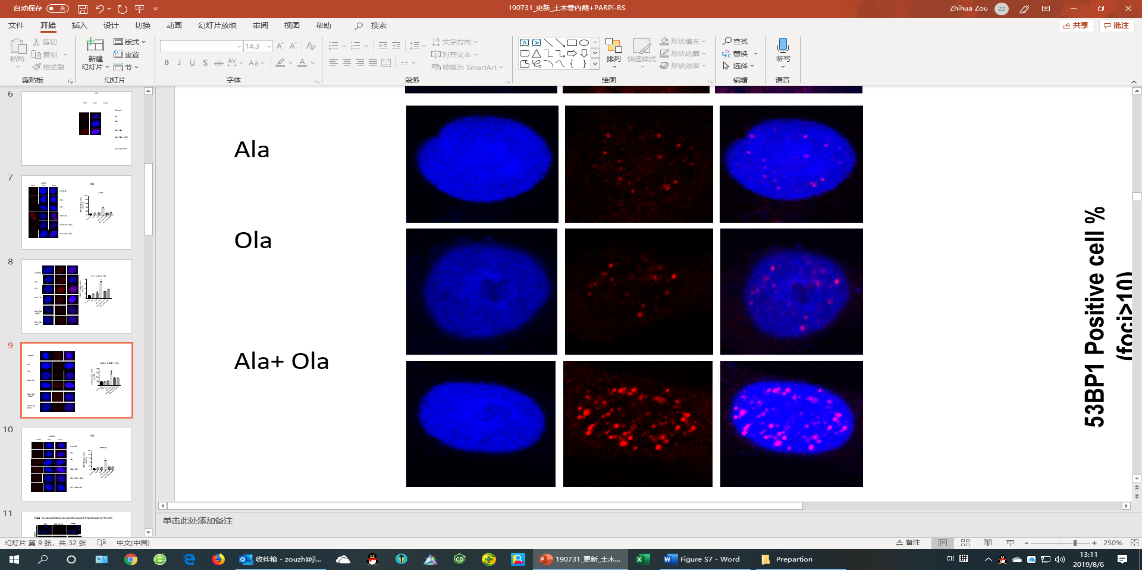


ATL

Ola

ATL+Ola


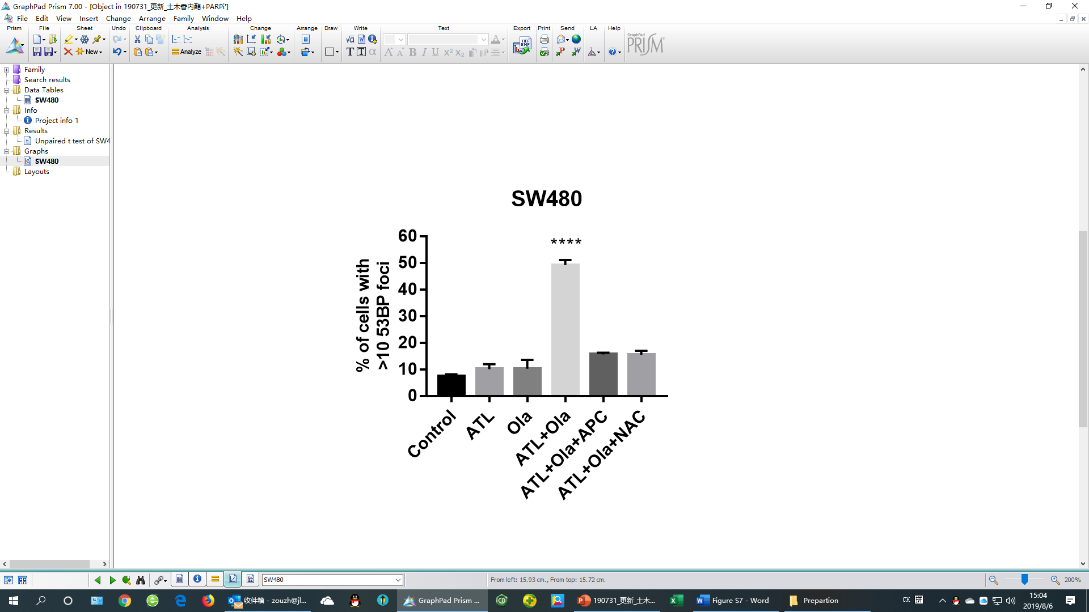

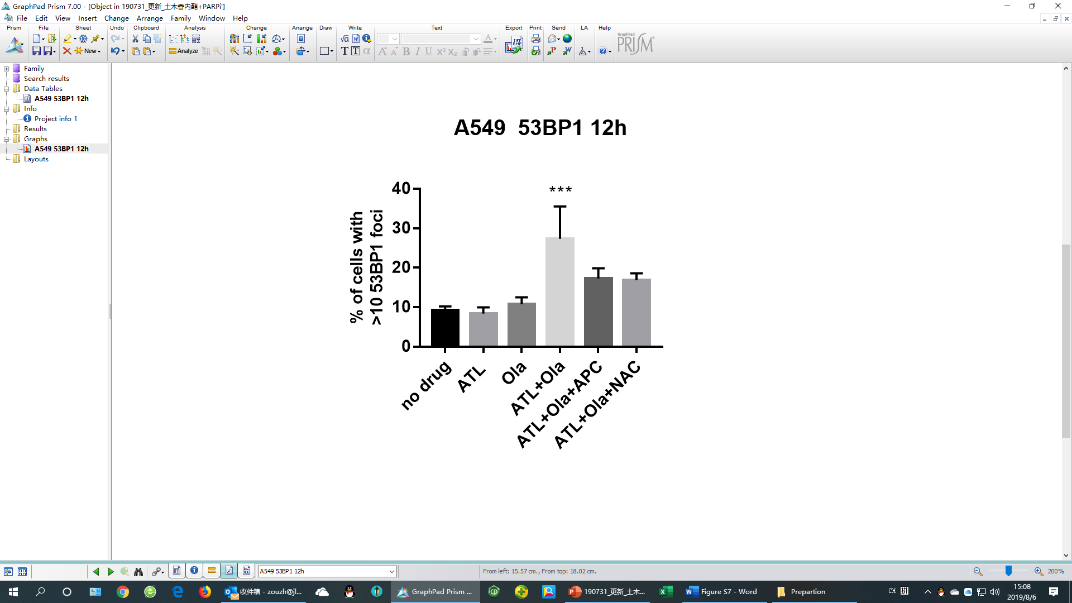


**SW480**

**A549**

**SW480**

**A549**


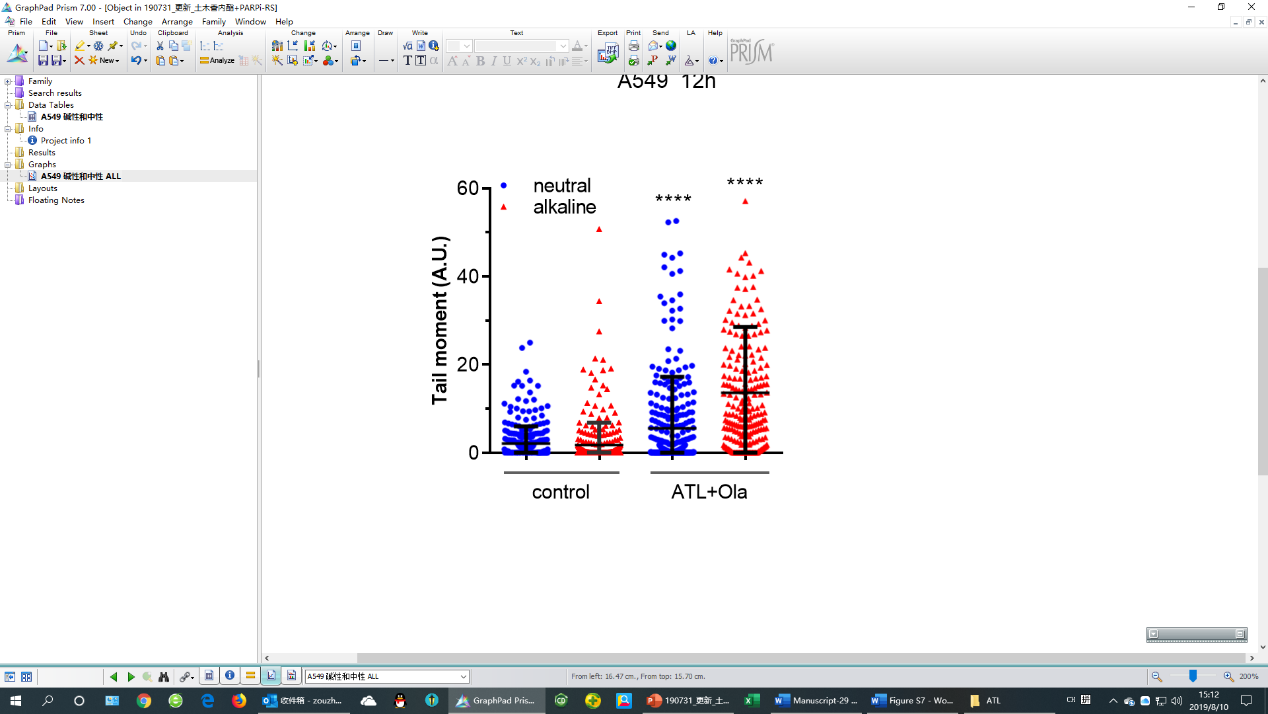

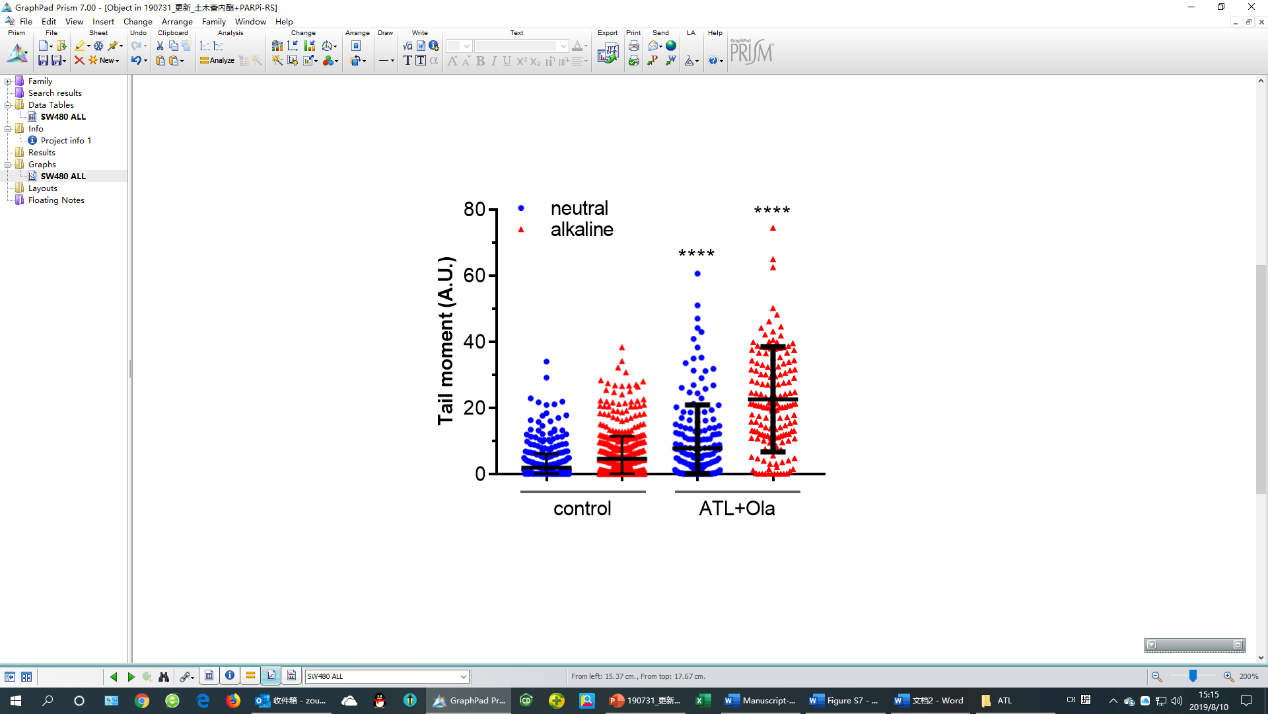


**Figure S7**

Supplement: Supplementary file 8 — Supplementary figure 7 [file 41388_2020_1191_MOESM8_ESM.docx]
