# Supplementary material for: Synergistic lethality between PARP-trapping and alantolactone-induced oxidative DNA damage in homologous recombination-proficient cancer cells
Source: Oncogene. 2020 Feb 6;39(14):2905–20. doi: 10.1038/s41388-020-1191-x (PMC7118026; doi:10.1038/s41388-020-1191-x)
Supplement: Supplementary file 6 — Supplementary figure 5 [file 41388_2020_1191_MOESM6_ESM.docx]

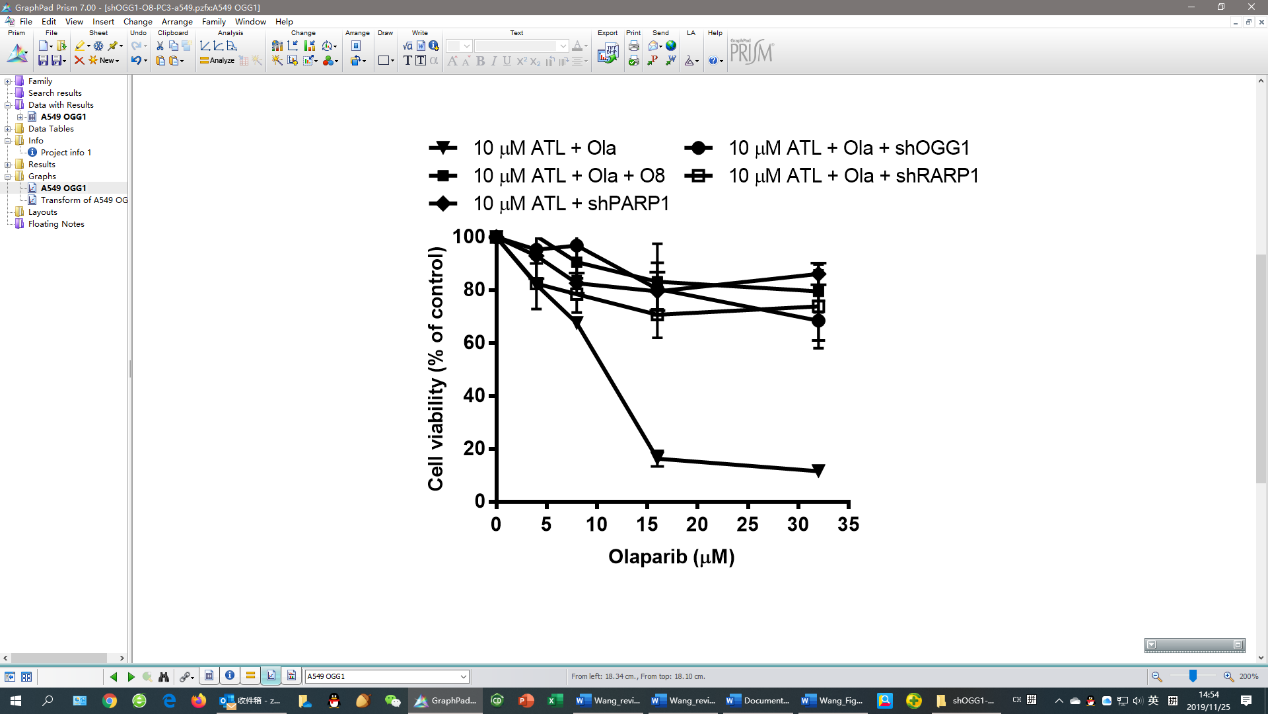


PARP1

GAPDH

Histone H3

cytoplasmic fraction

chromatin fraction

PAR

ATL

ATL+Vel

Ola

ATL+Ola

No drug

ATL

Ola

ATL+Ola

Vel

Vel

ATL+Vel


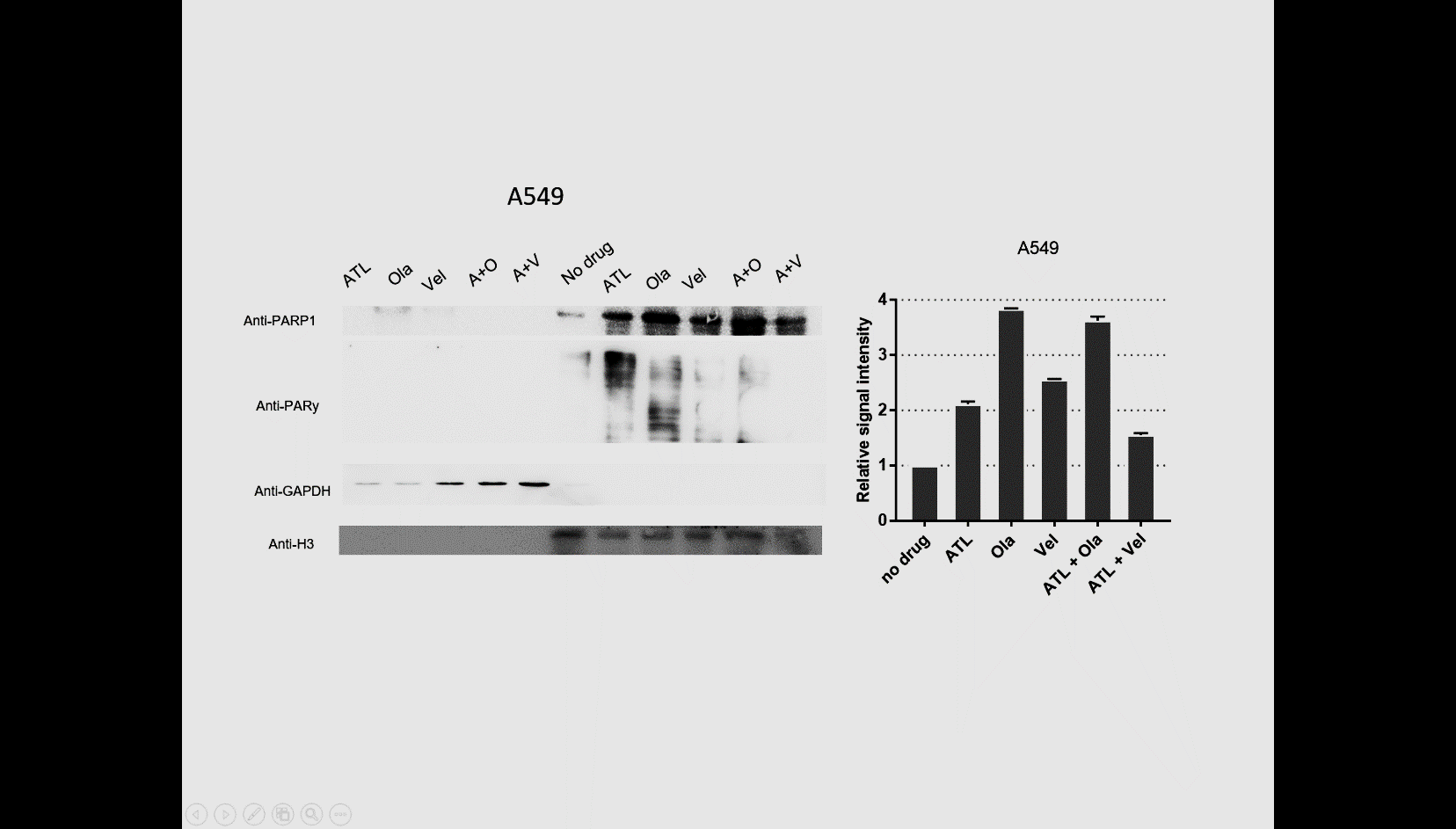

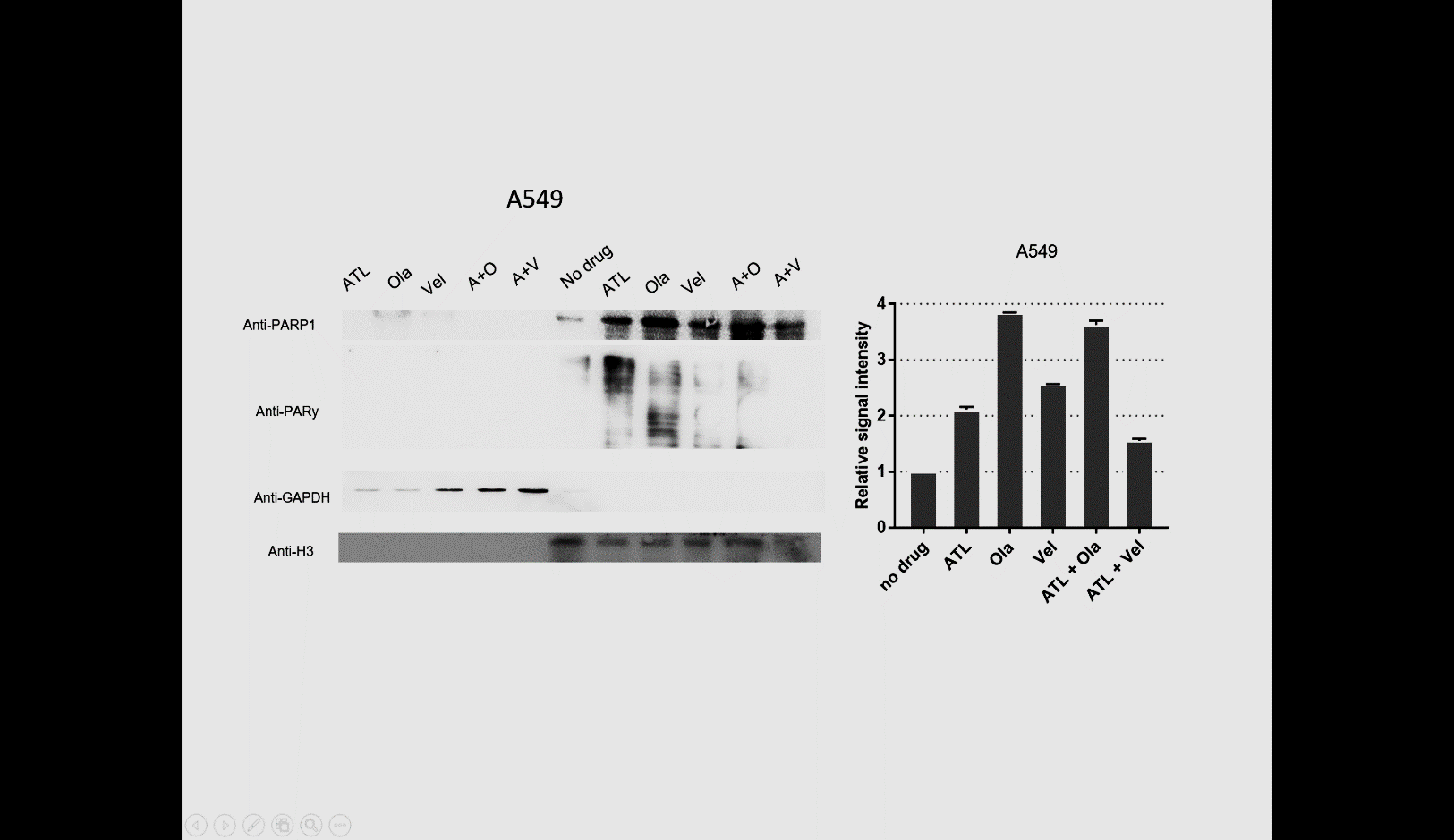

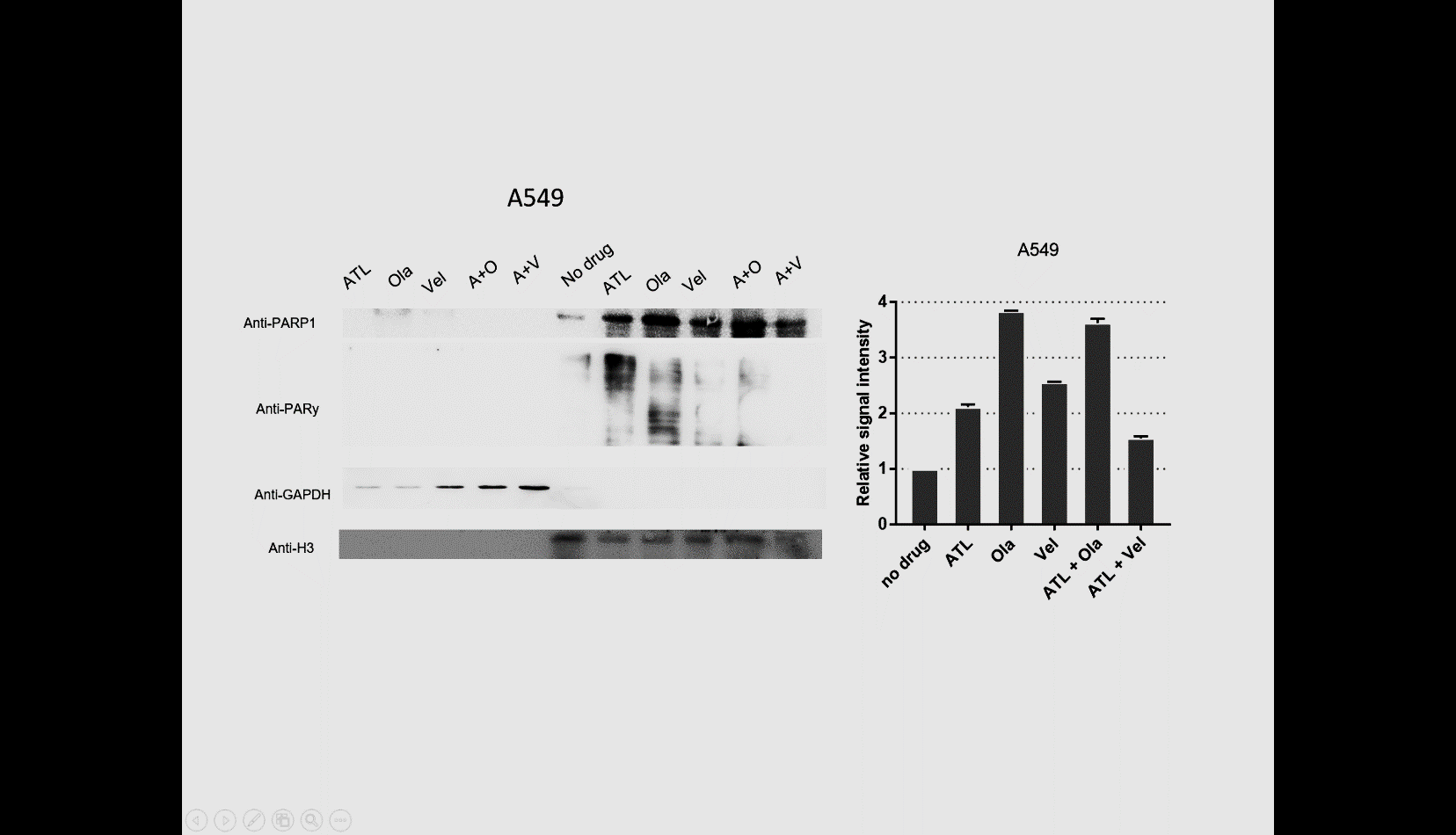

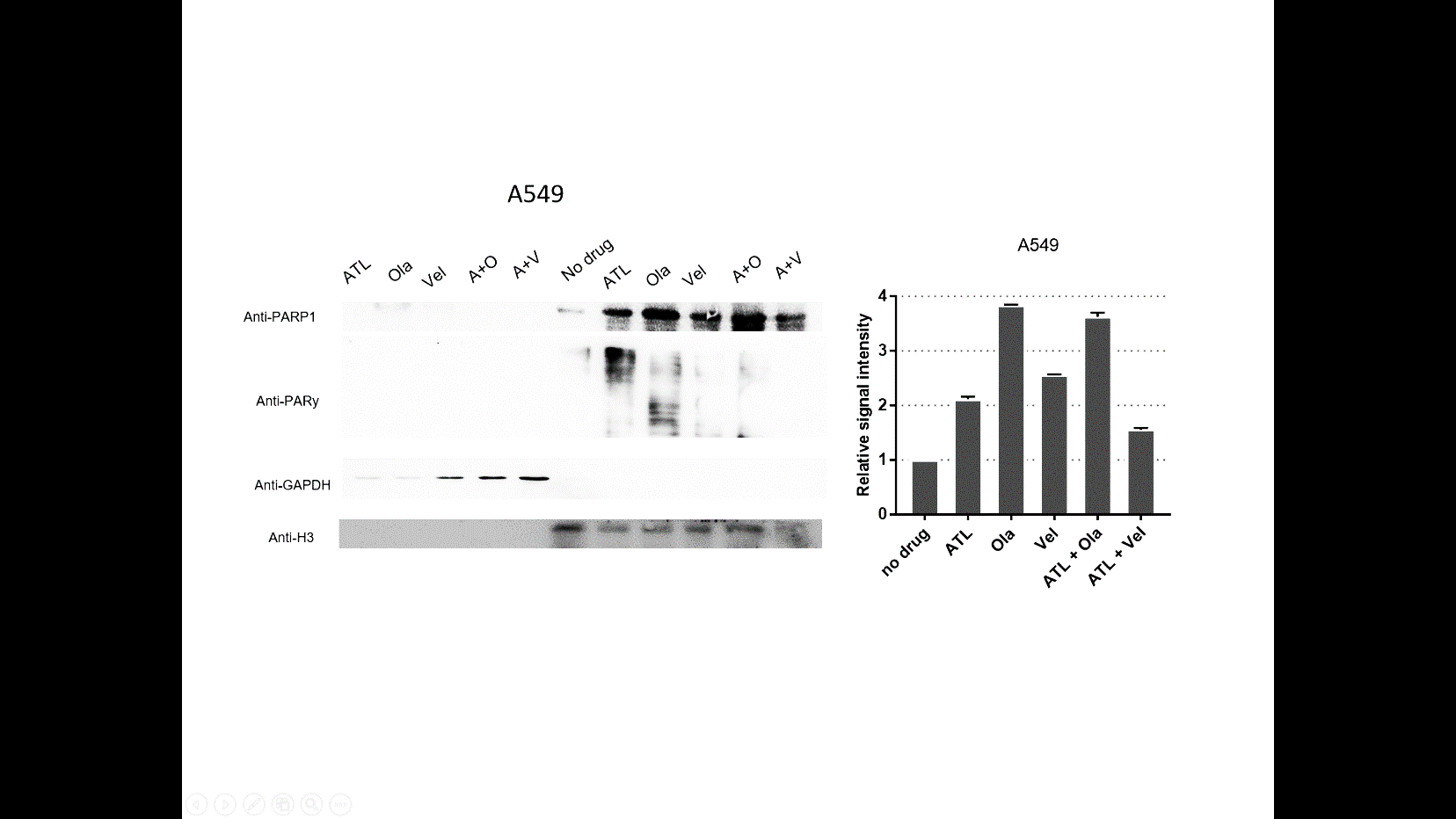


**D**

**A549**


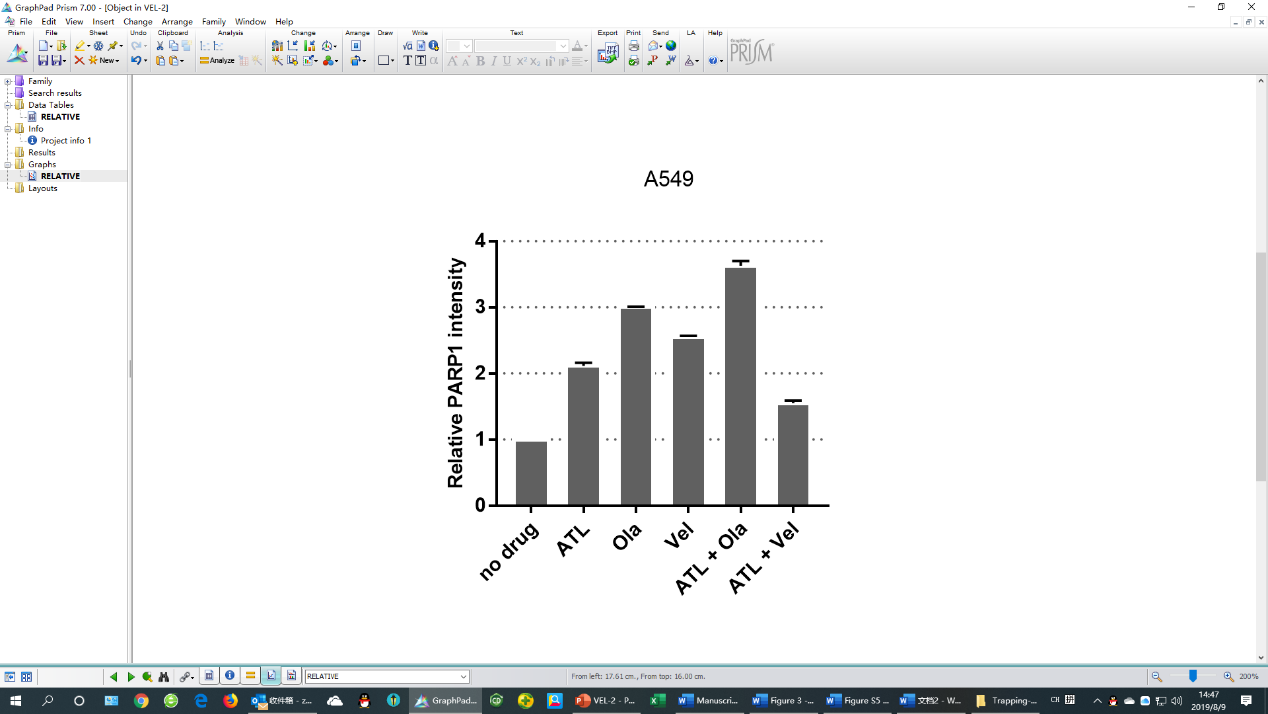

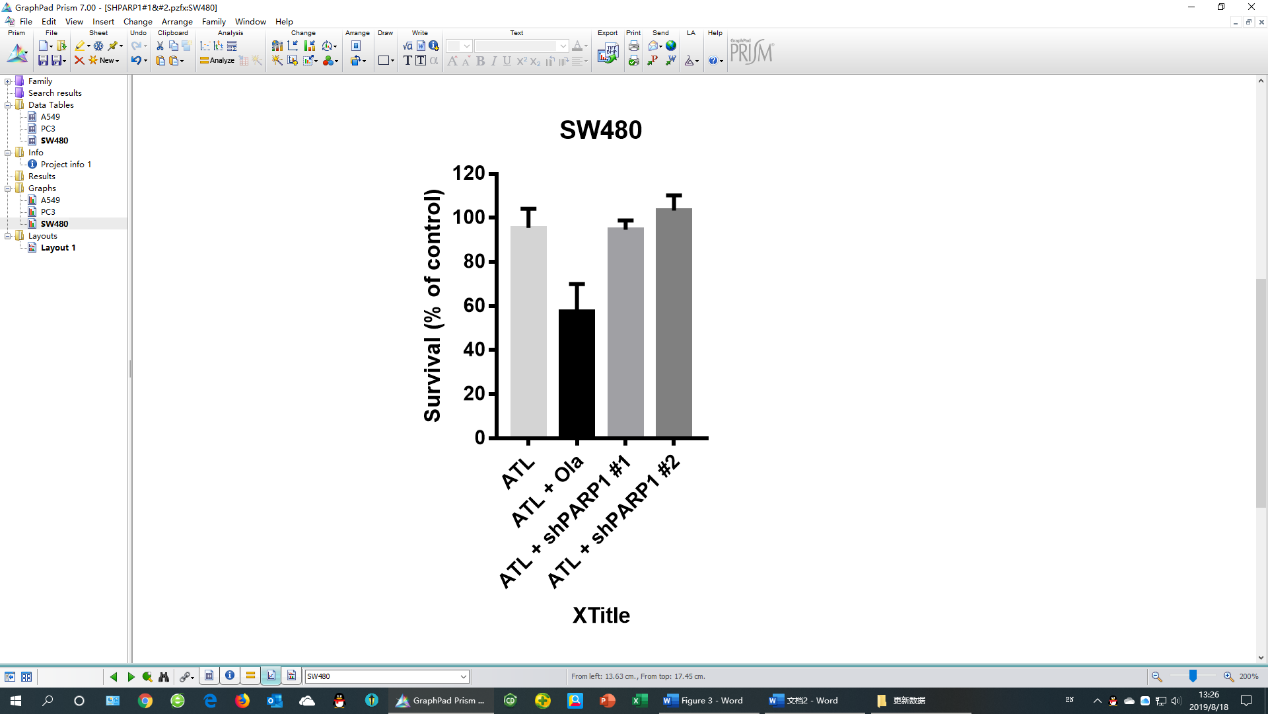

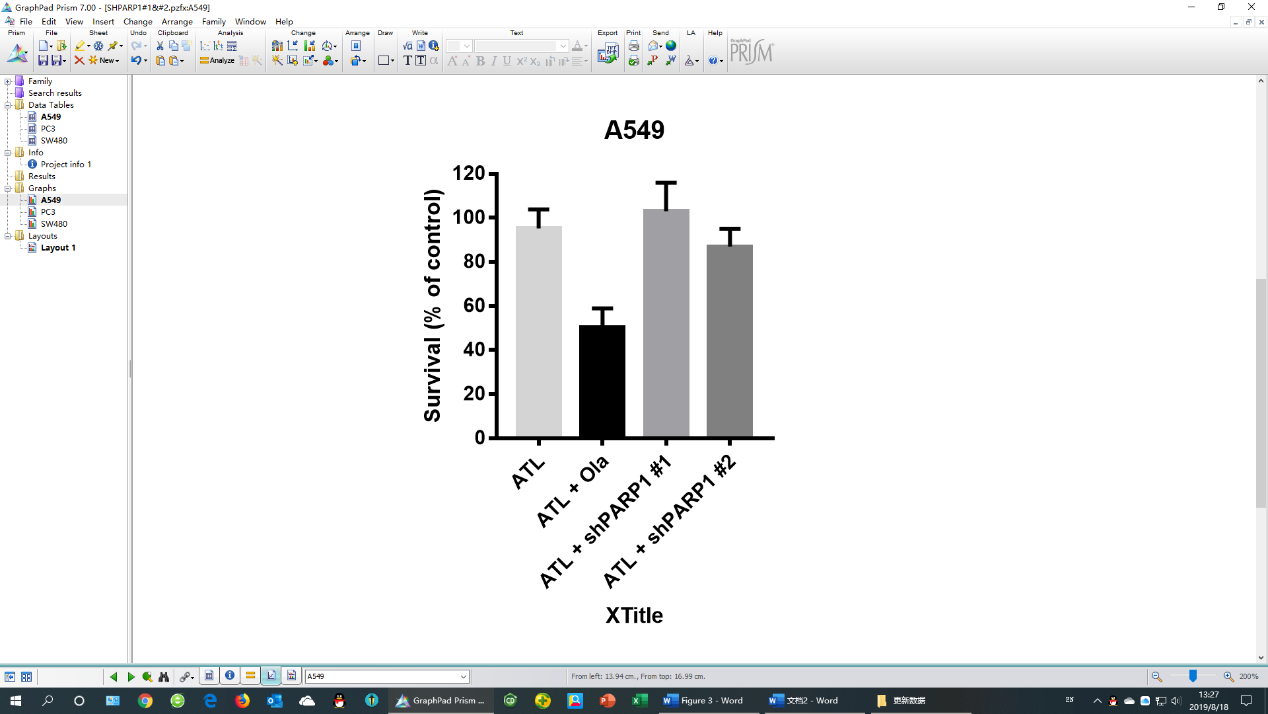


**C**

**A**


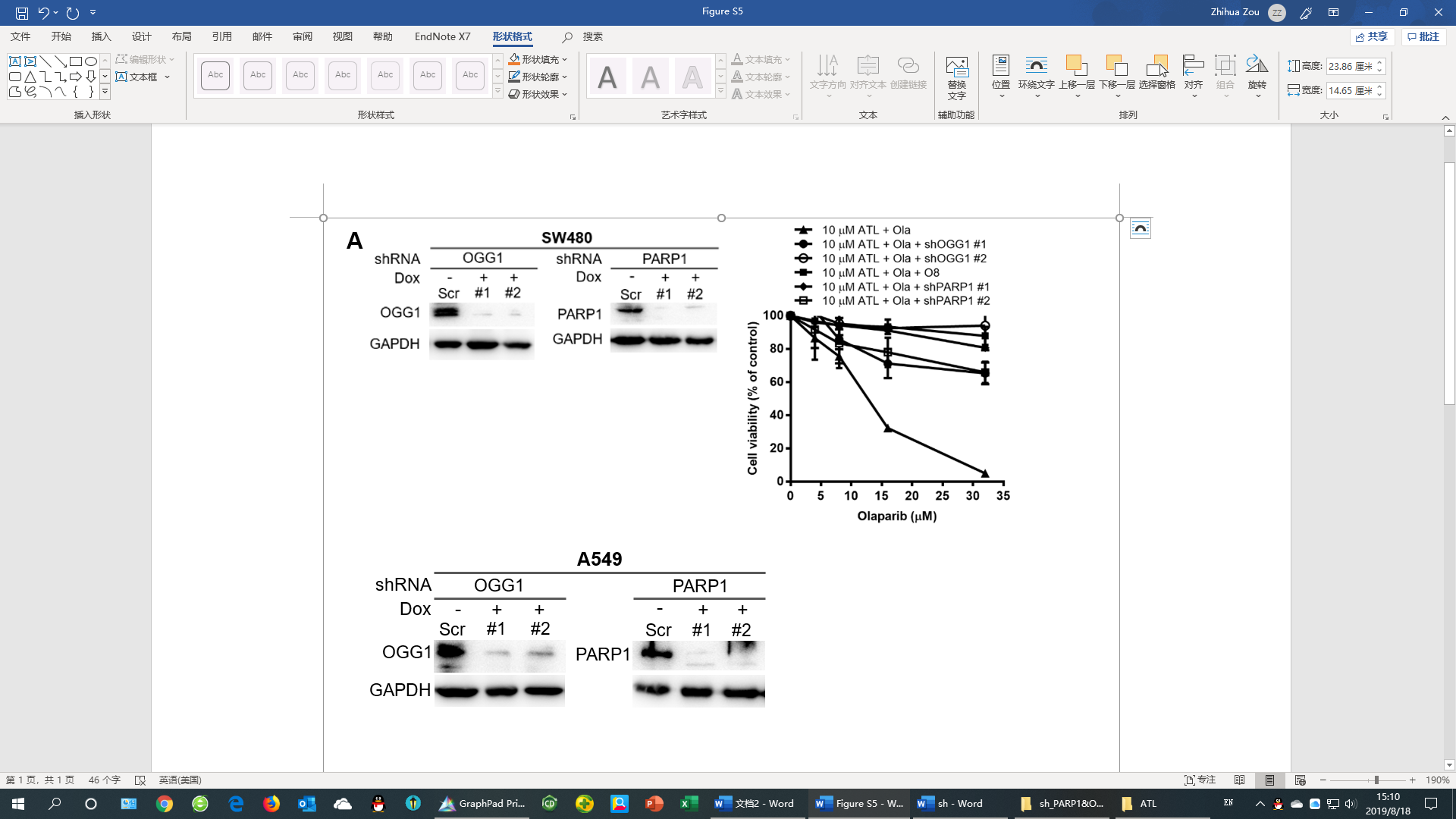

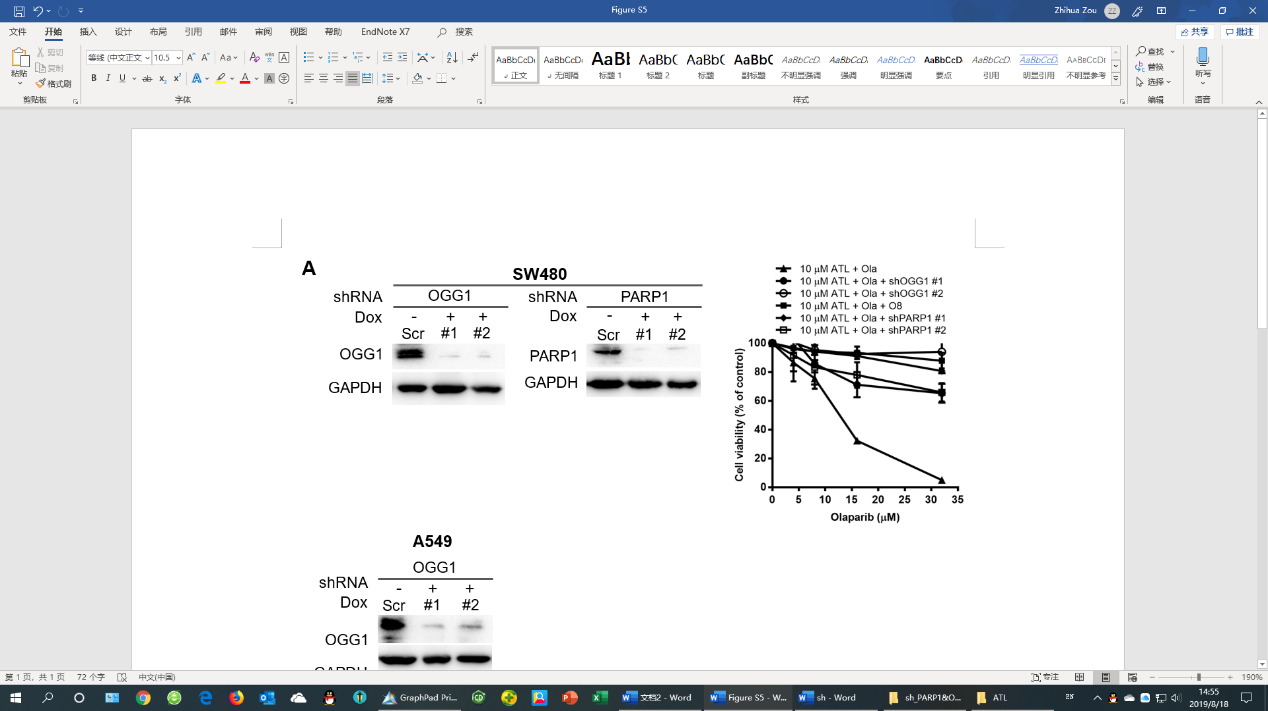

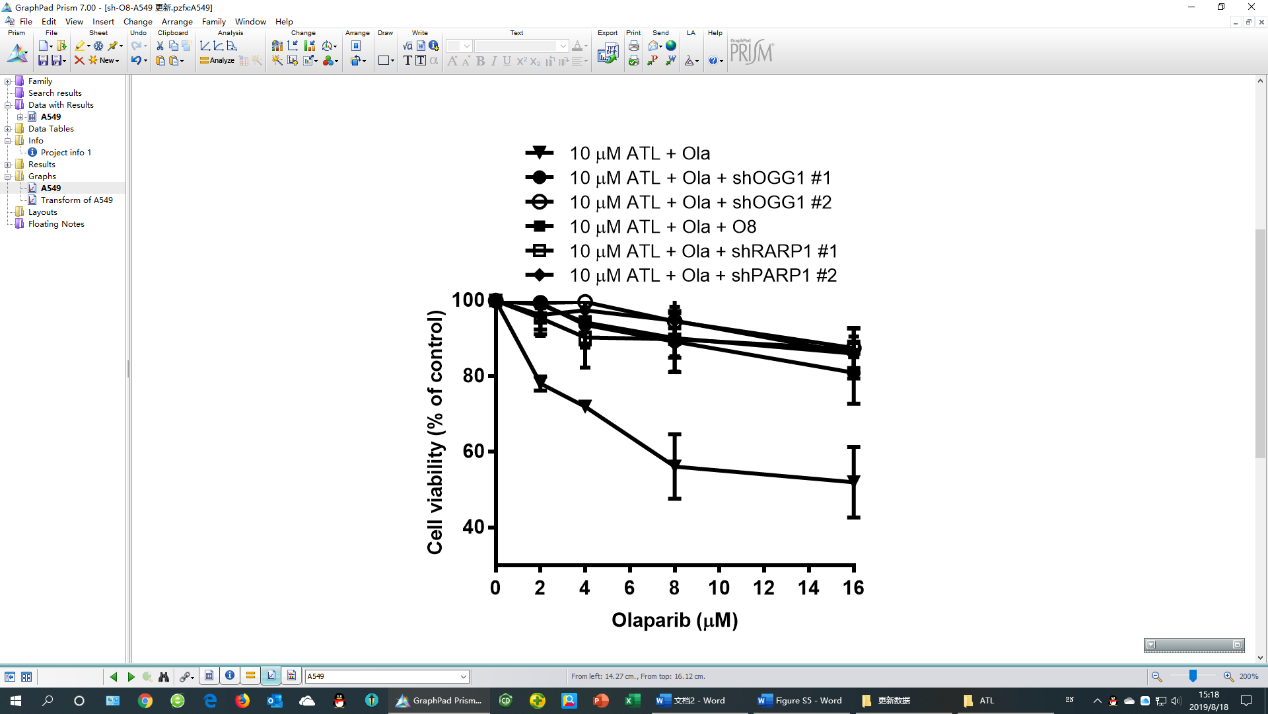

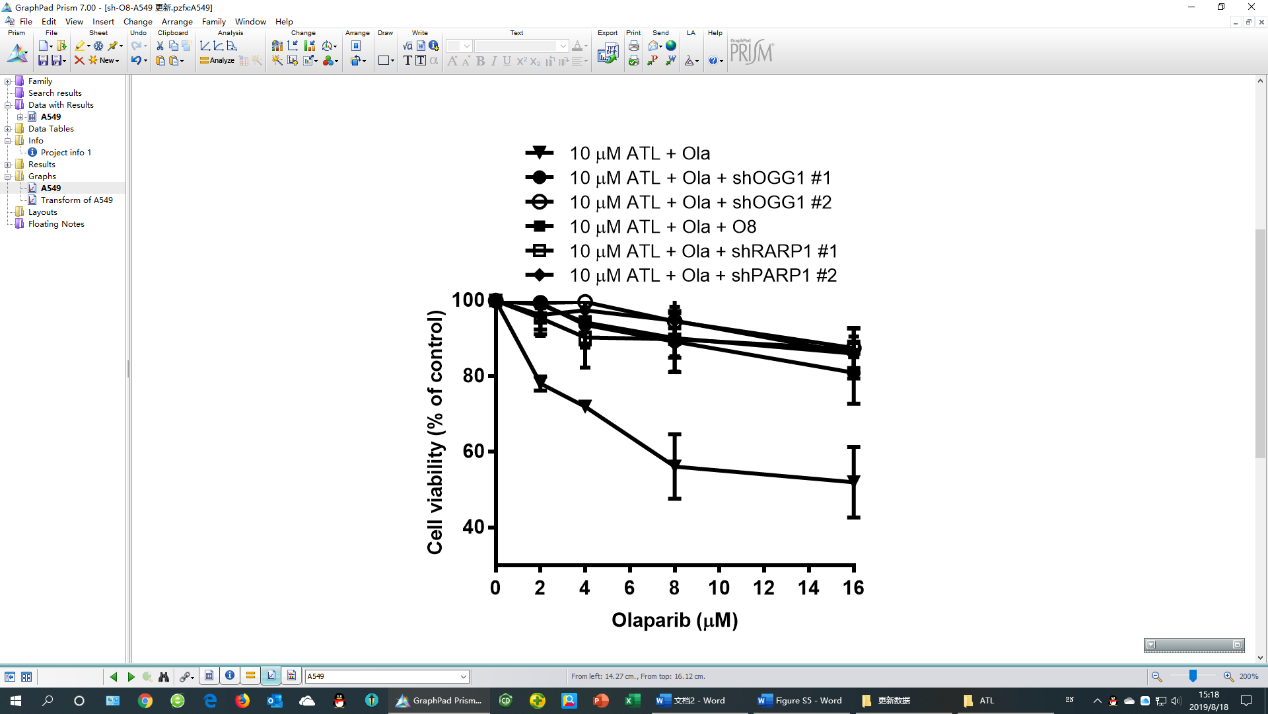

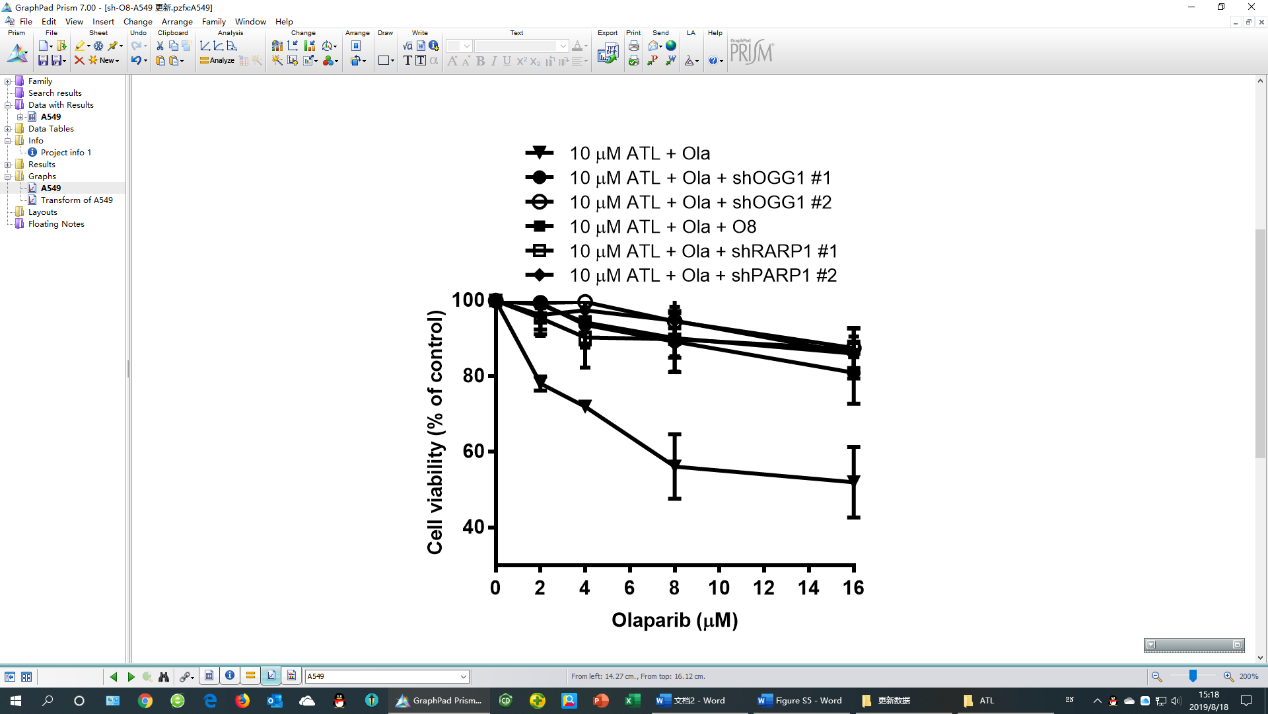

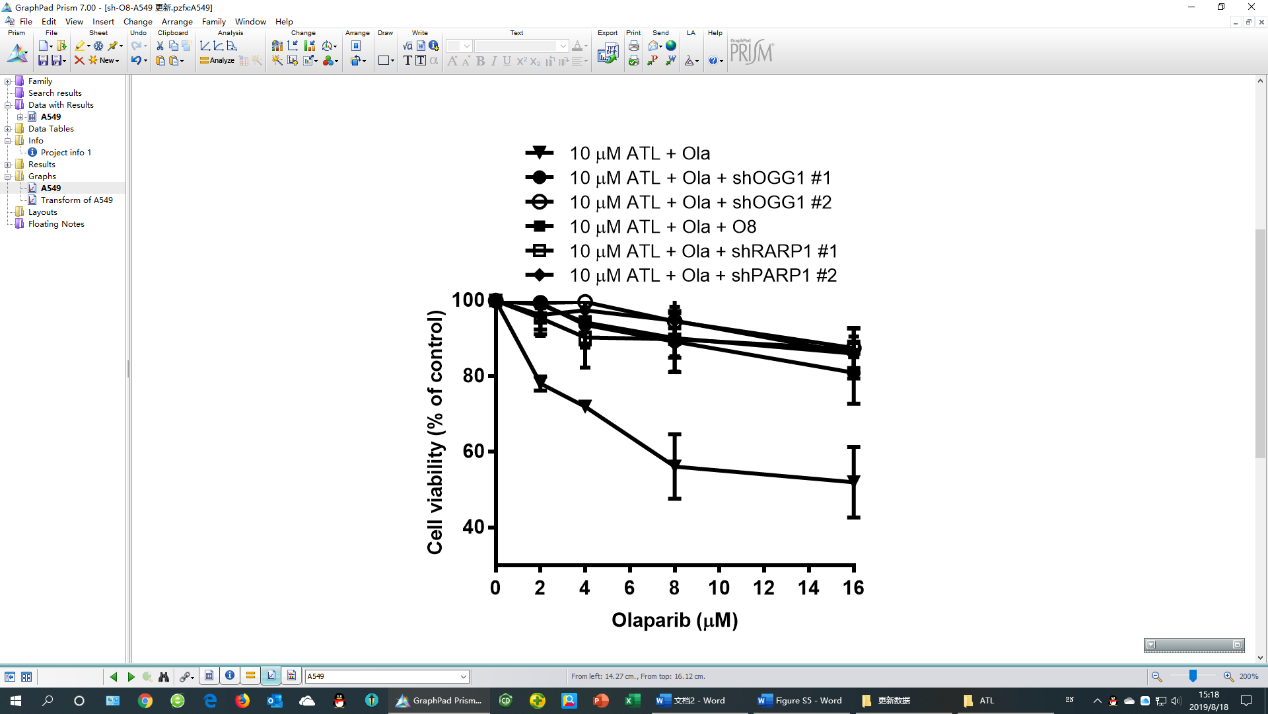

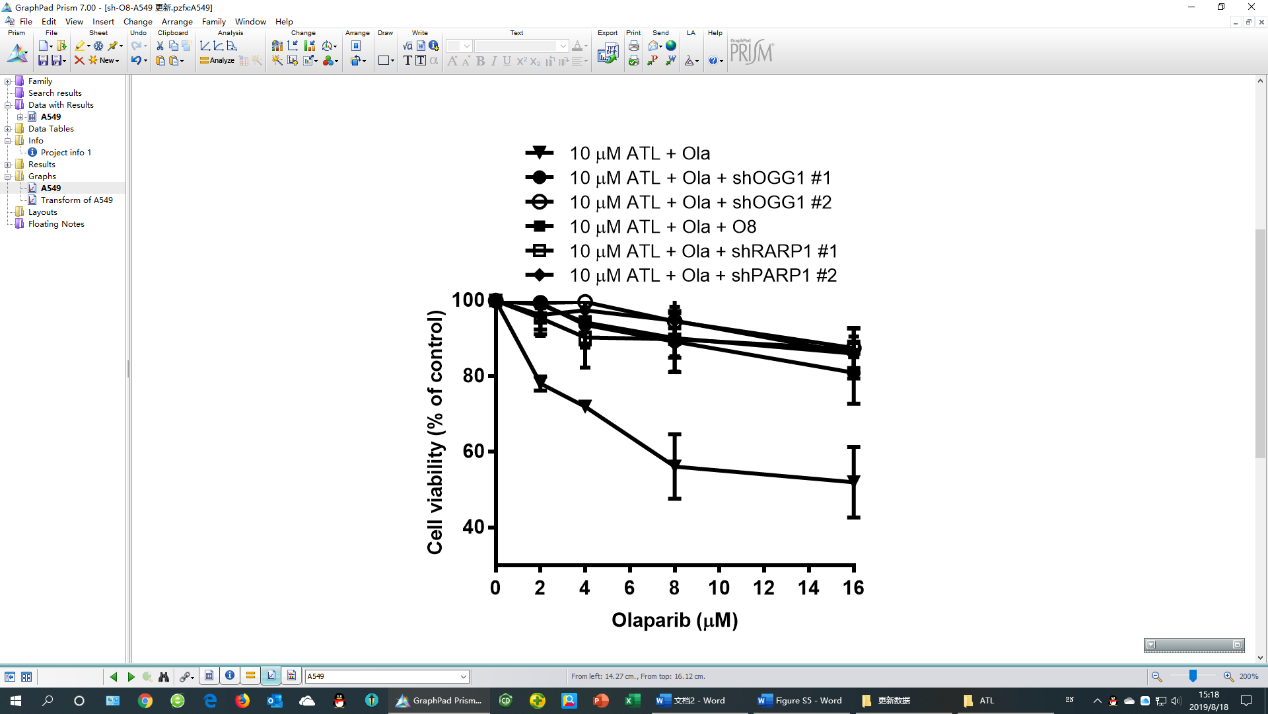


**B**

**A549**

**SW480**


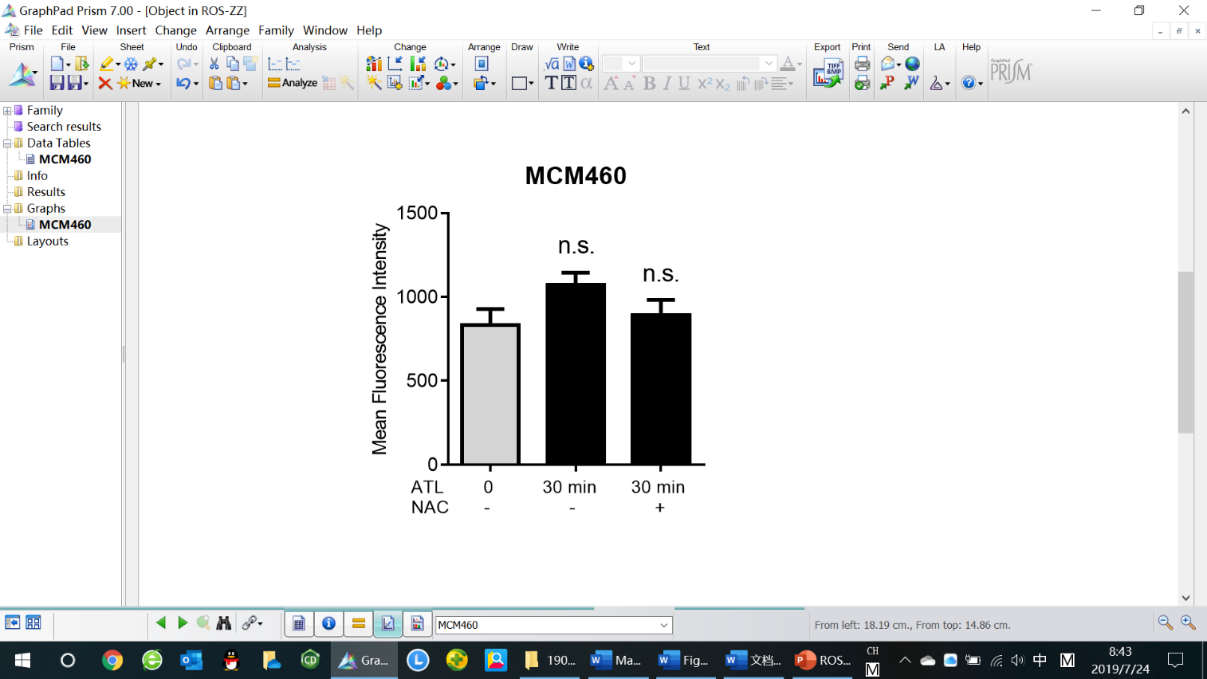

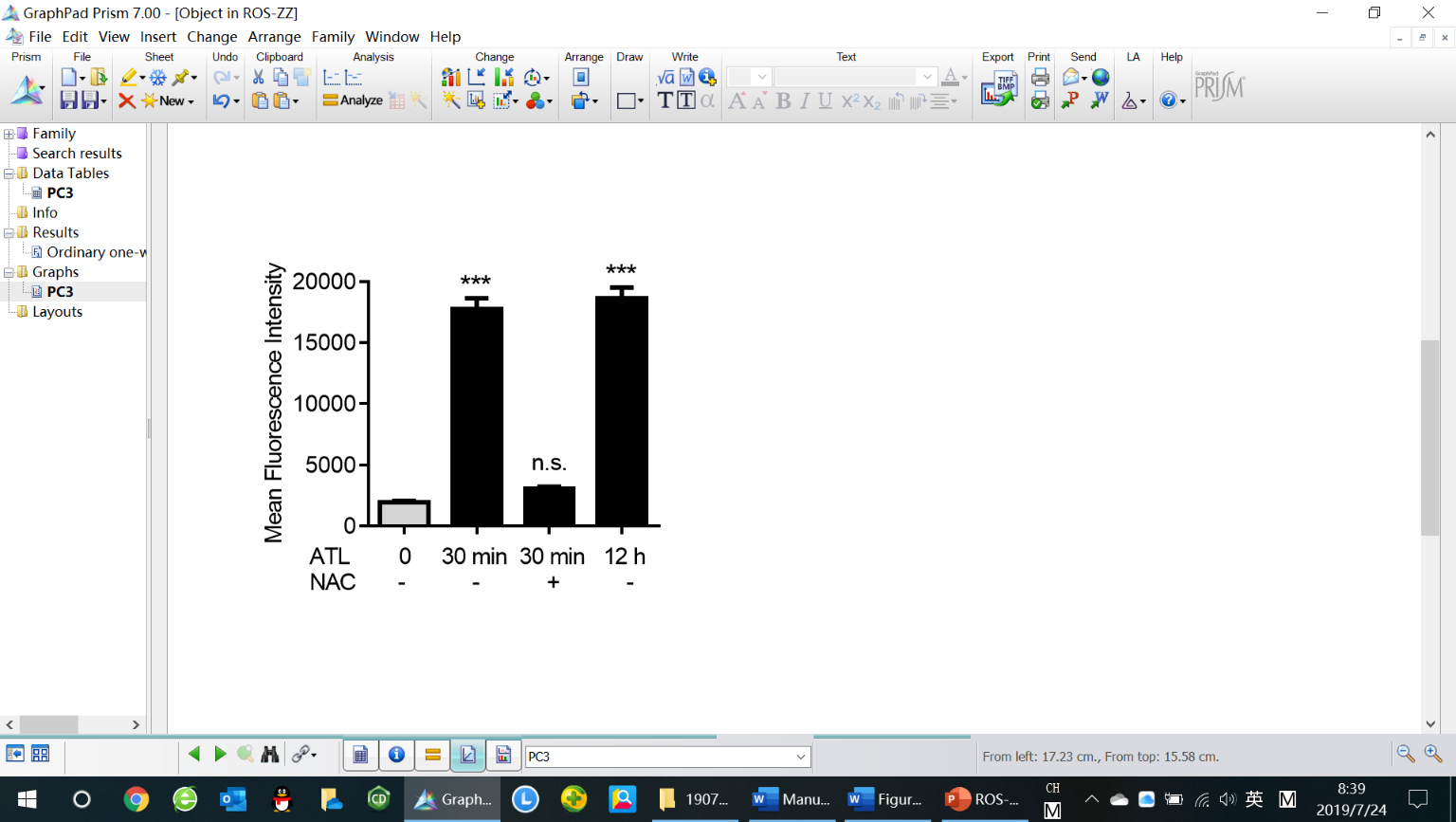

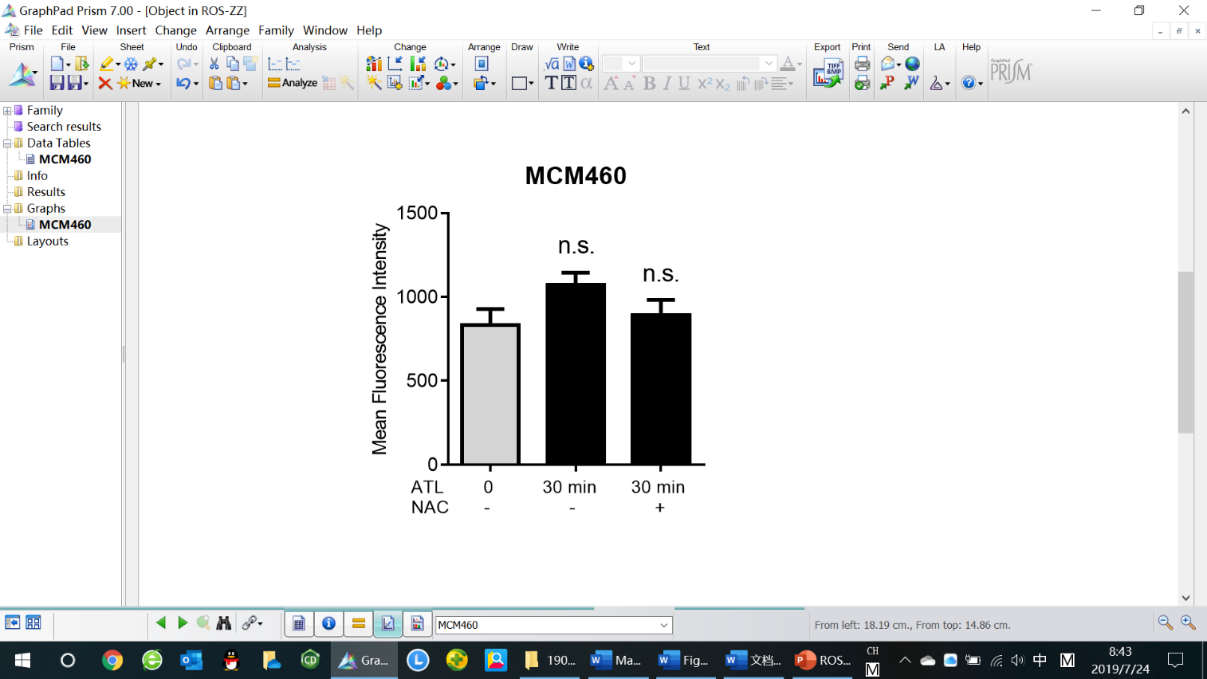

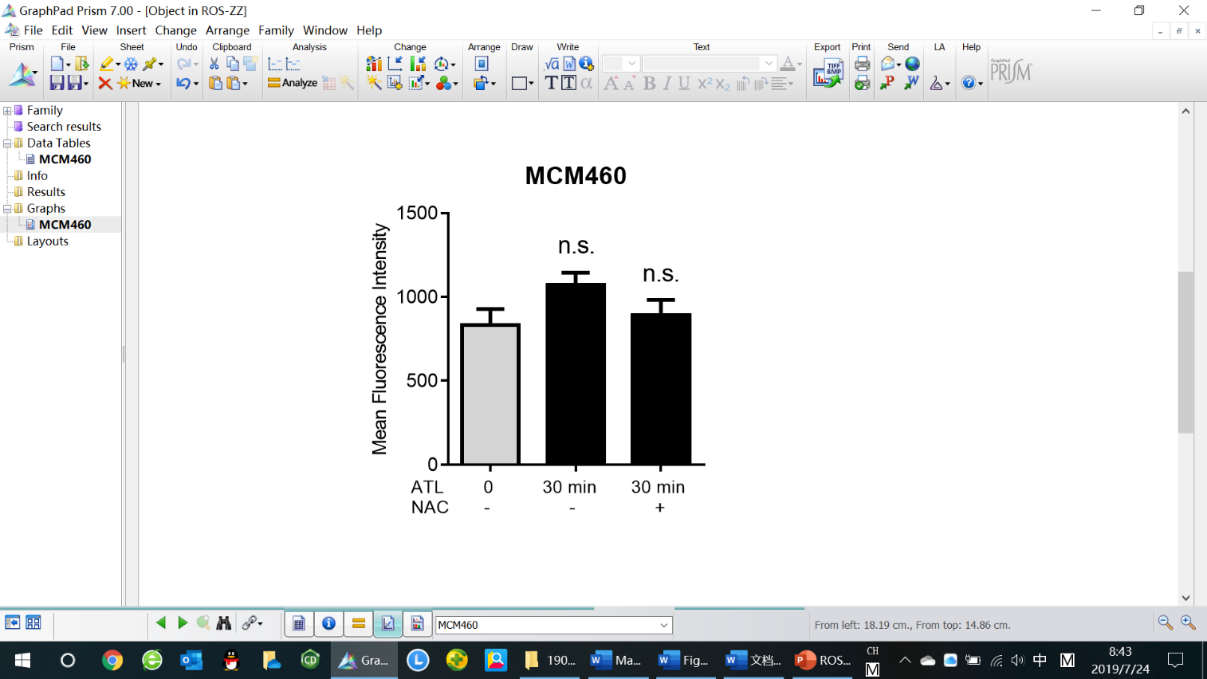

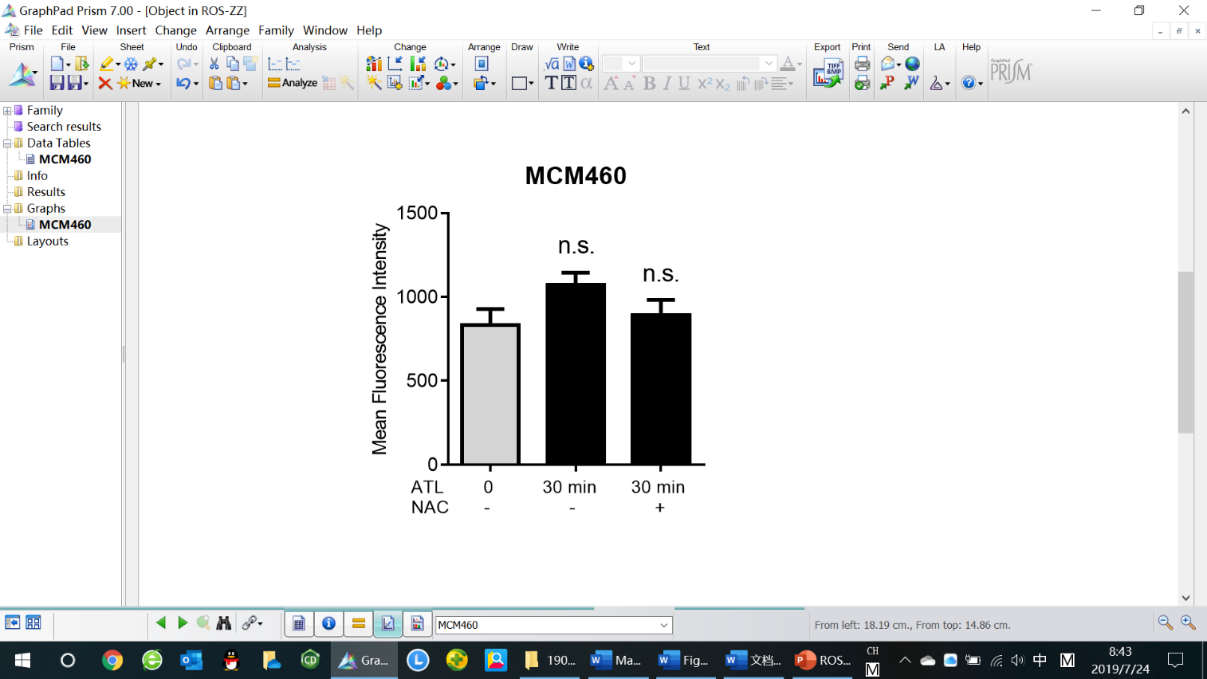

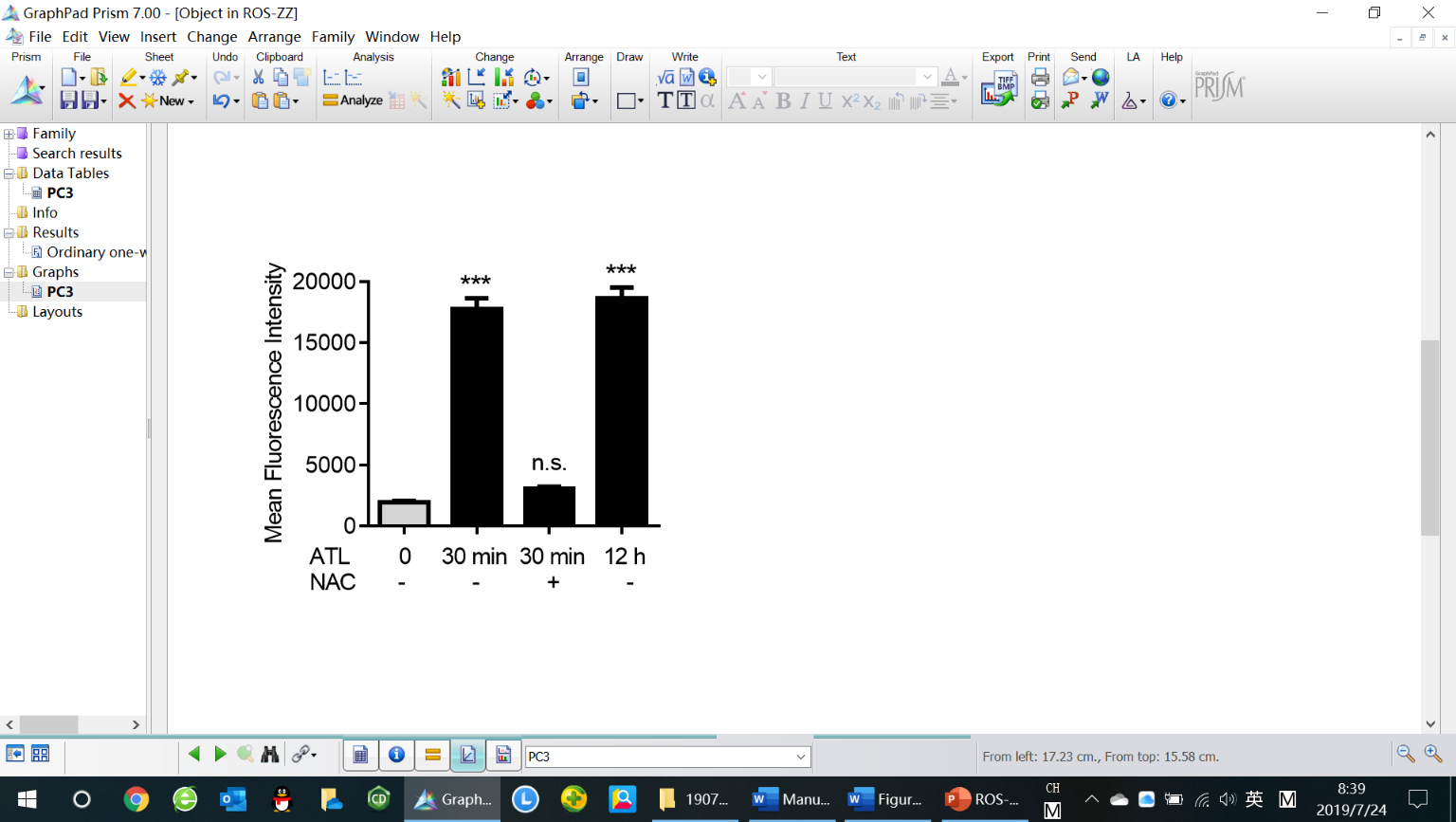

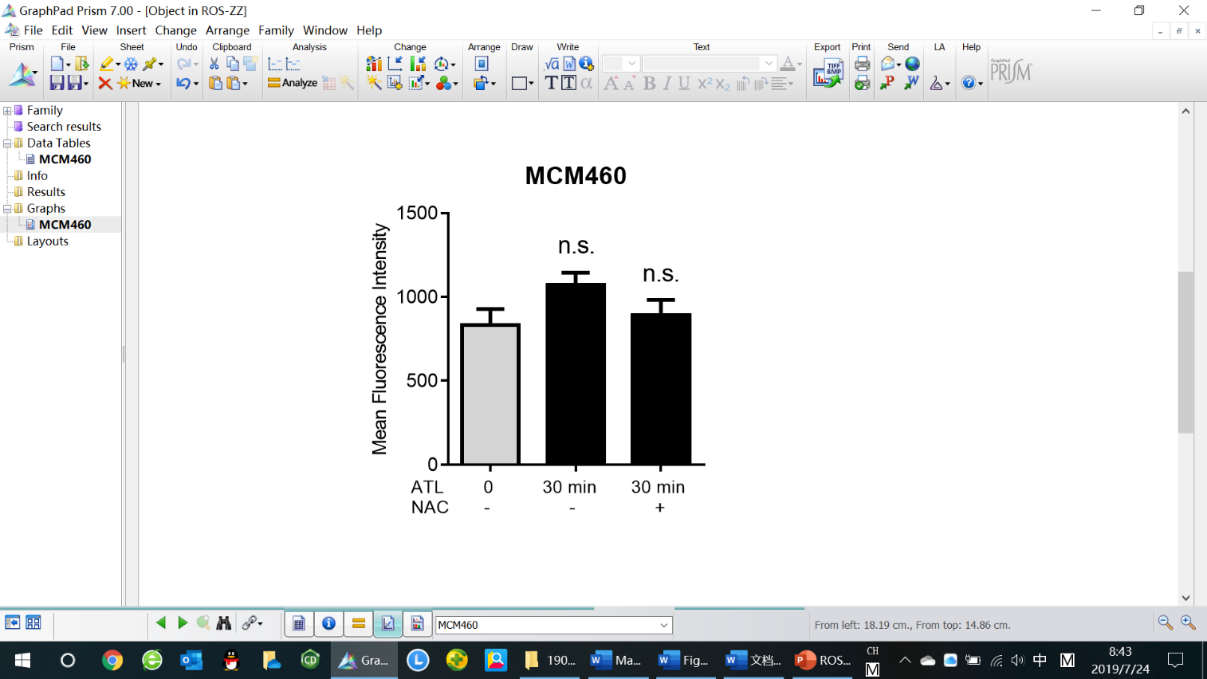

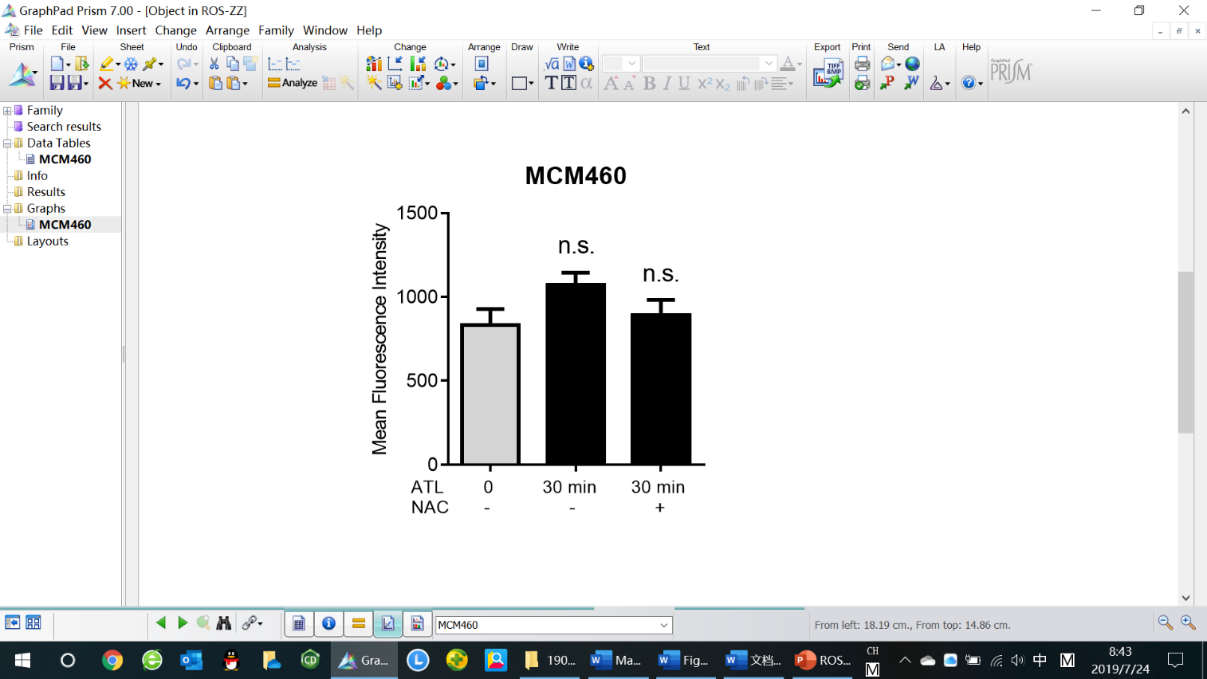


**Figure S5**
