# Supplementary material for: Synergistic lethality between PARP-trapping and alantolactone-induced oxidative DNA damage in homologous recombination-proficient cancer cells
Source: Oncogene. 2020 Feb 6;39(14):2905–20. doi: 10.1038/s41388-020-1191-x (PMC7118026; doi:10.1038/s41388-020-1191-x)
Supplement: Supplementary file 9 — Supplementary figure 8 [file 41388_2020_1191_MOESM9_ESM.docx]

**A**

**B**


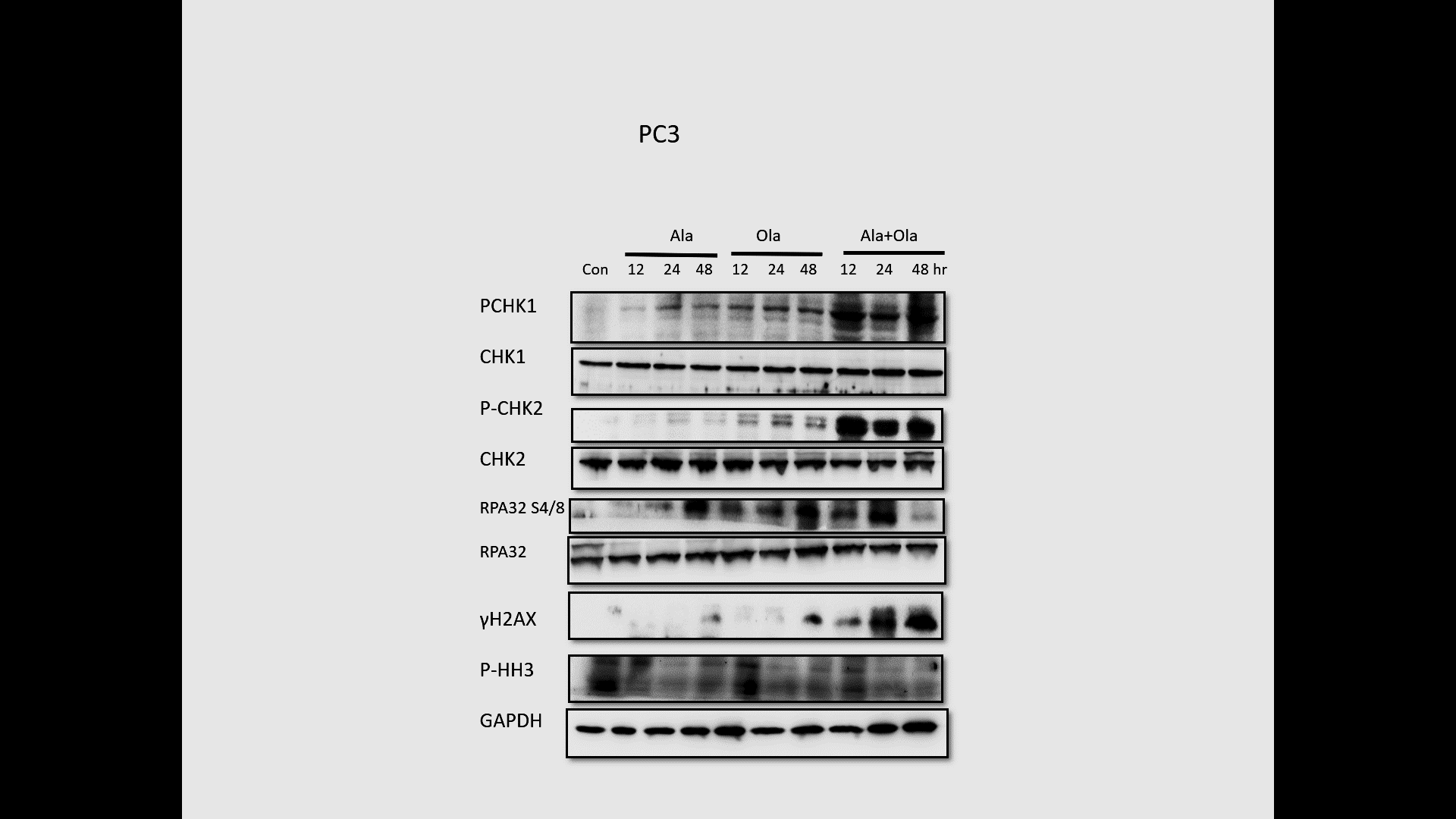


GAPDH

H3-pS10

12 h

24 h

48 h

12 h

24 h

48 h

12 h

24 h

48 h

control

ATL

Ola

ATL + Ola


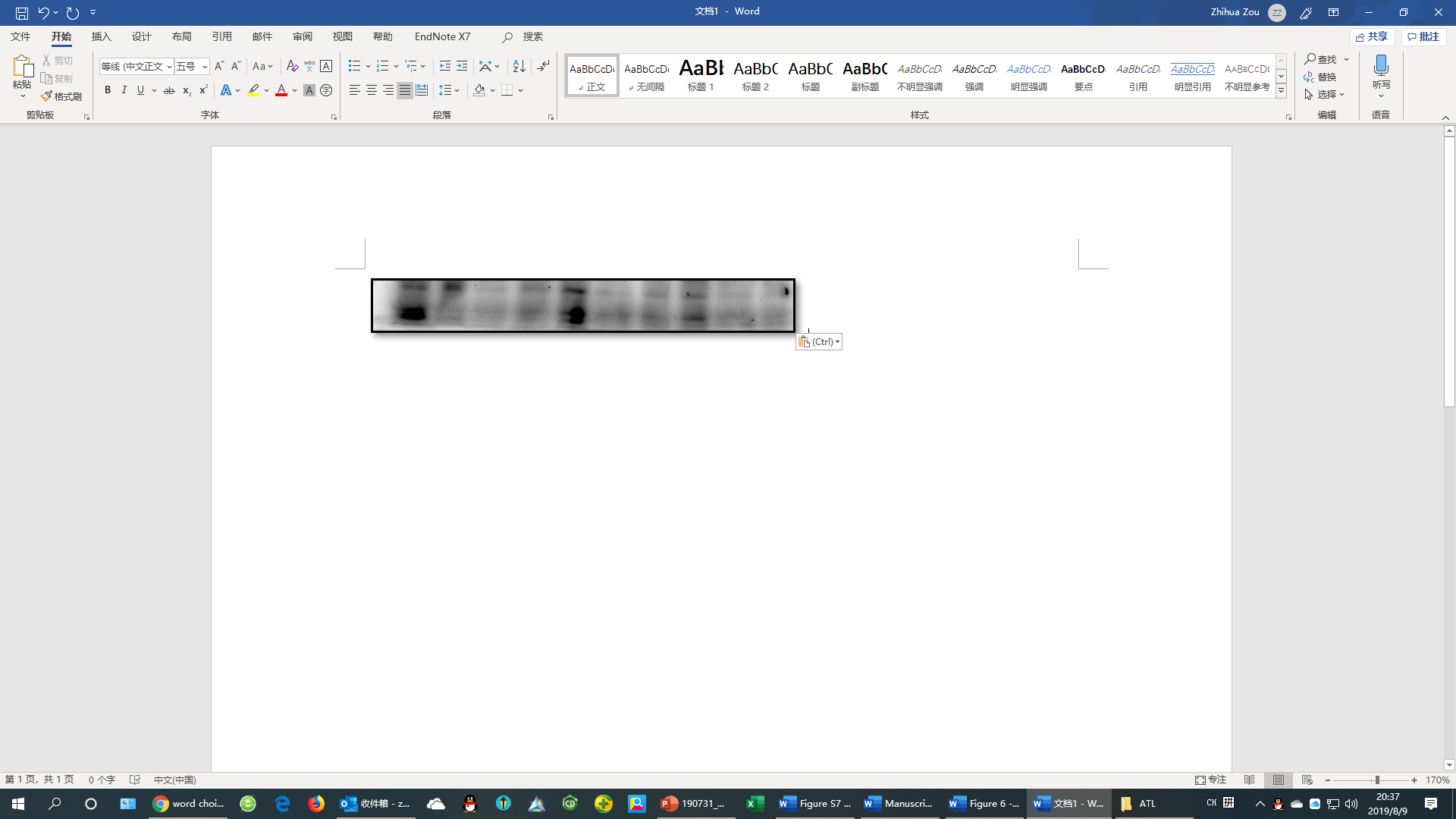

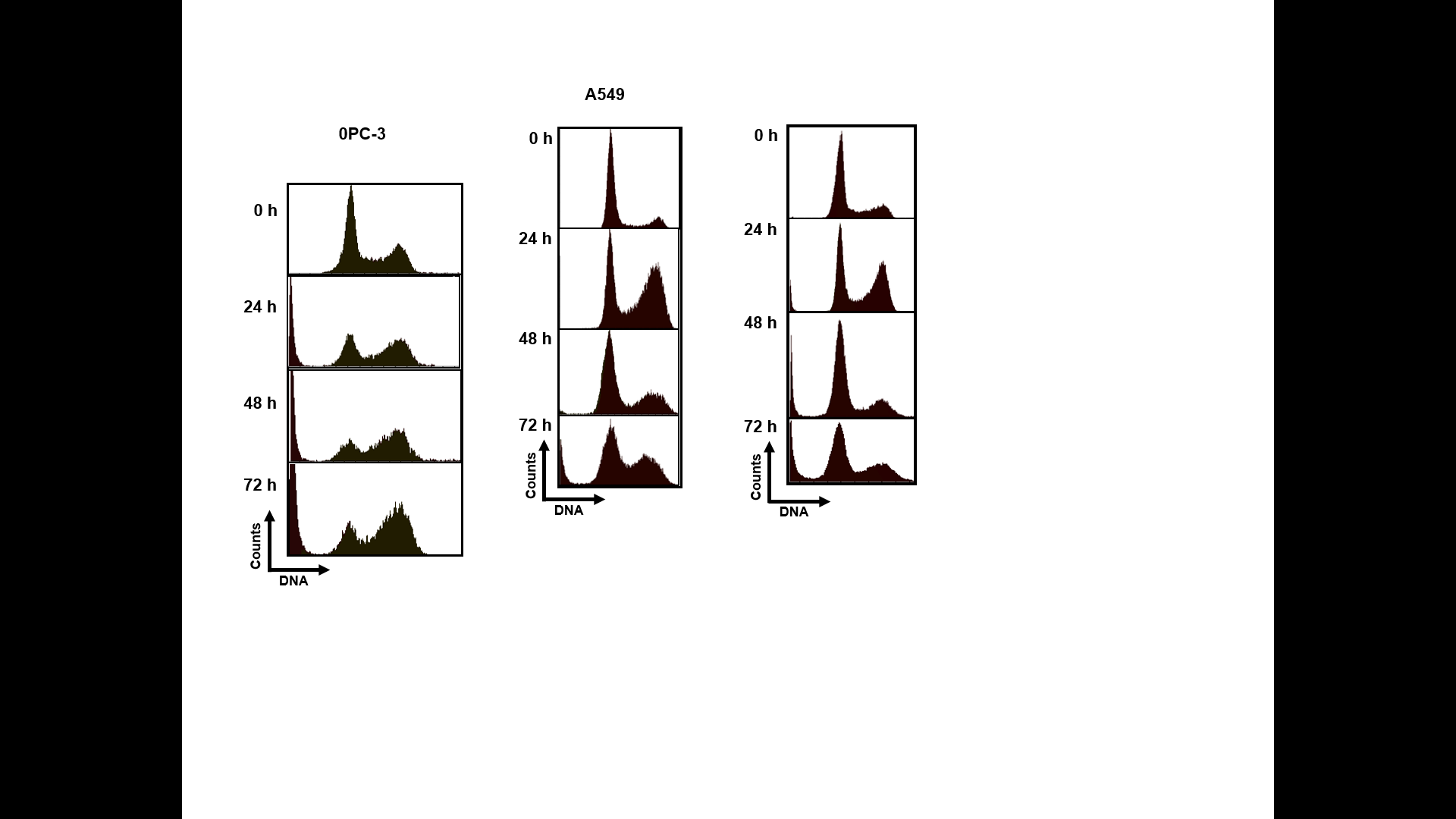


**SW480**


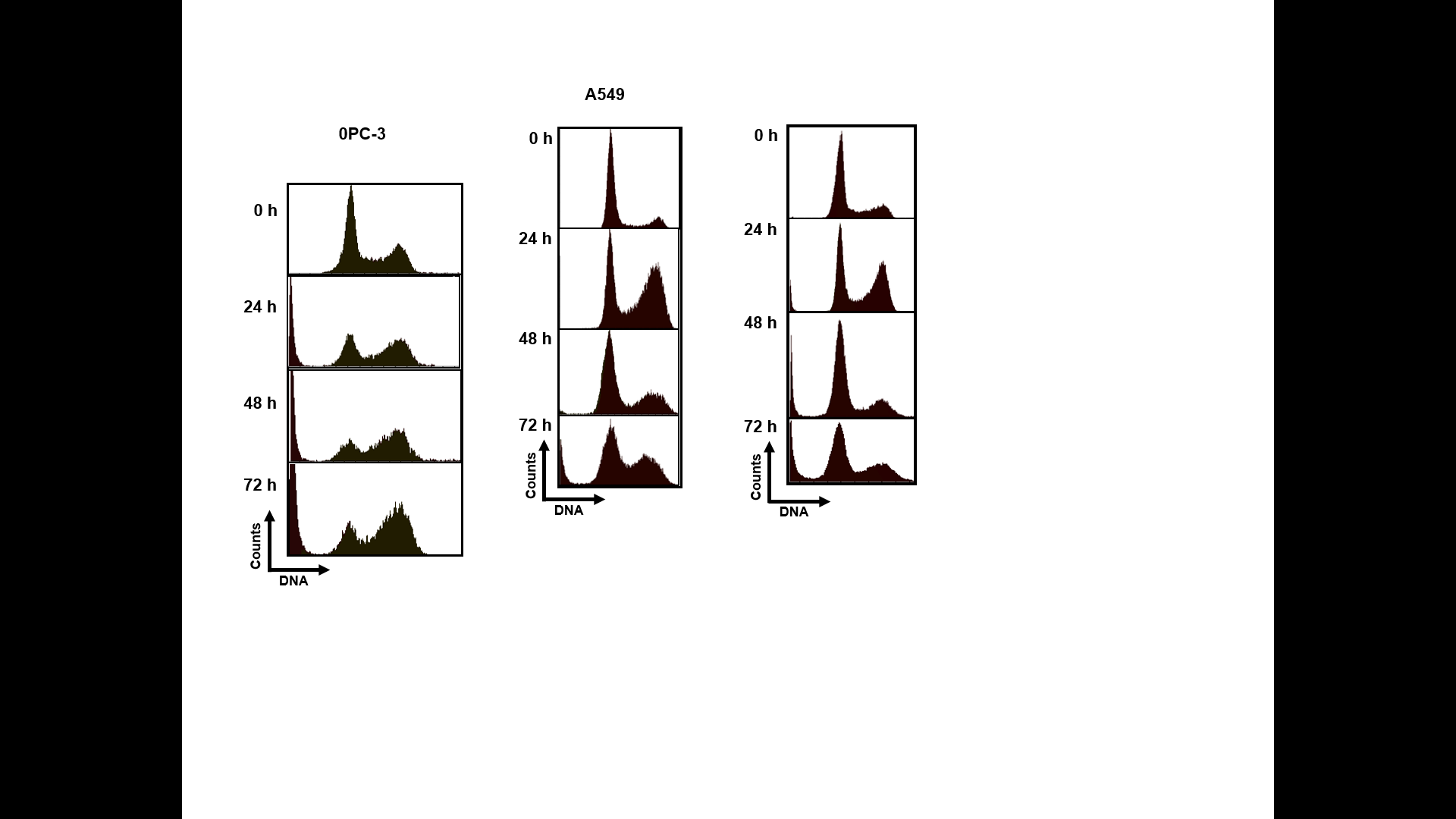


**A549**


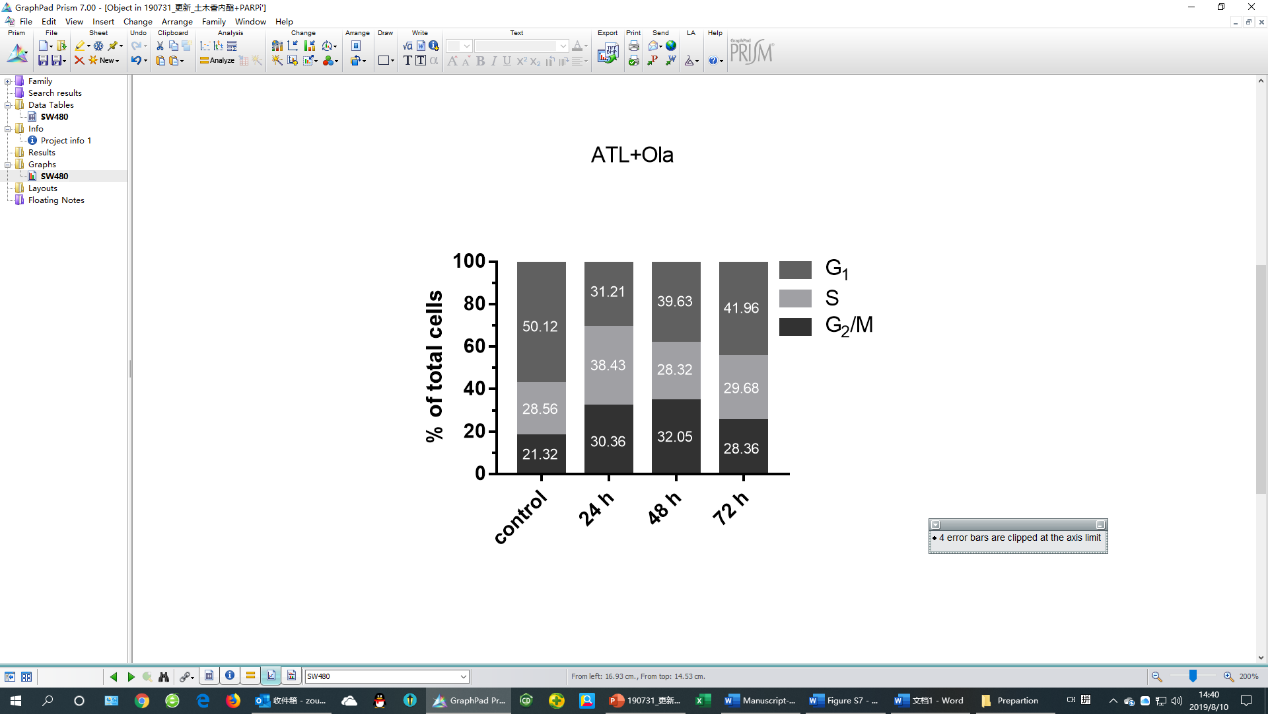

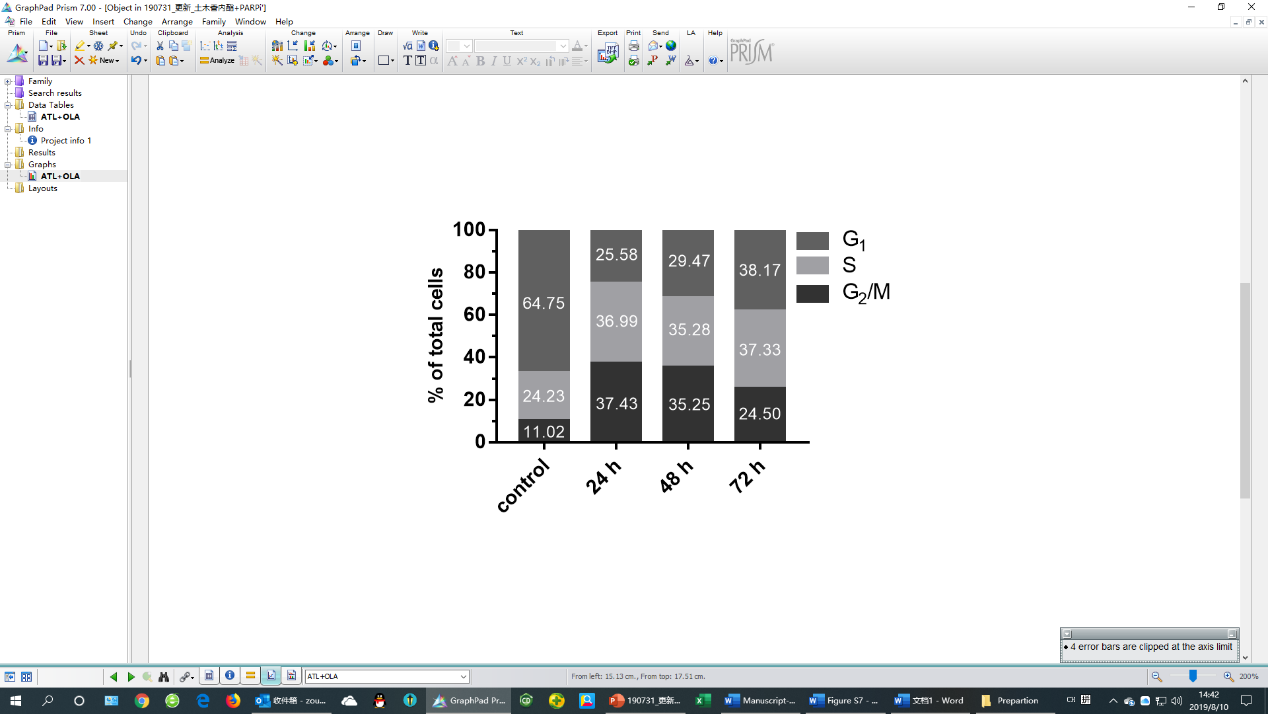


**A549**

**SW480**

**C**


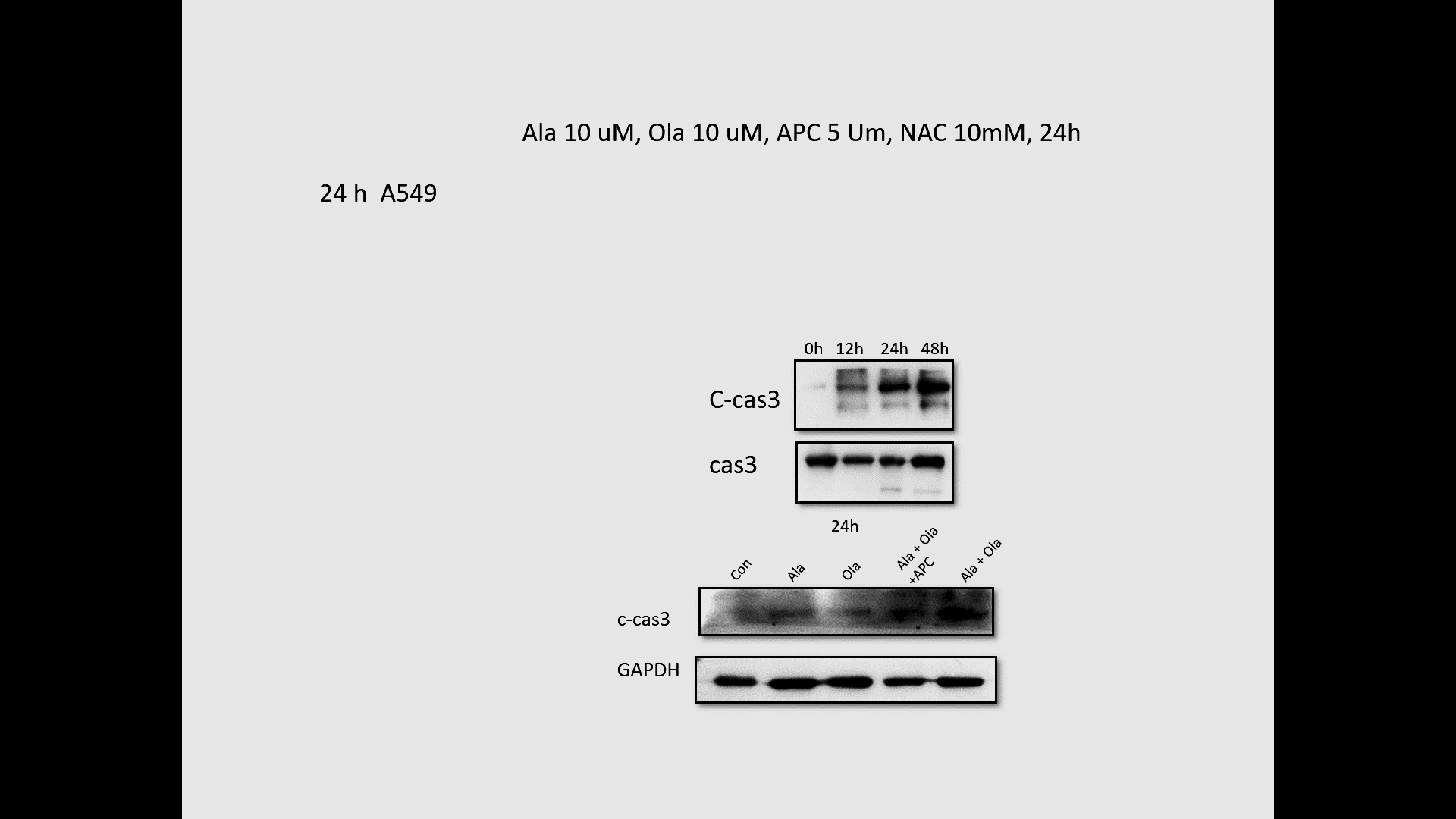

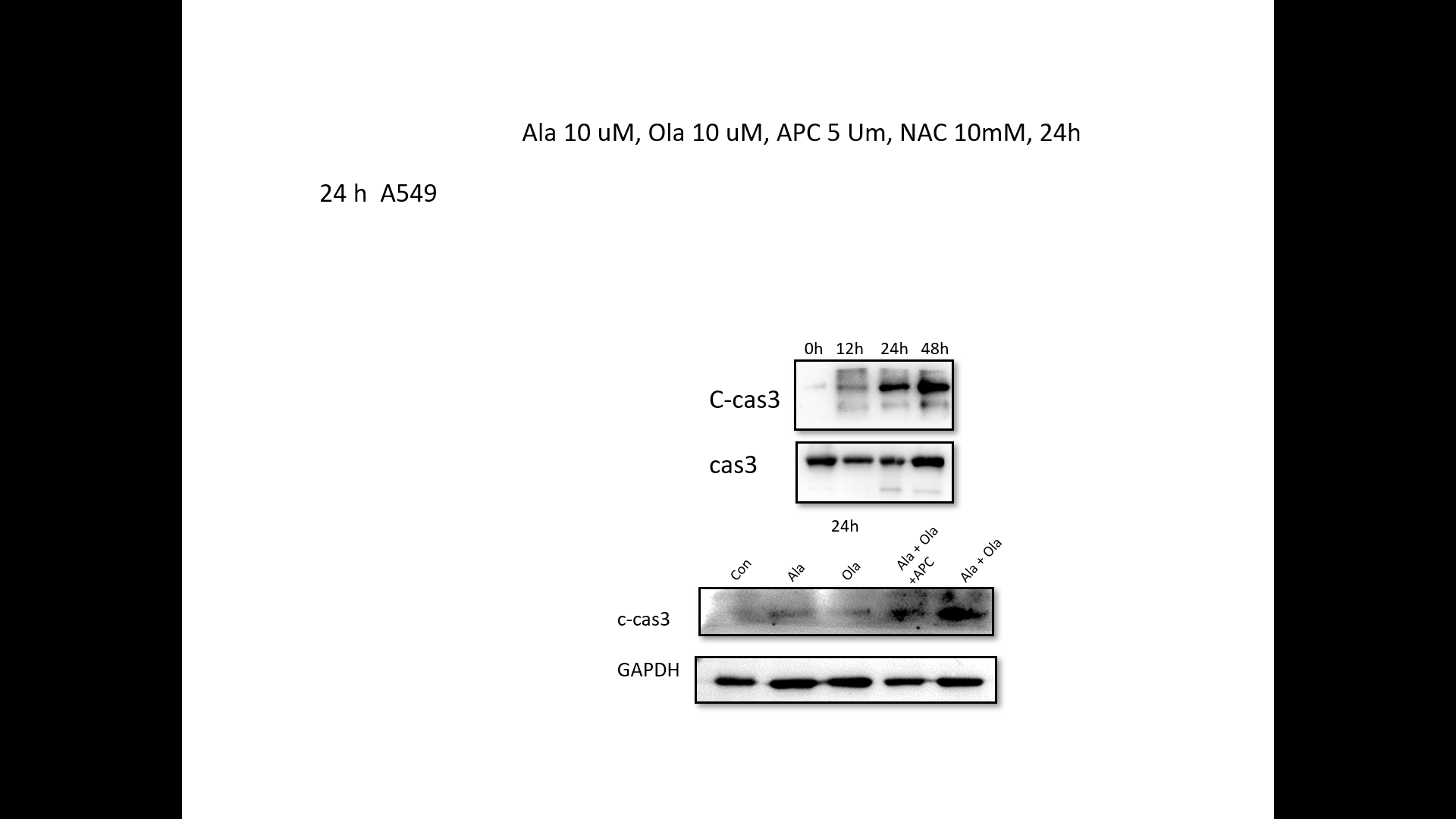

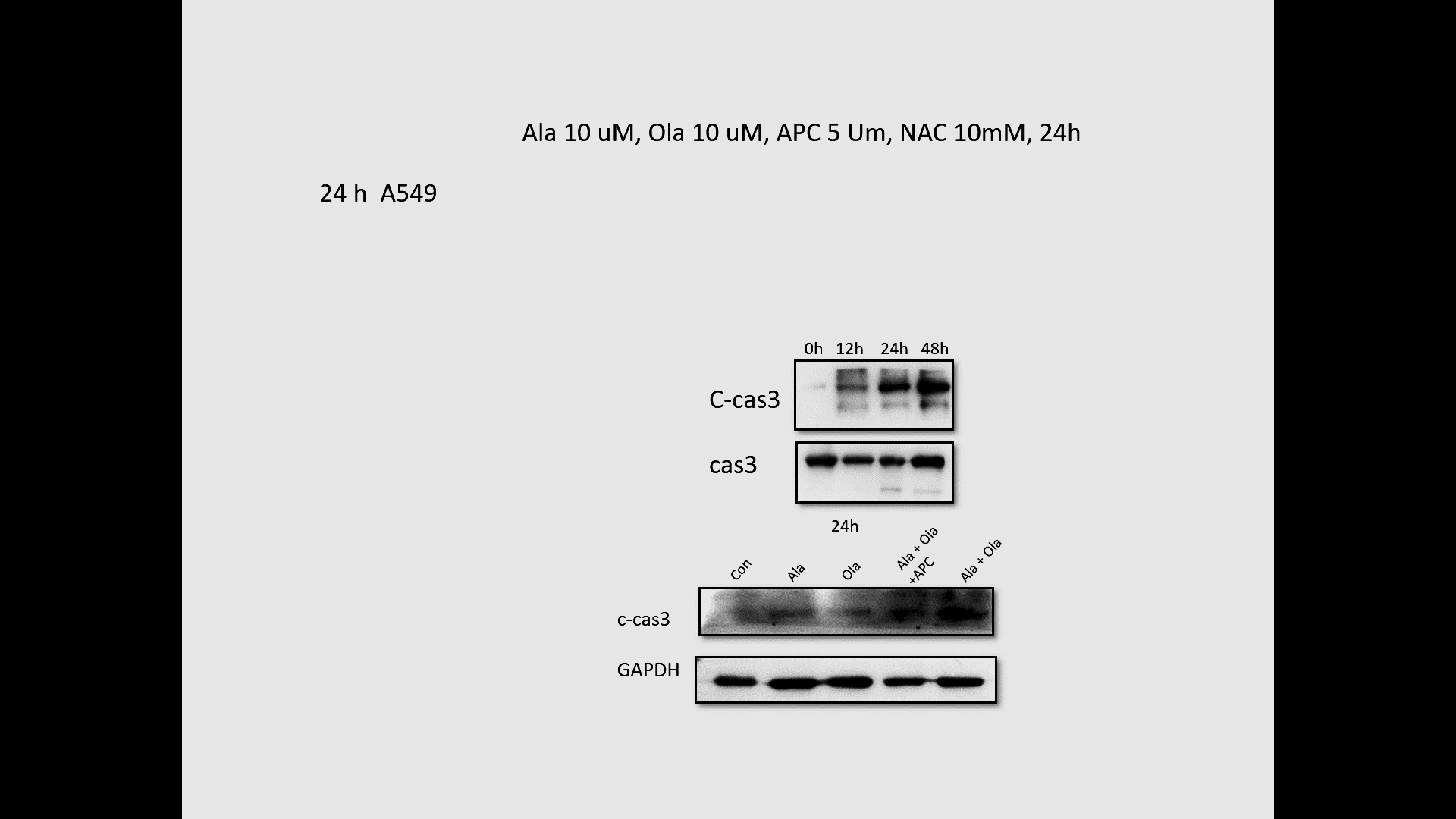


GAPDH

Caspase 3

Cleaved

Caspase 3

A549, ATL + Ola

0 h

12 h

24 h

48 h


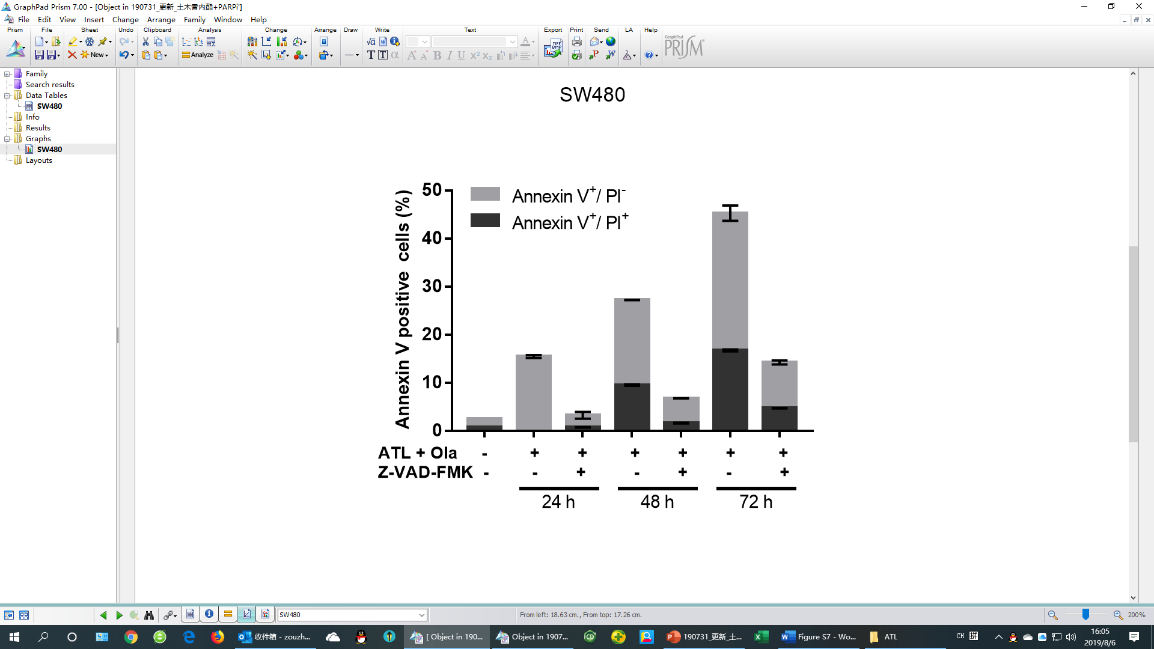


**SW480**


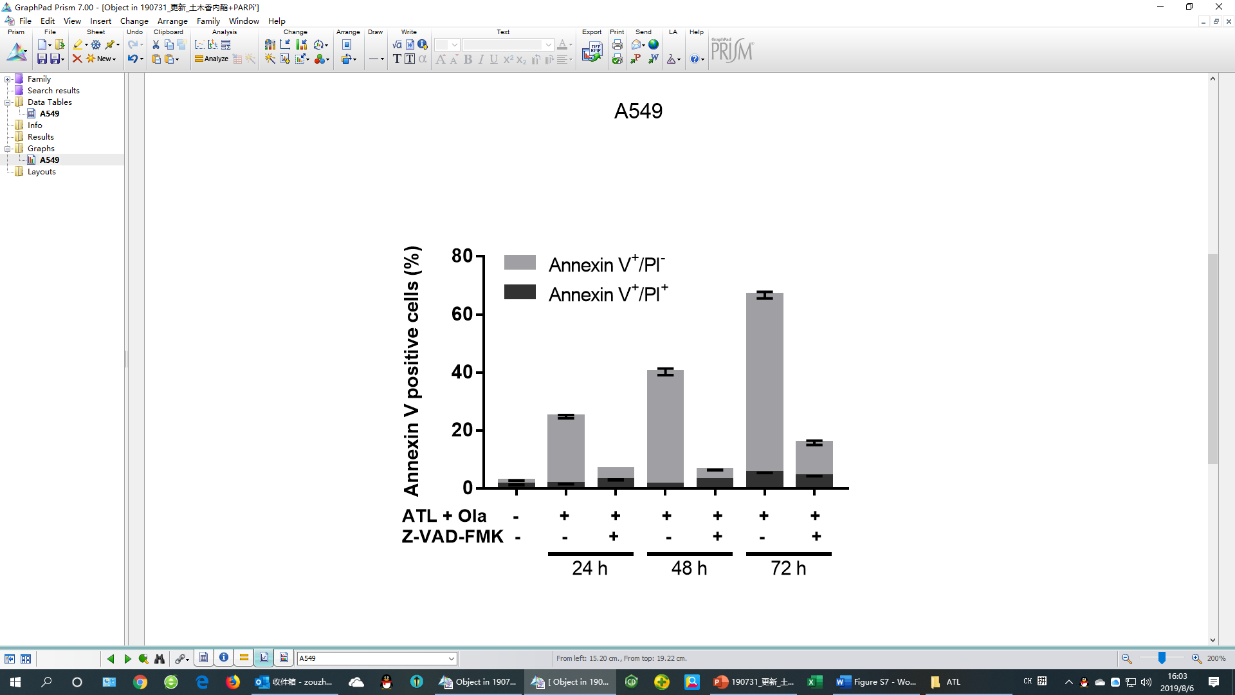


**A549**

**D**

**A549**

**Figure S8**
